# Epigenetic Activation of CCDC183‐AS1 Promotes Osteoclastogenesis and Prostate Cancer Bone Metastasis Through the FUBP1/LIGHT Axis

**DOI:** 10.1002/advs.202413288

**Published:** 2025-07-20

**Authors:** Chuandong Lang, Xiangyu Mu, Kun Chen, Xinwen Wang, Yuluo Rong, Jia Wang, Zongcheng Yang, Chi Yin, Yuhu Dai, Jun Xiao, Wenzhi Zhang

**Affiliations:** ^1^ Department of Orthopedics The First Affiliated Hospital of USTC Division of Life Sciences and Medicine University of Science and Technology of China Hefei Anhui 230001 China; ^2^ Department of Urology The First Affiliated Hospital of USTC Division of Life Sciences and Medicine University of Science and Technology of China Hefei Anhui 230001 China; ^3^ Department of Orthopaedic Surgery Sun Yat‐sen University First Affiliated Hospital Guangzhou Guangdong 510080 China; ^4^ Department of Stomatology The First Affiliated Hospital of USTC Division of Life Sciences and Medicine University of Science and Technology of China Hefei Anhui 230001 China; ^5^ Department of Orthopedics The Second Xiangya Hospital Central South University Changsha Hunan 410000 China

**Keywords:** bone metastasis, CCDC183‐AS1, FUBP1, KDM5C, LIGHT, osteoclastogenesis, prostate cancer

## Abstract

Bone metastasis (BM) is a major contributor to poor prognosis of prostate cancer (PCa); however, the underlying mechanisms of PCa BM remain poorly understood. A better understanding of these processes may provide critical insights for developing effective preventive and therapeutic strategies for PCa BM. In this study, significant upregulation of CCDC183‐AS1 in PCa BM is identified, which is associated with disease progression. CCDC183‐AS1 overexpression enhanced the ability of PCa cells to spread to the bone by inducing osteoclastogenesis and aiding in the creation of a BM niche. Mechanistically, CCDC183‐AS1 interacted with FUBP1 and enhanced its stability by inhibiting JTV‐1‐mediated ubiquitination and degradation of FUBP1, which promoted the transcription of TNFSF14 (LIGHT). Copy number gain‐induced upregulation of KDM5C epigenetically enhanced CCDC183‐AS1 expression by recruiting TET1 to its promoter and promoting DNA demethylation. Significantly, the administration of the selective FUBP1 inhibitor, FUBP1‐IN‐1, is shown to effectively suppress CCDC183‐AS1‐induced PCa BM. These results shed light on the involvement of CCDC183‐AS1 in enhancing osteoclastogenesis and the underlying mechanism in facilitating PCa BM, offering a potential avenue for therapeutic interventions.

## Graphical abstract

CCDC183‐AS1 overexpression enhanced the ability of PCa cells to spread to the bone by inducing osteoclastogenesis. Mechanistically, CCDC183‐AS1 interacted with FUBP1 and enhanced its stability, which promoted the transcription of TNFSF14 (LIGHT). Copy number gain‐induced KDM5C epigenetically upregulated CCDC183‐AS1 expression by recruiting TET1 to the promoter of CCDC183‐AS1. Significantly, FUBP1‐IN‐1 could effectively suppress CCDC183‐AS1‐induced PCa BM.

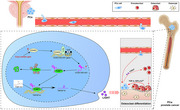

## 1 Introduction

Globally, prostate cancer (PCa) ranks as the second most prevalent malignancy in men. While the 5‐year survival rate for localized PCa is perfect at 100%, nearly 90% of men with advanced PCa develop bone metastases (BM), leading to a drastically lower 5‐year survival rate of under 30%.^[^ ^1^, ^2^, ^3^ ^]^ Cancer cells often metastasize to specific organs, and the main metastatic site of PCa is the bone.^[^ ^4^ ^]^ BM can lead to bone‐related complications, resulting in a reduced quality of life and poor prognosis.^[^ ^5^ ^]^ Unfortunately, the therapeutic interventions for PCa with BM are limited. Although earlier studies have highlighted the significant functions of proteins and non‐coding RNAs in BM of PCa, their precise molecular mechanisms and clinical applications are still not fully understood.^[^ ^6^ ^]^ Therefore, there is an urgent need to further understand the specific molecular mechanisms and develop innovative treatment strategies for PCa‐BM.

The spread of prostate cancer cells to the bone is a multifaceted and sequential process that includes leaving the original tumor, traveling through the bloodstream, settling in distant organs, and then multiplying to create metastatic growths.^[^ ^3^ ^]^ The regulation of bone homeostasis involves a dynamic equilibrium between bone resorption by osteoclasts (OCs) and bone formation by osteoblasts.^[^ ^7^ ^]^ However, many molecules secreted by PCa cells could disrupt this balance, creating a “BM niche” within the bone microenvironment that facilitates cancer metastasis.^[^ ^8^, ^9^ ^]^ Osteoclastogenesis, the process by which mature OCs are formed, consists of three main stages: commitment (hematopoietic stem cell differentiation into OC precursors), fusion (formation of multinucleated OCs), and activation (cytoskeletal polarization and podosome development).^[^ ^10^, ^11^, ^12^ ^]^ Numerous studies have demonstrated that PCa‐generated cytokines directly or indirectly stimulate osteoclastogenesis, leading to bone resorption.^[^ ^10^, ^13^ ^]^ In turn, this triggers a harmful cycle where OCs secrete enzymes such as tartrate‐resistant acid phosphatase (TRAP) and matrix metalloproteinases, leading to the breakdown of the bone matrix and encouraging tumor cell proliferation and spread.^[^ ^14^ ^]^ Consequently, understanding the crosstalk between PCa cells and OCs is crucial for developing novel therapeutic strategies targeting BM.

Long noncoding RNAs (lncRNAs) are naturally occurring RNA molecules exceeding 200 nucleotides in length, which have little to no ability to code for proteins. Studies have demonstrated their essential role in numerous core biological functions and illnesses.^[^ ^15^ ^]^ At the molecular level, lncRNAs can function as decoys by binding to proteins and microRNAs, or serve as scaffolds and guides, thereby modulating interactions among proteins, DNA, and other RNA molecules. Consequently, they can control gene expression at various stages, including epigenetics, transcription, post‐transcription, translation, and post‐translation.^[^ ^16^, ^17^, ^18^ ^]^ With advances in biological technologies, increasing evidence has demonstrated that lncRNAs play crucial roles in tumor metastasis. Tian et al. found that lncRNA VAL, which is directly activated by AKT/STAT3 signaling, is an effective pro‐metastatic molecule necessary for AKT‐ driven tumor metastasis, and antitumor responses in lung adenocarcinoma.^[^ ^19^ ^]^ In PCa, Wang et al. showed that lncRNA TMPO‐AS1 can regulate the Wnt/β‐catenin signaling pathway through the CSNK2A1/DDX3X complex, thereby promoting PCa BM.^[^ ^20^ ^]^ Therefore, lncRNAs could act as indicators for prognosis and diagnosis, offering fresh perspectives on treating human ailments, such as cancer. Nonetheless, to the best of our knowledge, research on lncRNAs in PCa BM and their impact on bone homeostasis remains limited.

FUBP1, far upstream element‐binding protein 1, is a versatile protein that binds to both DNA and RNA, playing a role in numerous cellular activities including transcription, translation, and RNA splicing.^[^ ^21^ ^]^ It is widely recognized that FUBP1 can form complexes with the far upstream element (FUSE) site to regulate gene expression, including key genes such as c‐Myc, P21, and P53.^[^ ^22^, ^23^ ^]^ Current research indicates that FUBP1 is upregulated in various tumor types, including renal cell carcinoma, squamous cell carcinoma, non‐small cell lung cancer, gastric cancer, breast cancer, liver cancer, and Hodgkin's lymphoma.^[^ ^24^ ^]^ As an oncogene, FUBP1 promotes tumor cell proliferation and metastasis while inhibiting apoptosis. It has been reported that FUBP1 enhances the development and metastasis of colorectal cancer via DVL1‐mediated activation of the Wnt/β‐catenin signaling pathway.^[^ ^25^ ^]^ Xiong et al. demonstrated that tRNA‐Val‐Ca2 promotes pancreatic cancer metastasis by interacting with FUBP1 and activating c‐MYC transcription.^[^ ^26^ ^]^ So far, the link between FUBP1 levels and PCa BM has not been investigated.

In this study, we utilized a public database to identify the key lncRNAs driving PCa BM. CCDC183‐AS1 was selected and investigated for its role in PCa BM progression. Notably, our study revealed that CCDC183‐AS1 was elevated in PCa BM tissues and correlated with a worse prognosis for the disease. Functionally, CCDC183‐AS1 promoted osteoclastogenesis in vitro and PCa BM in vivo. Mechanistically, CCDC183‐AS1 competes with JTV‐1 to bind FUBP1, enhancing FUBP1 stability, which in turn stimulates the transcription of LIGHT, leading to an imbalance in bone homeostasis and facilitating the metastasis of PCa cells to the bone. Additionally, we demonstrated that the copy number gain‐induced elevation of KDM5C promotes CCDC183‐AS1 expression by modulating DNA demethylation in a TET1‐dependent manner. The innovation of this study lies in being the first to demonstrate the role of the CCDC183‐AS1/FUBP1/LIGHT signaling axis in osteoclastogenesis and PCa bone metastasis. It also highlights the therapeutic potential of targeting this signaling axis in the treatment of PCa bone metastasis. Previous studies about PCa bone metastasis have primarily focused on the enhanced migratory and invasive capacity of PCa cells as a determinant of metastatic potential. In contrast, this study broadens the mechanistic understanding of bone metastasis by elucidating the interaction between the bone microenvironment and tumor cells, offering a novel therapeutic avenue for targeting the bone microenvironment in the treatment of PCa bone metastasis.

## 2 Results

### 2.1 CCDC183‐AS1 Overexpression is Related to BM in PCa

**Figure 1:**
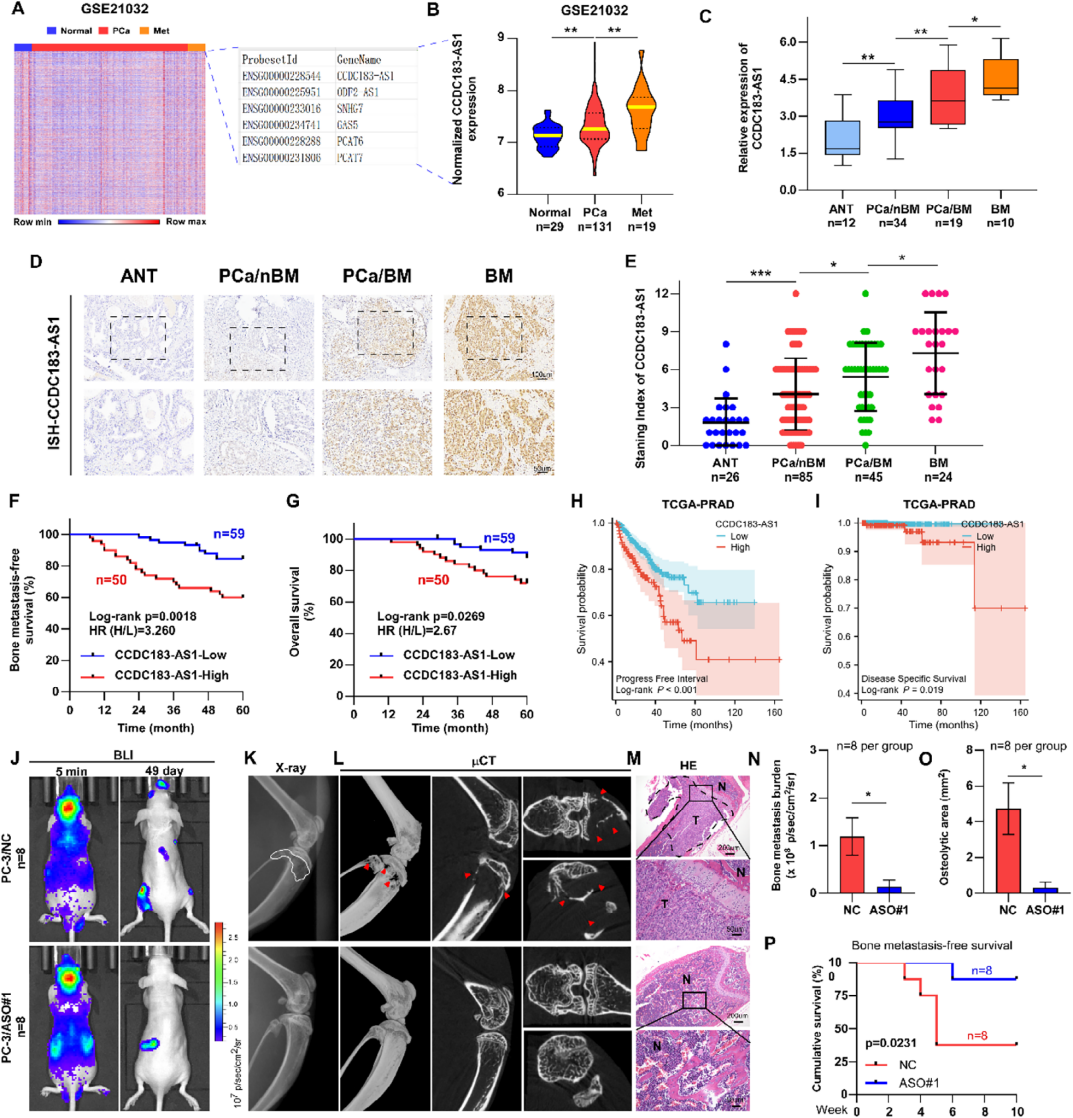
CCDC183‐AS1 overexpression is related to BM in PCa. A) Heatmap showing differentially expressed lncRNAs among normal prostate tissues (Normal), primary PCa (PCa), and metastatic PCa tissues (Met) (GSE21032 dataset). B) Expression of CCDC183‐AS1 in normal prostate tissues (n = 29), primary PCa tissues (n = 131), and metastatic PCa tissues (n = 19) within the GSE21032 dataset. C) RT‐qPCR analysis of CCDC183‐AS1 expression in adjacent normal tissues (ANT) (n = 12), PCa without BM (PCa/nBM) (n = 34), PCa with BM (PCa/BM) (n = 19), and BM (n = 10) tissues. D,E) Representative ISH images (D) and quantitative analysis (E) of CCDC183‐AS1 expression in ANT (n = 26), PCa/nBM (n = 85), PCa/BM (n = 45), and BM tissues (n = 24). Bars, 100 and 50 um. F) Kaplan‐Meier analysis of bone metastasis‐free survival in PCa patients with low and high CCDC183‐AS1 expression based on our clinical cohort. H, high; L, low. G) Kaplan‐Meier analysis of overall survival in PCa patients with low and high CCDC183‐AS1 expression based on our clinical cohort. H, high; L, low. H) Kaplan‐Meier analysis of progression‐free interval in PCa patients with low and high CCDC183‐AS1 expression based on the TCGA‐PRAD cohort. I) Kaplan‐Meier analysis of disease‐specific survival in PCa patients with low and high CCDC183‐AS1 expression based on the TCGA‐PRAD cohort. J) Representative BLI images of bone metastasis lesions in mice. K) Representative X‐ray images of bone metastasis lesions in mice. L) Representative µCT images of bone metastasis lesions in mice. M) Representative HE images of bone metastasis lesions in mice. Bars, 200 and 50 um. N) Quantification of bone metastasis burden based on BLI. O) Quantification of osteolytic areas based on X‐ray. P) Kaplan‐Meier analysis showing bone metastasis‐free survival curve of mice from two groups. ASOs (10 nmol in 100 µl PBS) or PBS (100 µl) were administered via tail vein injection every 5 days for a total of 6 doses after injection with PC‐3 cells. All experiments were performed in biological triplicate. Statistical analyses were performed by Student's t‐test (N, O) and one‐way ANOVA test (B, C, E), and the log‐rank test (F, G, H, I, P). *p < 0.05, **p < 0.001, and ***p < 0.0001.

In order to identify the key lncRNAs that promote PCa metastasis, we conducted a differential analysis of normal, primary PCa, and metastatic PCa tissues using data from the GSE21032 dataset. As shown in **Figure** **1**A,B, compared to normal prostate tissues, six lncRNAs (CCDC183‐AS1, ODF2‐AS1, SNHG7, GAS5, PCAT6, and PCAT7) were significantly upregulated in primary PCa tissues and were further elevated in metastatic PCa tissues. The analysis based on the prostate adenocarcinoma database from The Cancer Genome Atlas (TCGA‐PRAD) revealed that these five lncRNAs, excluding ODF2‐AS1, also exhibited a differential expression trend in PCa relative to adjacent normal tissues (ANT), which further indicated their oncogenic role in PCa (Figure S1A, Supporting Information). Given that the biological function of the aforementioned four lncRNAs except CCDC183‐AS1 in PCa has been reported, we chose CCDC183‐AS1 for further research.

In PCa, the main site for metastasis is the bone. Consequently, we analyzed CCDC183‐AS1 expression pattern specifically in PCa BM. Initially, the differential expression analysis based on the TCGA‐PRAD database demonstrated that CCDC183‐AS1 was significantly higher in PCa with BM (PCa/BM) than in PCa without BM (PCa/nBM) tissues (Figure S1B, Supporting Information). Consistently, RT‐qPCR and ISH analyses illustrated that compared with ANT or PCa/nBM, CCDC183‐AS1 was overexpressed in PCa/BM tissues and further increased in BM tissues (Figure 1C–E). ISH in 12 paired patient samples showed that CCDC183‐AS1 expression was increased in PCa BM tissues, as compared with the corresponding primary PCa tissues (Figure S1C, Supporting Information). Our clinical cohort's survival analysis revealed that individuals exhibiting high levels of CCDC183‐AS1 had worse overall survival and BM‐free survival compared to those with low CCDC183‐AS1 expression (Figure 1F,G). Similarly, an examination of the TCGA‐PRAD cohort indicated that patients with elevated CCDC183‐AS1 expression experienced shorter progression‐free interval and disease‐specific survival compared to those with reduced CCDC183‐AS1 levels (Figure 1H,I). Furthermore, high CCDC183‐AS1 expression was associated with poorer clinicopathological characteristics of PCa patients both in TCGA and our cohorts (Figure S1D–K, Supporting Information). Collectively, this evidence indicates a strong association between CCDC183‐AS1 overexpression and BM in PCa.

Consistently, CCDC183‐AS1 expression in PCa cell lines was significantly upregulated compared to that in normal prostate cell line (RWPE‐1) (Figure S1L, Supporting Information). To further determine whether CCDC183‐AS1 influences PCa BM, we used antisense oligonucleotides (ASOs) targeting CCDC183‐AS1 to silence CCDC183‐AS1 in PC‐3 cells and constructed two stable cell lines (DU145 and 22RV1) overexpressing CCDC183‐AS1 (Figure S1M, Supporting Information). Through intracardiac injection of PC‐3 cells to establish bone metastasis in vivo, we observed that treatment with ASO delayed and decreased BM onsets of mice, indicated by the reduced BM burden shown in BLI and H&E, and osteolytic lesions shown in X‐ray and µCT (Figure 1J–O). Survival analysis showed that treatment with ASO prolonged the BM‐free survival of mice (Figure 1P). Overall, our results demonstrated that CCDC183‐AS1 plays a critical role in PCa BM.

### 2.2 CCDC183‐AS1 Overexpression Promotes Osteoclastogenesis

**Figure 2:**
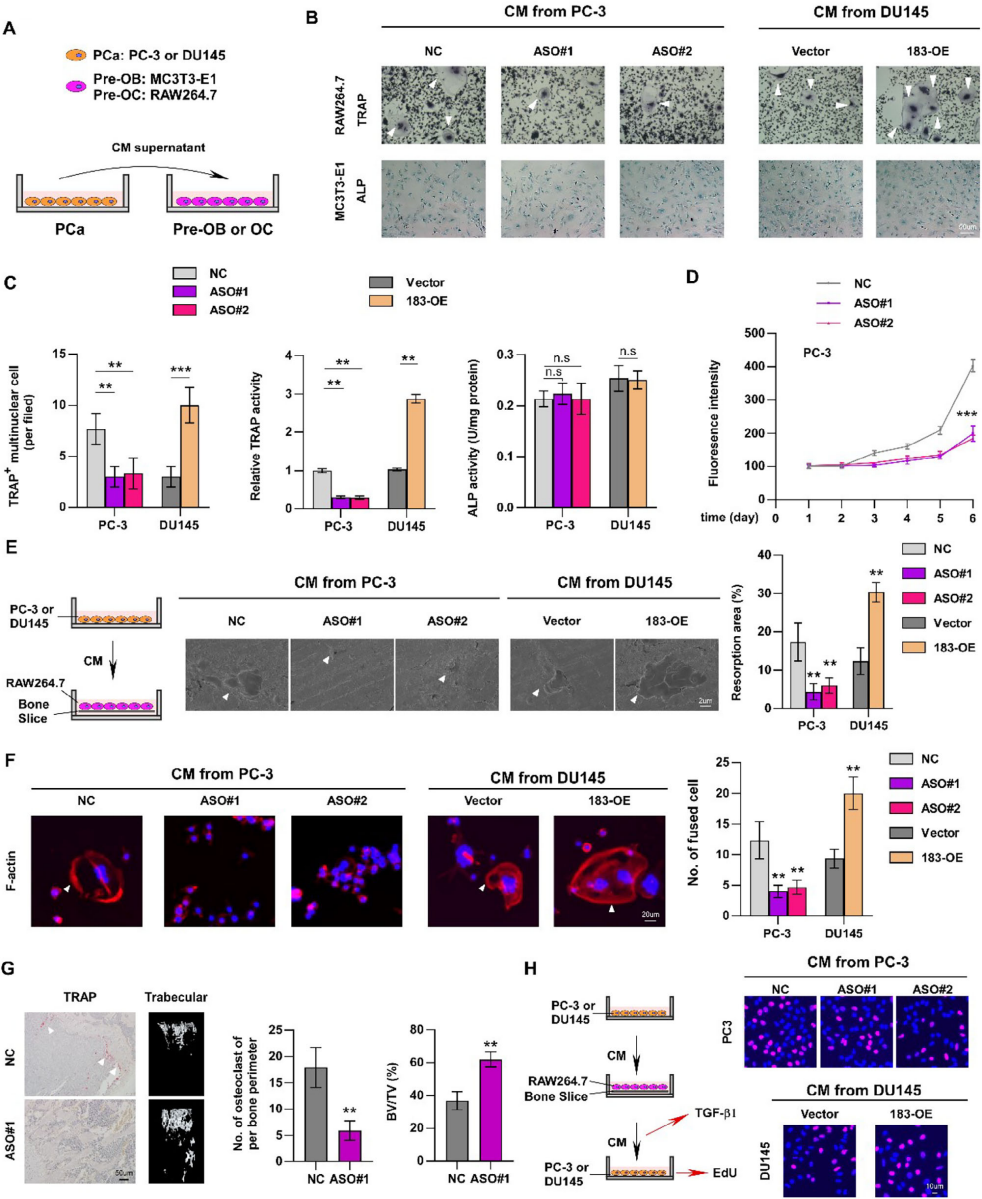
CCDC183‐AS1 overexpression promotes osteoclastogenesis. A) Schematic illustration of treatment of MC3T3‐E1 and RAW264.7 cells with conditioned medium (CM) from PCa cells. OB, osteoblast; OC, osteoclast. B) Osteoclast differentiation assay using TRAP staining (top) and osteoblast differentiation assay using ALP staining (bottom) after treatment with CM from the indicated PCa cells. 183‐OE, CCDC183‐AS1 overexpression. Bars, 50 um. C) Quantification of TRAP^+^ multinucleated OCs, TRAP activity, and ALP activity in the osteoclast and osteoblast differentiation assays. D) Measurement of fluorescence intensity of the culture supernatant of RAW264.7 cells treated with CM from PCa cell. E) Bone resorption assays of RAW264.7 cells cultured onto the bone slices for indicated treatments. Then bone slice was fixed for scanning electron microscopy (SEM) (left) and quantification of the area of resorption pit per bone slice (right). Bars, 2 um. F) Formation of actin rings (left) and quantification of fused multinucleated cells after treating RAW264.7 with CM from PCa cells. Bars, 20um. G) Representative TRAP staining and µCT (trabecular bone section) images of BM lesions in mice (left). Quantification of TRAP^+^ OCs along the bone‐tumor interface of metastases and bone volume fraction/total volume (BV/TV) in the indicated mice. Bars, 50 um. H) Schematic representation of the vicious cycle between cancer cells and OCs (upper). Representative EdU staining image of PCa cells with different treatments (lower). Bars, 10 um. All experiments were performed in biological triplicate. Statistical analyses were performed by Student's t‐test (G) and one‐way ANOVA test (C, D, E, F). *p < 0.05, **p < 0.001, and ***p < 0.0001.

The bone metastasis microenvironment is primarily regulated by OCs and osteoblasts,^[^ ^4^ ^]^ and since its dysregulation significantly influences BM, we further explored the effect of different levels of CCDC183‐AS1 expression on these cells. Pre‐osteoclast (RAW264.7) and pre‐osteoblast (MC3T3‐E1) cells were treated with conditioned medium (CM) obtained from PCa cells (**Figure** **2**A).The results of the osteoclast and osteoblast differentiation experiments indicated that treatment with CM derived from PCa cells with CCDC183‐AS1 silence by ASO dramatically decreased the number of TRAP^+^‐multinuclear OCs, while CM from CCDC183‐AS1‐overexpressed PCa cells elevated the count of TRAP^+^‐multinuclear OCs, demonstrating that CCDC183‐AS1 could regulate the capability of PCa cells to promote osteoclastogenesis in vitro (Figure 2B,C). However, CM from different PCa cells with altered CCDC183‐AS1 expression did not affect osteoblast differentiation (Figure 2B,C). The bone resorption assay consistently showed that CCDC183‐AS1 promoted osteoclast‐mediated bone resorption indicated by the fluorescence intensity in the medium (Figure 2D; Figure S2A, Supporting Information). Meanwhile, the CM from PCa cells in the CCDC183‐AS1 ASO treatment group markedly reduced the area of bone resorption pits (Figure 2E). In contrast, an increased area of bone resorption pits was observed in the CCDC183‐AS1 overexpression group (Figure 2E), suggesting that the CCDC183‐AS1‐induced secretome promoted the formation of a vicious cycle. Podosome formation is crucial for bone resorption and is regulated by multinuclear OCs to form actin‐rich sealing zones.^[^ ^14^ ^]^ We found that CM from ASO‐treated PCa cells inhibited podosome formation, whereas CM from PCa cells overexpressing CCDC183‐AS1 promoted podosome formation, as shown by the altered formation of actin rings in immunofluorescence (IF) staining (Figure 2F). Primary bone marrow macrophages (BMMs), widely recognized as osteoclast precursors, are frequently utilized in studies of osteoclastogenesis. Using BMMs, we also found that CM from ASO‐treated PCa cells inhibited osteoclastogenesis, bone resorption and podosome formation (Figure S2B–E, Supporting Information). Thus, the above findings demonstrated that PCa cells overexpressing CCDC183‐AS1 enhance osteoclast differentiation and activation. Following this, we further revealed that a decrease in TRAP^+^‐OCs and a notable rise in bone volume/total volume (BV/TV) and trabecular thickness (Tb.Th) in the ASO‐treated group through TRAP and µCT assays performed in hindlimbs from mice shown in Figure 1, suggesting that CCDC183‐AS1 might affect PCa cell‐induced osteoclastogenesis and bone destruction in vivo (Figure 2G; Figure S2F, Supporting Information). More importantly, enhanced osteoclastogenesis induced by CM derived from CCDC183‐AS1‐overexpressing PCa cells apparently led to elevated bone resorption levels, indicated by an increase in TGF‐β1 released from the bone matrix, which in turn promoted PCa cell proliferation, further exacerbated the vicious cycle between PCa cells and OCs (Figure 2H; Figure S2G,H, Supporting Information). In summary, these results indicate that elevation of CCDC183‐AS1 in PCa cells promotes osteoclastogenesis.

### 2.3 CCDC183‐AS1 Interacts with FUBP1 Protein

**Figure 3:**
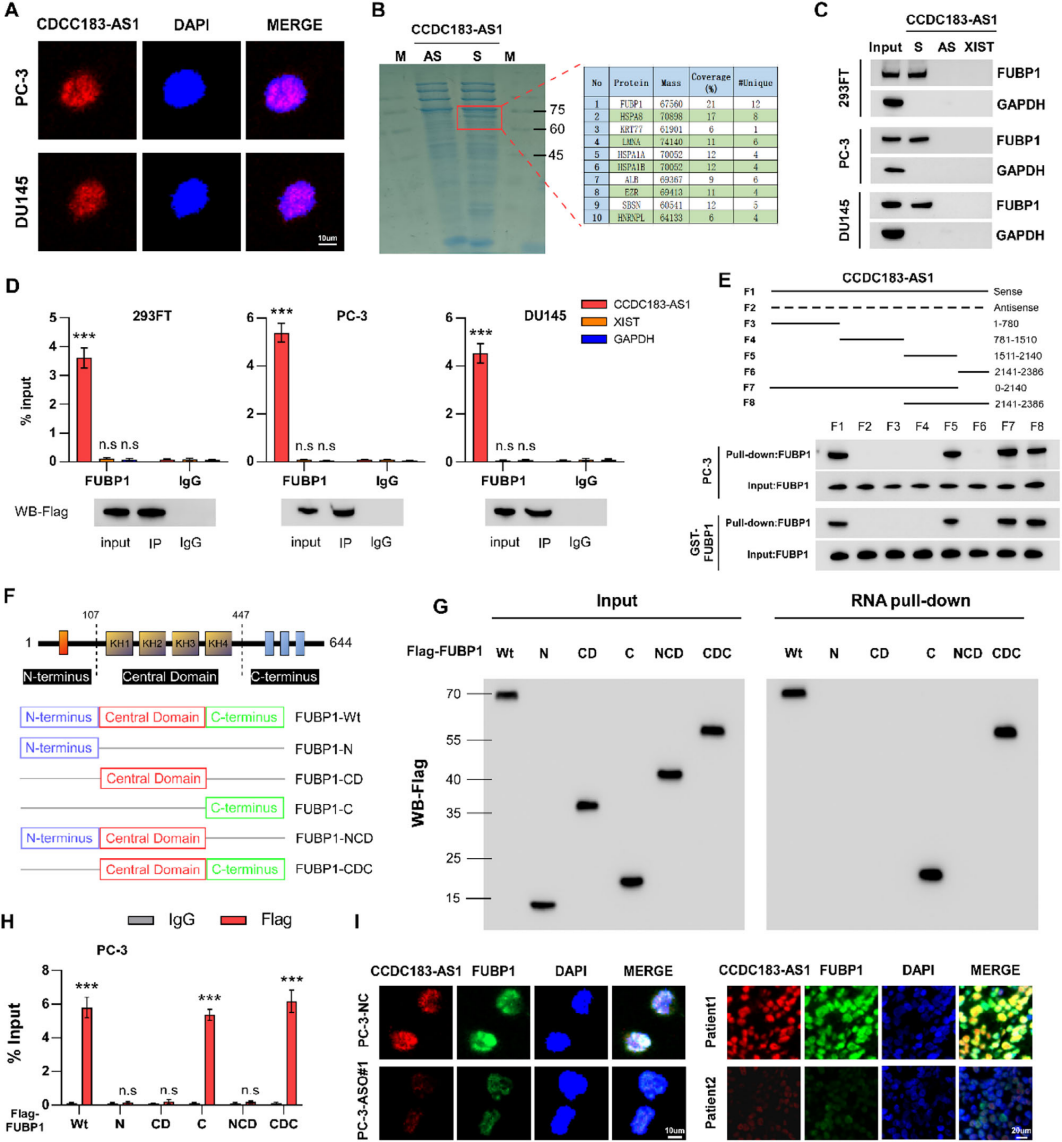
CCDC183‐AS1 interacts with FUBP1 protein. A) RNA FISH analysis showing the subcellular localization of CCDC183‐AS1 in PCa cells. Bars, 10 um. B) Isolation of CCDC183‐AS1 interacting proteins through RNA pull‐down assay in PC‐3 cells, followed by SDS‐PAGE electrophoresis and Coomassie Brilliant Blue staining. C) Confirmation of CCDC183‐AS1 interaction with FUBP1 via RNA pull‐down and Western blotting assay. GAPDH was used as a negative control. D) RIP assay using anti‐FUBP1 and control IgG antibodies, followed by RT‐qPCR assay to detect the enrichment of CCDC183‐AS1, XIST, and GAPDH. E) Design of a series of truncated transcript based on the secondary structure of CCDC183‐AS1 to identify the core region required for interaction with FUBP1 through RNA pull‐down assays. F) Schematic representation of the structure of FUBP1 protein and summary of the FUBP1 variants used in this study. The blue box represents the N‐terminus domain, the red box represents the Central Domain, and the green box represents the C‐terminus domain. G) Identification of the core region necessary for FUBP1 interaction with CCDC183‐AS1 through RNA pull‐down and Western blotting assays in 293FT cells. H) RIP assay using anti‐flag and control IgG antibodies, followed by RT‐qPCR to detect the enrichment of CCDC183‐AS1. I) Representative co‐staining images of CCDC183‐AS1 and FUBP1 in control and CCDC183‐AS1 knockdown PCa cells and in clinical samples from PCa patients using smFISH and immunostaining assays together. Bars, 20 and 10 um. All experiments were performed in biological triplicate. Statistical analyses were performed by Student's t‐test (H) and one‐way ANOVA test (D). *p < 0.05, **p < 0.001, and ***p < 0.0001.

To investigate how CCDC183‐AS1 functions in PCa BM, we employed fluorescence in situ hybridization (FISH) and subcellular fractionation to detect the localization of CCDC183‐AS1 within PCa cells. The findings showed that CCDC183‐AS1 was predominantly found in the nucleus (**Figure** **3**A; Figure S3A, Supporting Information). Notably, we identified several genes adjacent to the CCDC183‐AS1 locus, including CCDC183, RABL6, and NCLP1. Among these, RABL6 and NCLP1 showed slight expression changes based on the TCGA‐PRAD dataset (Figure S3B, Supporting Information). However, silencing CCDC183‐AS1 did not change the expression of these genes, indicating that CCDC183‐AS1 may not be a cis‐acting gene (Figure S3C, Supporting Information). Previous studies have indicated that lncRNAs located in the nucleus exert their biological functions by forming complexes with proteins.^[^ ^27^ ^]^ Therefore, we performed an RNA pull‐down experiment to identify the proteins that interact with CCDC183‐AS1. As shown in Figure 3B, there was a clear band in the pull‐down region of CCDC183‐AS1 with a size between 60 and 75 kDa. Subsequently, mass spectrometry analysis identified some proteins that may interact with CCDC183‐AS1, with FUBP1 ranking first (Figure 3B; Figure S3D, Supporting Information). Western blotting analysis of the pull‐down products revealed that CCDC183‐AS1 bound to FUBP1 (Figure 3C). Meanwhile, RNA immunoprecipitation (RIP) experiments further confirmed the specific interaction between CCDC183‐AS1 and FUBP1 (Figure 3D). To further elucidate how CCDC183‐AS1 interacts with FUBP1, we predicted the potential sites through which CCDC183‐AS1 binds to FUBP1 and the secondary structure of CCDC183‐AS1 using catRAPID and RNAfold websites (http://service.tartaglialab.com/page/catrapid_group; http://rna.tbi.univie.ac.at/cgi‐bin/RNAWebSuite/RNAfold.cgi) (Figure S3E,F, Supporting Information). We constructed several truncated fragments of CCDC183‐AS1 based on the predicted results and performed an RNA pull‐down assay. The results indicated that the F5 fragment of CCDC183‐AS1 (1511‐2140 nt) was the specific binding site for the FUBP1 protein (Figure 3E). Additionally, we created a set of FLAG‐tagged FUBP1 deletion variants to determine which domain of FUBP1 interacts with CCDC183‐AS1 (Figure 3F). Using RNA pull‐down and RIP assays, we discovered that the C‐terminal region (447‐644 aa) of FUBP1 was essential for its interaction with CCDC183‐AS1 (Figure 3G,H; Figure S3G, Supporting Information). Consistently, FISH and IF staining showed that CCDC183‐AS1 and FUBP1 co‐localized in the nuclei of PCa cells and tissues. Interestingly, the expression of FUBP1 in PCa cells with CCDC183‐AS1 knockdown or in tissues with low CCDC183‐AS1 expression was markedly decreased (Figure 3I), indicating that CCDC183‐AS1 may regulate the expression of FUBP1. Hence, these findings imply that the interaction of FUBP1 with CCDC183‐AS1 may be an important factor in the progression of PCa BM.

### 2.4 CCDC183‐AS1 Blocks Ubiquitin‐Mediated Degradation of FUBP1 via Competing with JTV‐1

**Figure 4:**
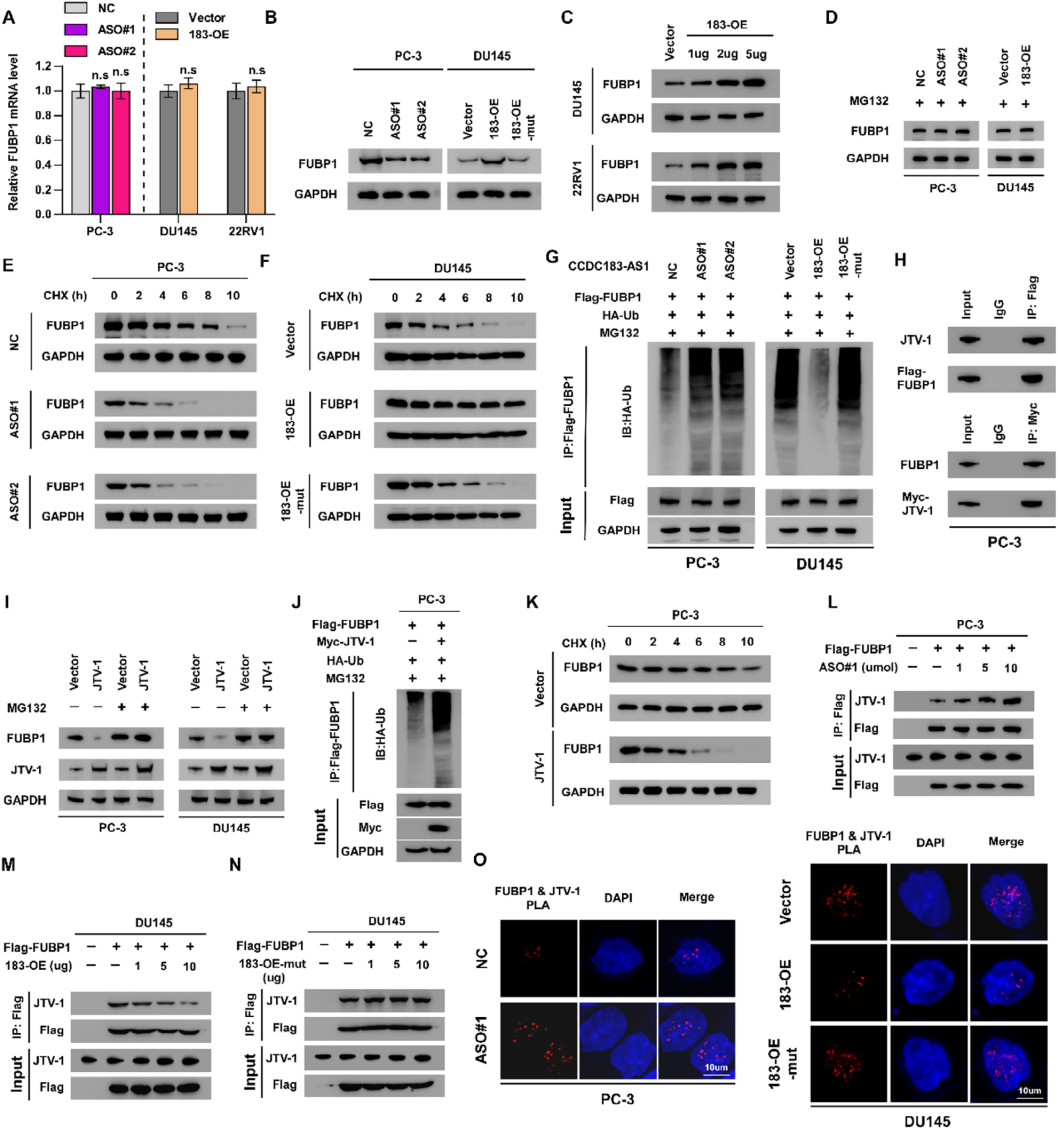
CCDC183‐AS1 blocks ubiquitin‐mediated degradation of FUBP1 via competing with JTV‐1. A) RT‐qPCR assay showing the effect of CCDC183‐AS1 inhibition and overexpression on FUBP1 mRNA levels. B) Western blotting assay showing the effect of CCDC183‐AS1 inhibition and overexpression on FUBP1 protein levels. 183‐OE‐mut, CCDC183‐AS1 mutant. C) Western blotting analysis of FUBP1 expression in PCa cells transfected with various dosages of CCDC183‐AS1 expressing‐ or control‐vector plasmids. D) Western blotting analysis of FUBP1 expression in the indicated cells treated with the proteasome inhibitor MG132 (10 umol L^−1^). E,F) The effect of CCDC183‐AS1 knockdown or overexpressing CCDC183‐AS1 or CCDC183‐AS1‐mut on the half‐life of FUBP1 was evaluated in the indicated cells treated with cyclohexamide (CHX, 50 ug mL^−1^). G) The effect of CCDC183‐AS1 inhibition and overexpression of CCDC183‐AS1 or CCDC183‐AS1‐mut on the ubiquitination level of FUBP1. H) Verification of FUBP1‐JTV‐1 interaction by immunoprecipitation assay. I) Western blotting analysis of FUBP1 expression in the indicated cells treated with the proteasome inhibitor MG132 (10 umol L^−1^). J) The effect of JTV‐1 overexpression on the ubiquitination level of FUBP1. K) The effect of JTV‐1 overexpression on the half‐life of FUBP1 in PCa cells treated with CHX (50 ug mL^−1^). L) The effect of CCDC183‐AS1 inhibition on FUBP1‐JTV‐1 interaction in a dose‐dependent manner. M) The effect of CCDC183‐AS1 overexpression on FUBP1‐JTV‐1 interaction in a dose‐dependent manner. N) The effect of CCDC183‐AS1‐mut overexpression on FUBP1‐JTV‐1 interaction. O) In situ PLA was used to detect the interaction between endogenous FUBP1 and JTV‐1 in the indicated cells. Bars, 10 um. All experiments were performed in biological triplicate. Statistical analyses were performed by one‐way ANOVA test (A). *p < 0.05, **p < 0.001, and ***p < 0.0001.

We investigated how the interaction between CCDC183‐AS1 and FUBP1 affects FUBP1 expression by conducting RT‐qPCR and Western blotting to measure FUBP1 mRNA and protein levels following ASO treatment or CCDC183‐AS1 overexpression. As shown in **Figures** **4**A,B and S4A (Supporting Information), ASO treatment and CCDC183‐AS1 overexpression significantly altered the protein level of FUBP1, whereas the mRNA levels did not show significant changes. Notably, overexpression of the CCDC183‐AS1 mutant plasmid (deletion of 1511–2140 nt) did not alter the protein level of FUBP1 (Figures 4B and S4A,B, Supporting Information), indicating that FUBP1 protein was regulated by CCDC183‐AS1 depending on their interaction. Meanwhile, elevated levels of CCDC183‐AS1 led to a dose‐dependent rise in FUBP1 protein expression (Figures 4C and S4C, Supporting Information). Furthermore, we tested whether CCDC183‐AS1 affects FUBP1 degradation using the proteasome inhibitor MG132. As shown in Figure 4D and Figure S4D,E (Supporting Information), when MG132 was used to inhibit proteasome‐mediated protein degradation, inhibiting or overexpressing CCDC183‐AS1 did not affect the level of FUBP1 protein. Silencing of CCDC183‐AS1 significantly accelerated FUBP1 protein degradation (Figures 4E and S4F, Supporting Information), while overexpressing CCDC183‐AS1, but not the CCDC183‐AS1 mutant, prolonged the half‐life of the FUBP1 protein (Figures 4F and S4G, Supporting Information). Additionally, inhibition of CCDC183‐AS1 markedly increased the ubiquitination of FUBP1, while overexpression of CCDC183‐AS1 greatly eliminated the ubiquitination of FUBP1, which was not regulated by the CCDC183‐AS1 mutant (Figures 4G and S4H, Supporting Information). These results demonstrated that CCDC183‐AS1 blocked the degradation of FUBP1 in a ubiquitination‐dependent manner.

Previous research has demonstrated that JTV‐1 is capable of physically interacting with the C‐terminal region of FUBP1, thereby facilitating the degradation of FUBP1 via ubiquitination.^[^ ^22^ ^]^ Based on our results, we hypothesized that CCDC183‐AS1 competes with JTV‐1 for binding to the same domain of FUBP1, consequently inhibiting the ubiquitination‐induced proteasomal degradation of FUBP1. To validate this hypothesis, IP analysis was conducted, which confirmed the interaction of FUBP1 with JTV‐1 (Figure 4H). Subsequently, we observed that JTV‐1 overexpression dramatically decreased FUBP1 expression, and inhibition of proteasome function reversed the effect of JTV‐1 on FUBP1 protein levels (Figure 4I; Figure S4I,J, Supporting Information). Meanwhile, overexpression of JTV‐1 enhanced the ubiquitination of FUBP1 and accelerated its degradation (Figure 4J,K; Figure S4K,L, Supporting Information). Our study revealed that inhibition of CCDC183‐AS1 increased, whereas overexpression of CCDC183‐AS1 weakened the interaction of JTV‐1 with FUBP1 in a dose‐dependent fashion (Figure 4L,M; Figure S4M,N, Supporting Information). In contrast, the CCDC183‐AS1 mutant had no influence on the interaction of JTV‐1 with FUBP1 (Figure 4N; Figure S4O, Supporting Information). Notably, the proximity ligation assay (PLA) also confirmed the interaction of JTV‐1 with FUBP1, which was enhanced by CCDC183‐AS1 knockdown but inhibited by CCDC183‐AS1 overexpression, instead of the CCDC183‐AS1 mutant (Figure 4O). Together, these findings suggest that CCDC183‐AS1 competitively binds to FUBP1 with JTV‐1, thereby inhibiting JTV‐1‐mediated ubiquitination and degradation of FUBP1.

### 2.5 CCDC183‐AS1 Induced LIGHT Promoting Osteoclastogenesis in a FUBP1‐Dependent Manner

**Figure 5:**
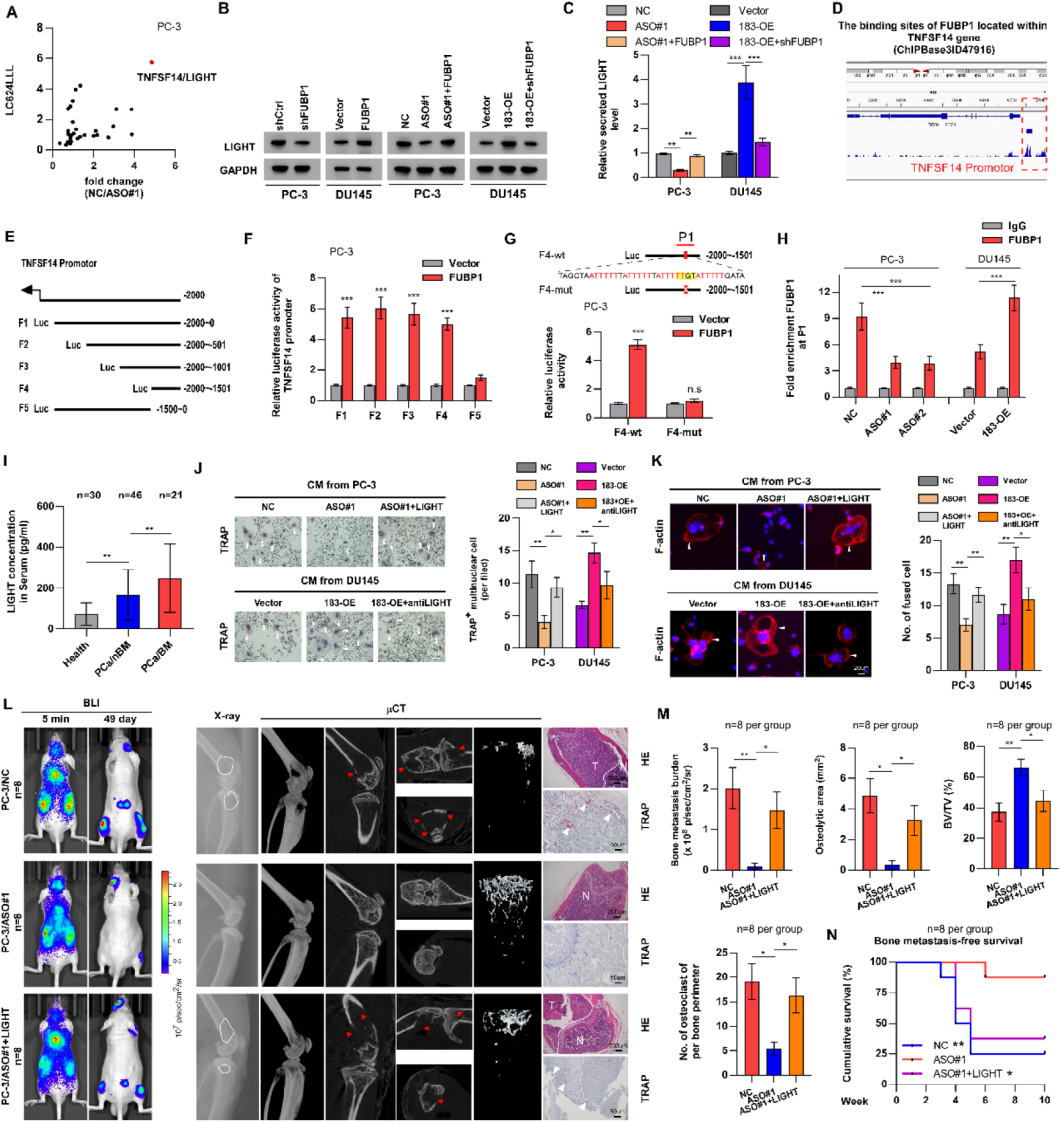
CCDC183‐AS1 induced LIGHT promoting osteoclastogenesis in an FUBP1‐dependent manner. A) RT‐qPCR analysis of mRNA levels of 32 common osteoclastogenic factors following the inhibition of CCDC183‐AS1 and FUBP1 expression. B) Western blotting analysis of LIGHT protein level in the indicated cells. C) ELISA analysis of LIGHT level in the medium of the indicated cells. D) The ChIP‐seq results based on public database (ChIPBase) show a significant overlapping peak of FUBP1 in the promoter region of TNFSF14. E) Schematic illustration of the TNFSF14 promoter region and a summary of the TNFSF14 promoter variants used in this study. F) Measurement of TNFSF14 promoter activity using a dual‐luciferase reporter assay after transfection with different TNFSF14 promoter variants in FUBP1‐overexpressing and control cell lines. G) Luciferase reporter assay showing the luciferase activity of F4‐wt and F4‐mut (P1 deletion) reporter in the indicated cells. H) ChIP assay demonstrating the enrichment of FUBP1 on P1 fragments of TNFSF14 promoter in the indicated groups. I) Detection of LIGHT level in the serum of health individuals and PCa patients with or without BM using ELISA. J) Osteoclast differentiation assay using TRAP staining after treatment with CM from the indicated PCa cells (left). Quantification of TRAP^+^ multinuclear OCs in the osteoclast differentiation assays. Bars, 50 um. K) Formation of actin rings (left) and quantification of fused multinucleated cells in the indicated RAW264.7 cells. Bars, 20 um. L) Representative images of BLI, X‐ray, µCT, HE and TRAP staining of BM lesions. M) Quantification of bone metastasis burden, osteolytic areas, BV/TV and OCs. N) Kaplan‐Meier analysis for bone metastasis‐free survival. Bars, 200 and 50 um. All experiments were performed in biological triplicate. Statistical analyses were performed by Student's t‐test (A, F, G, H) and one‐way ANOVA test (C, I, J, K, M), and the log‐rank test (N). *p < 0.05, **p < 0.001, and ***p < 0.0001.

To further delve into the process of osteoclastogenesis induced by CCDC183‐AS1, we examined the potential downstream factors involved in CCDC183‐AS1‐induced osteolytic BM. Among the 32 commonly observed bone‐remodeling factors,^[^ ^28^, ^29^, ^30^ ^]^ the mRNA levels of TNFSF14, which encodes the LIGHT protein, in PCa cells subjected to CCDC183‐AS1 inhibition or FUBP1 knockdown showed the most substantial decrease compared to those in control cells (**Figure** **5**A; Figure S5A and Table S1, Supporting Information). Therefore, TNFSF14 was selected for further investigation. The protein levels of LIGHT were regulated by CCDC183‐AS1 and FUBP1, and FUBP1 reversed the effect of CCDC183‐AS1 on TNFSF14 at both the mRNA and protein levels (Figure 5B,C; Figure S5B–D, Supporting Information). Previous studies have shown that FUBP1 serves as a transcriptional regulator that governs the transcription of specific target genes. Interestingly, our analysis revealed a notable peak in FUBP1 enrichment within the promoter region of TNFSF14, as indicated by ChIP‐seq data obtained from the ChIPBase database (Figure 5D). This suggests that FUBP1 may modulate the expression of TNFSF14 by controlling its transcription. To verify the transcriptional regulation of TNFSF14 by FUBP1, we generated various truncations of the TNFSF14 promoter and assessed luciferase activity (Figure 5E). FUBP1 prominently increased the luciferase activity of the TNFSF14 promoter, and the F4 fragment (‐2000 to‐1501 nt) was essential for FUBP1 in regulating TNFSF14 transcription (Figure 5F). Additionally, a putative FUSE‐like sequence (5′‐ATTTTT‐3′, P1)^[^ ^22^ ^]^ was discovered within the F4 fragment, which was hypothesized to mediate the binding of FUBP1 to DNA (Figure 5G). To investigate the regulatory role of FUBP1 in TNFSF14 expression via the P1 sequence, a mutant F4 fragment with the P1 deletion was generated, followed by a luciferase reporter assay. The results suggested that removal of the P1 sequence abrogated the transcriptional activation of TNFSF14 induced by FUBP1 (Figure 5G). Furthermore, our study confirmed that alterations in CCDC183‐AS1 expression significantly influenced the enrichment of FUBP1 in the TNFSF14 promoter, as demonstrated by ChIP‐qPCR assay (Figure 5H). In summary, these results demonstrate that FUBP1 plays a role in the transcriptional upregulation of TNFSF14 in PCa.

Next, an ELISA assay was conducted to quantify the levels of LIGHT in the serum of patients with PCa. Our findings demonstrated that LIGHT protein levels were significantly elevated in patients with BM, compared with those without BM and healthy controls (Figure 5I). Additionally, survival analysis based on the TCGA database demonstrated that TNFSF14 expression levels were negatively associated with the prognosis of PCa patients (Figure S5D, Supporting Information). These results illustrated that CCDC183‐AS1‐induced LIGHT may be involved in PCa BM. In accordance with this hypothesis, our study demonstrated that LIGHT upregulation counteracted the inhibitory effect of CCDC183‐AS1 depletion on osteoclast differentiation and activation (Figure 5J,K). Conversely, LIGHT inhibition negated the stimulatory effect of CCDC183‐AS1 upregulation on osteoclast differentiation and activation (Figure 5J,K). Concurrently, in vivo experiments validated that mice in the ASO with LIGHT overexpression group showed accelerated bone metastasis onset, heightened bone metastasis burden, increased osteolytic area, elevated number of TRAP^+^ OCs, and poor bone metastasis‐free survival compared to mice treated solely with ASO (Figure 5L–N; Figure S5E, Supporting Information). These results show that CCDC183‐AS1 induces LIGHT expression in a FUBP1‐dependent manner, promoting osteoclastogenesis and BM.

### 2.6 CCDC183‐AS1 Induces EMT of PCa Cells through the FUBP1/C‐MYC Axis

CCDC183‐AS1 has previously been reported to promote the migratory and invasive capabilities of tumor cells.^[^ ^31^, ^32^ ^]^ Meanwhile, C‐MYC, as a classical downstream target of FUBP1, is involved in various cellular processes including tumor cell proliferation, migration, invasion, differentiation, and epithelial‐mesenchymal transition (EMT).^[^ ^21^, ^33^, ^34^, ^35^ ^]^ Therefore, we hypothesized that CCDC183‐AS1 may regulate EMT and the migratory and invasive abilities of PCa cells through modulation of C‐MYC expression. Interestingly, correlation analysis based on the TCGA‐PRAD database revealed a positive correlation between CCDC183‐AS1 and C‐MYC levels (Figure S6A, Supporting Information). Further RT‐qPCR and western blot assays indicated that CCDC183‐AS1 or FUBP1 knockdown significantly decreased C‐MYC expression and FUBP1 overexpression could reverse the effect of CCDC183‐AS1 knockdown on C‐MYC (Figure S6B,C, Supporting Information). To investigate whether CCDC183‐AS1 affects EMT of PCa cells, we found CCDC183‐AS1 knockdown increased E‐cadherin expression, while decreased vimentin expression, and C‐MYC knockdown had the same effect (Figure S6D, Supporting Information). Transwell assays indicated that CCDC183‐AS1 knockdown dramatically inhibited the migration and invasion of PCa cells, while C‐MYC overexpression attenuated this effect (Figure S6E,F, Supporting Information). In contrast, CCDC183‐AS1 overexpression promoted the migration and invasion of PCa cells, whereas C‐MYC knockdown abolished the promoting function of CCDC183‐AS1 (Figure S6E,F, Supporting Information). Furthermore, we explored whether C‐MYC mediates the regulatory effect of CCDC183‐AS1 on bone metastasis in vivo. Our results demonstrated that overexpression of C‐MYC partially reversed the inhibitory effect of CCDC183‐AS1 knockdown on bone metastasis (Figure S6G–J, Supporting Information). The above results indicate that CCDC183‐AS1 induces EMT of PCa cells to enhance bone metastasis through the FUBP1/C‐MYC axis.

### 2.7 Elevated CCDC183‐AS1 Expression is Linked to Hypomethylation of Its Own Promoter

**Figure 6:**
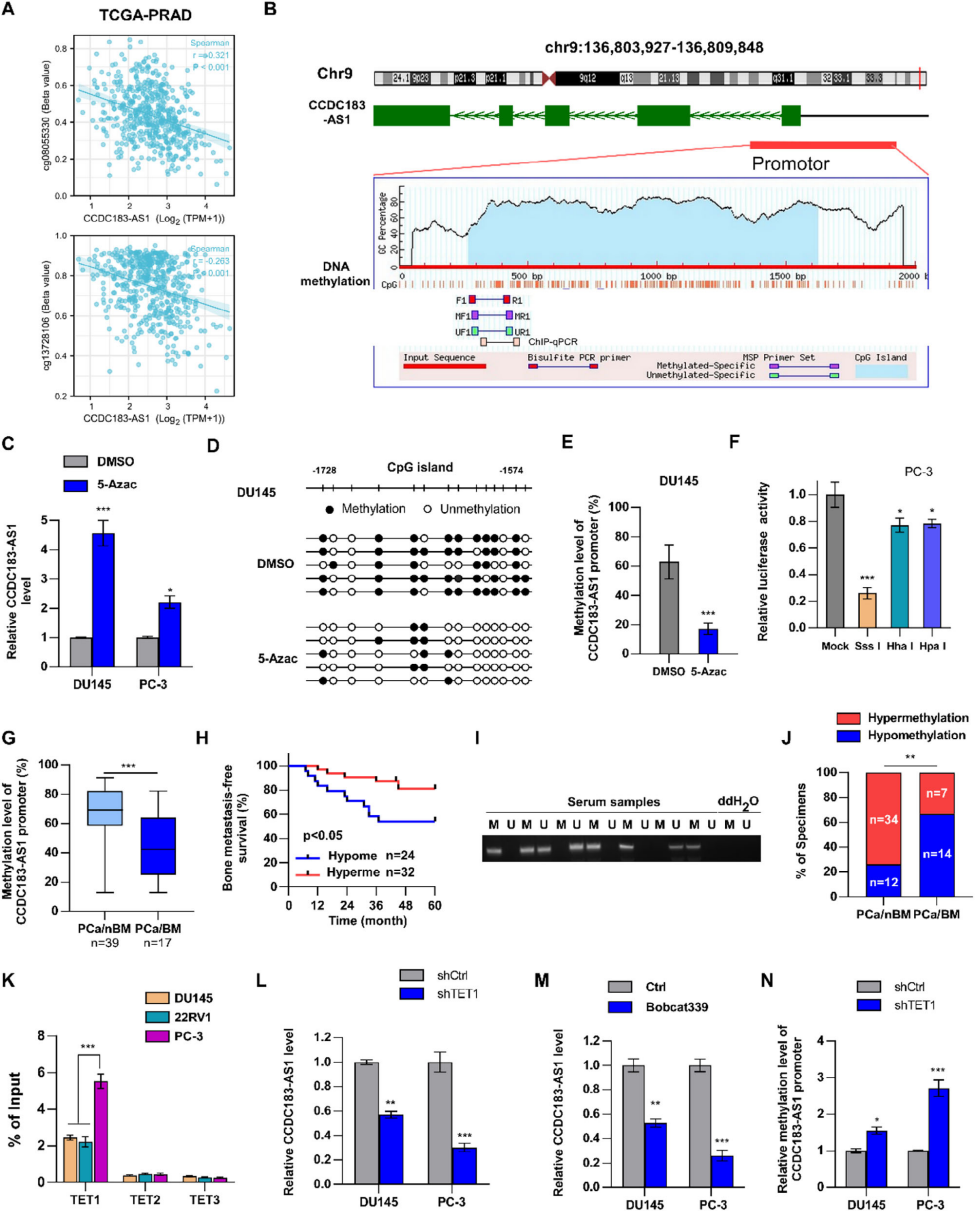
Elevated CCDC183‐AS1 is associated with its promoter hypomethylation. A) Correlation analysis of CCDC183‐AS1 expression and methylation level of CCDC183‐AS1 promoter in the TCGA‐PRAD cohort. B) The CpG Island of the CCDC183‐AS1 promoter region predicted by MethPrimer website. C) RT‐qPCR showing the effect of 5‐Azac (5 µm) treatment for 48 h on CCDC183‐AS1 expression in PCa cells. D,E) BSP analysis (D) and quantification (E) of the methylation status of CCDC183‐AS1 promoter in the indicated cells. F) Methylated or mock‐methylated CCDC183‐AS1 promoter–luciferase reporter constructs were transiently transfected into PC‐3 cells. Luciferase activity was analyzed after a 48‐hour transfection. G) BSP assay showing the comparison of methylation level of CCDC183‐AS1 promoter in PCa/nBM and PCa/BM tissues. H) Kaplan‐Meier analysis of bone metastasis‐free survival in patients with low and high methylated CCDC183‐AS1 promoter in PCa. The high and low methylation groups are bounded by 50% of methylation value. Hypome, hypomethylation; Hyperme, hypermethylation. I) Representative images of MSP analysis for CCDC183‐AS1 promoter region in serum DNA from PCa patient. M represents methylation and U represents unmethylation. J) MSP assay showing the comparison of the methylation status of CCDC183‐AS1 in serum DNA from PCa/nBM and PCa/BM. Hypermethylation refers to the condition where the intensity of M is greater than that of U. K) ChIP analysis to determine the enrichment of TET enzymes on the CCDC183‐AS1 promoter. L) RT‐qPCR to assess the effect of TET1 knockdown on CCDC183‐AS1 expression in PCa cells. M) RT‐qPCR to assess the effect of the TET1 enzyme inhibitor (Bobcat339, 10 µm, 24 h) on CCDC183‐AS1 expression in PCa cells. N) MS‐qPCR assay to assess the effect of TET1 knockdown on the methylation level of CCDC183‐AS1 promoter in PCa cells. All experiments were performed in biological triplicate. Statistical analyses were performed by Student's t‐test (C, E, G, L, M, N), Spearman correlation test (A), and one‐way ANOVA test (F, K). *p < 0.05, **p < 0.001, and ***p < 0.0001.

Recent studies suggest that epigenetic processes like DNA methylation significantly influence RNA expression.^[^ ^36^, ^37^ ^]^ In our study, correlation analysis utilizing TCGA‐PRAD data revealed a negative association between CCDC183‐AS1 expression and the methylation status of multiple CpG sites within the promoter region of CCDC183‐AS1 (**Figure** **6**A; Figure S7A, Supporting Information). Notably, we also observed the presence of a CpG island within the promoter region of CCDC183‐AS1 through an analysis conducted by the MethPrimer website (Figure 6B). To validate the role of DNA methylation in the upregulation of CCDC183‐AS1, PCa cells were treated with 5‐Azac, a potent DNA‐demethylating agent. Subsequent analysis revealed a significant upregulation in the expression of CCDC183‐AS1 after 5‐Azac treatment (Figure 6C). Furthermore, bisulfite sequencing PCR (BSP) revealed that the methylation level of the CCDC183‐AS1 promoter markedly decreased after 5‐Azac treatment (Figure 6D,E; Figure S7B, Supporting Information). Conversely, the administration of S‐adenosyl‐l‐methionine (AdoMet), a compound known to suppress demethylase function, to PCa cells led to decreased expression of CCDC183‐AS1 (Figure S7C, Supporting Information). These results indicated that DNA methylation influences the transcription of CCDC183‐AS1. To investigate whether DNA methylation could directly regulate the activity of the CCDC183‐AS1 promoter, we cloned the CCDC183‐AS1 promoter region (‐1726 to ‐1547) into a luciferase reporter construct. The cloned fragments were methylated in vitro using Sssl (14 methylated CpGs), Hpall (3 methylated CpGs), and Hhal methylases (3 methylated CpGs). After transfecting PCa cells with methylated or mock‐methylated luciferase reporter constructs, the activity of the CCDC183‐AS1 promoter was measured. As shown in Figure 6F, DNA methylation inhibited CCDC183‐AS1 promoter activity in a dose‐dependent manner. Subsequently, we assessed the methylation level of the CCDC183‐AS1 promoter in PCa tissues. Our findings revealed that the methylation level of the CCDC183‐AS1 promoter was dramatically lower in PCa patients with BM than in those without BM (Figure 6G). Furthermore, hypomethylation of the CCDC183‐AS1 promoter was associated with poor bone metastasis‐free survival outcomes in patients with PCa (Figure 6H). Emerging evidence suggests that blood‐based biomarkers are more readily obtainable and less invasive than those obtained from tumor tissues. Additionally, advancements in medical technology enable the identification of epigenetic alterations in cell‐free tumor DNA present in the serum of individuals with cancer.^[^ ^38^, ^39^ ^]^ To validate the potential of CCDC183‐AS1 hypomethylation in serum DNA as a biomarker of PCa BM, methylation‐specific PCR (MSP) was conducted to assess the methylation status of the CCDC183‐AS1 promoter in serum samples from patients with PCa. We found that, in PCa patients with BM, the proportion of patients with hypomethylation of the CCDC183‐AS1 promoter was higher than that in PCa patients without BM (Figure 6I,J). Collectively, these findings indicate that upregulation of CCDC183‐AS1 is associated with hypomethylation of its promoter, suggesting that hypomethylation of the CCDC183‐AS1 promoter could be a potential prognostic indicator for tracking PCa BM.

### 2.8 KDM5C Recruited TET1 to the Promoter Region of CCDC183‐AS1, Promoting DNA Demethylation of CCDC183‐AS1

To determine the underlying mechanism of hypomethylation of the promoter region of CCDC183‐AS1, we initially analyzed the levels of 5‐methylcytosine (5 mc) enrichment in PCa cells. We observed significantly lower 5 mc enrichment in PC‐3 cells than in DU145 and 22RV1 cells (Figure S7D, Supporting Information). Previous studies have demonstrated that DNMT1, DNMT3A, and DNMT3B function as canonical DNA methyltransferases that are responsible for the establishment and preservation of DNA methylation. Conversely, the TET protein family, comprising TET1, TET2, and TET3, catalyzes the conversion of 5 mc to 5‐hydroxymethylcytosine (5hmc), thereby initiating DNA demethylation.^[^ ^40^ ^]^ Therefore, we performed ChIP assays to assess the binding of these six proteins to the CCDC183‐AS1 promoter region. ChIP results indicated a significant increase in DNMT1 and DNMT3B enrichment in the CCDC183‐AS1 promoter region. However, no variation of their enrichment was observed among the different PCa cells with varying 5mc status of the CCDC183‐AS1 promoter, suggesting that these enzymes may not be responsible for the discrepancies in DNA methylation of the CCDC183‐AS1 promoter (Figure S7E, Supporting Information). Subsequently, our findings revealed a notable increase in TET1 enrichment in the CCDC183‐AS1 promoter region, with discrepancies observed across various PCa cell lines, suggesting that TET1‐mediated demethylation may play a role in hypomethylation of the CCDC183‐AS1 promoter region (Figure 6K). TET1 knockdown significantly decreased the expression of CCDC183‐AS1 (Figure 6L; Figure S7F, Supporting Information). The TET1 enzyme inhibitor (Bobcat339) could markedly reduce CCDC183‐AS1 expression in PCa cells, indicating that the regulation of CCDC183‐AS1 by TET1 depends on its enzyme activity (Figure 6M). In addition, MS‐qPCR confirmed that TET1 knockdown dramatically increased the methylation of the CCDC183‐AS1 promoter region (Figure 6N). These results suggest that TET1 mediates demethylation of the CCDC183‐AS1 promoter region.

**Figure 7:**
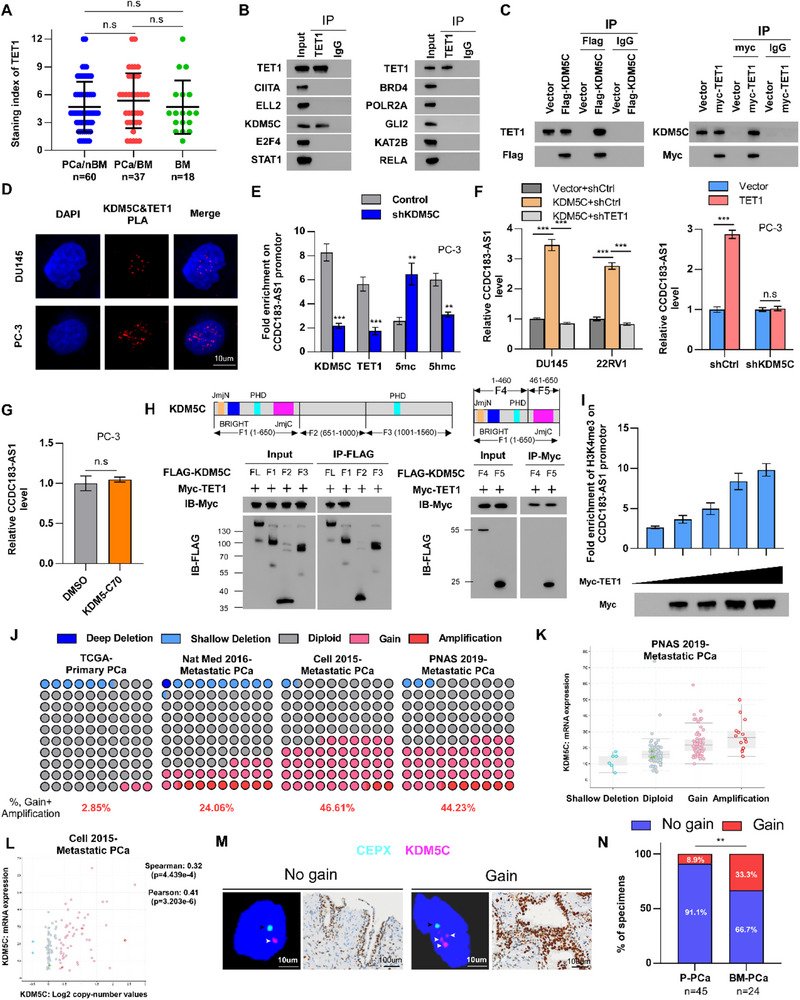
KDM5C recruited TET1 to the promoter region of CCDC183‐AS1, promoting demethylation of CCDC183‐AS1. A) IHC assay showing the expression of TET1 in PCa/nBM (n = 60), PCa/BM (n = 37), and BM (n = 18) tissues. B) IP assay showing the interaction of TET1 with the top 10 transcriptional regulators in the promoter region of CCDC183‐AS1 in PC‐3 cells. C) IP and western blotting assays confirming the interaction between TET1 and KDM5C in 293FT cells. D) Proximity ligation assay (PLA) detecting the interaction between TET1 and KDM5C, with red spots indicating the interaction. Bars, 10um. E) ChIP analysis showing the effect of KDM5C knockdown on the enrichment levels of KDM5C, TET1, 5mc, and 5hmc in the promoter region of CCDC183‐AS1. F) RT‐qPCR analysis for detecting CCDC183‐AS1 level in the indicated DU145 and 22RV1 (left) and PC‐3(right) cells. G) RT‐qPCR analysis of the effect of the KDM5C enzyme inhibitor (KDM5‐C70) on CCDC183‐AS1 expression in PC‐3 cells. H) 293FT cells transfected with Myc‐TET1 and Flag‐labeled, full length (FL) or truncated forms of KDM5C were subjected to IP with anti‐Flag antibody followed by western blotting analysis as indicated (left). 293FT cells transfected with Flag‐labeled, full length (FL) or truncated forms of KDM5C and Myc‐TET1 were subjected to IP with anti‐Myc antibody followed by western blotting analysis as indicated (right). I) TET1 significantly reduces KDM5C histone demethylase activity in a dose‐dependent manner. J) Copy number gain of KDM5C in primary PCa and metastatic PCa from published database deposited in cBioPortal (Nat Med. 2016 Mar;22(3):298‐305; Cell. 2015 May 21;161(5):1215‐1228; PNAS. 2019 Jun 4;116(23):11428‐11436.). K) KDM5C expression in patients with different copy number (PNAS. 2019 Jun 4;116(23):11428‐11436). L) Correlation between KDM5C copy number value and mRNA expression in the metastatic PCa cohort (Cell. 2015 May 21;161(5):1215‐1228). M) Representative images of FISH staining for KDM5C genomic gain status and corresponding IHC staining for KDM5C protein in primary tumors from PCa patients with or without BM. The black arrow indicates the X chromosome; The white arrow indicates the KDM5C gene. Bars, 100 and 10 um. N) Percentages of KDM5C copy number gain in PCa patients with different BM statuses. All experiments were performed in biological triplicate. Statistical analyses were performed by Student's t‐test (E, F‐right, G) and one‐way ANOVA test (A, F‐left, I, K), Spearman correlation test (L) and χ2 test (N). *p < 0.05, **p < 0.001, and ***p < 0.0001.

Based on previous research indicating the dysregulation of TET1 in PCa,^[^ ^41^ ^]^ we analyzed its expression within this particular context. However, the IHC results from our PCa cohort showed no statistically significant variation in TET1 protein expression among PCa/nBM, PCa/BM, and BM tissues (**Figure** **7**A). Similarly, a differential analysis based on GSE21032 dataset showed no significant difference in TET1 expression between primary and metastatic PCa tissues (Figure S8A, Supporting Information). However, we found that the enrichment of TET1 in the CCDC183‐AS1 promoter region was dramatically increased in BM and PCa/BM tissues relative to PCa/nBM (Figure S8B, Supporting Information). Meanwhile, a positive correlation was observed between TET1 enrichment within the CCDC183‐AS1 promoter region and CCDC183‐AS1 level in PCa cell lines (Figure S8C, Supporting Information). These findings indicated that the expression differences of CCDC183‐AS1 may depend on the enrichment of TET1 in the CCDC183‐AS1 promoter rather than the expression differences of TET1 in PCa. Hence, it is postulated that other factors may have contributed to the discrepancies noted in TET1 enrichment in the CCDC183‐AS1 promoter. Previous studies have reported that transcriptional regulatory factors regulate gene expression by recruiting epigenetic factors.^[^ ^42^, ^43^ ^]^ Hence, we hypothesized that certain transcriptional regulatory factors might be involved in mediating variations in TET1 enrichment in the CCDC183‐AS1 promoter. The top 10 transcriptional regulatory factors bound to the CCDC183‐AS1 promoter region were identified by analyzing the Cistrome Data Browser website (http://cistrome.org/db/#/) (Figure S8D, Supporting Information). The levels of CIITA, KDM5C, BRD4, POLR2A, and RELA were significantly and positively correlated with CCDC183‐AS1 in the TCGA‐PRAD dataset (Figure S8E, Supporting Information), suggesting a potential regulatory relationship between these five factors and CCDC183‐AS1. The IP findings validated that TET1 specifically interacted with KDM5C among the top 10 transcriptional regulators (Figure 7B). Similarly, exogenous IP and PLA further confirmed the interaction between TET1 and KDM5C (Figure 7C,D). To investigate whether KDM5C recruits TET1 to the CCDC183‐AS1 promoter, a ChIP assay was performed. Our findings demonstrated that KDM5C could interact with the promoter of CCDC183‐AS1. Following KDM5C knockdown, a decrease in the enrichment of KDM5C, TET1, and 5‐hydroxymethylcytosine (5 hmC) was observed in the CCDC183‐AS1 promoter region. Conversely, an increased enrichment of 5‐methylcytosine (5 mC) was detected in the CCDC183‐AS1 promoter region (Figure 7E). These results indicate that KDM5C facilitates TET1 binding to the CCDC183‐AS1 promoter and plays a role in regulating DNA demethylation, particularly the conversion of 5 mC to 5 hmC. Meanwhile, silencing of KDM5C significantly reduced the expression level of CCDC183‐AS1 (Figure S8F, Supporting Information). Conversely, ectopic KDM5C prominently increased CCDC183‐AS1 level, whereas the induced ability by KDM5C was reversed when TET1 was silenced (Figure 7F). Compared to control cells, KDM5C knockdown significantly attenuated the ability of TET1 to enhance CCDC183‐AS1 expression (Figure 7F). The above findings demonstrated that KDM5C mediates CCDC183‐AS1 upregulation via the demethylation capacity of TET1 which depends on the recruitment by KDM5C to CCDC183‐AS1 promoter. However, it is widely recognized that KDM5C, functioning as a histone demethylase, primarily suppresses gene expression by targeting H3K4me2/3 through its demethylase activity.^[^ ^44^ ^]^ Hence, we conducted additional experiments to determine the potential influence of KDM5C on the transcription of CCDC183‐AS1 through its effect on histone demethylation. Intriguingly, our findings revealed that KDM5C enzyme inhibitor (KDM5‐C70) had no significant impact on the expression of CCDC183‐AS1 (Figure 7G). Additionally, transfection with the wild‐type or catalytic mutant KDM5C (H514A) plasmids upregulated CCDC183‐AS1 expression (Figure S8G, Supporting Information). These findings indicated that KDM5C modulated CCDC183‐AS1 expression independent of its demethylase function. Subsequently, we aimed to elucidate the process by which KDM5C experiences a loss of enzymatic function upon the activation of CCDC183‐AS1. Previous research has indicated that the demethylase activity of KDM5C is inhibited when its JmjC domain binds to other proteins.^[^ ^45^ ^]^ Therefore, we investigated whether TET1 binds to the enzymatic domain of KDM5C. We generated full‐length and truncated mutant forms of KDM5C, and conducted an IP assay, which revealed that the F1 truncated fragment of KDM5C exhibited interaction with TET1 (Figure 7H). Next, we divided the F1 fragment into the F4 and F5 fragments and found that TET1 mainly bound to the catalytic domain, JmjC of KDM5C (Figure 7H). Based on these findings, we proposed that TET1 may mask the demethylase activity of KDM5C through this interaction. To verify this hypothesis, ChIP experiments were performed and revealed that TET1 significantly decreased the demethylase activity of KDM5C in a dose‐dependent manner (Figure 7I; Figure S8H, Supporting Information). Collectively, these results show that KDM5C recruits TET1 to the promoter of CCDC183‐AS1, which promotes DNA demethylation in the CCDC183‐AS1 promoter region, and TET1 masks the demethylase activity of KDM5C, leading to the transcriptional activation of CCDC183‐AS1.

Furthermore, our analysis based on public databases revealed that KDM5C expression levels were significantly higher in metastatic PCa tissues compared to primary PCa tissues (Figure S8I, Supporting Information). The mechanism underlying KDM5C upregulation was further investigated in PCa BM. Analysis based on the cBioPortal website (https://www.cbioportal.org/) revealed a higher frequency of KDM5C copy number gain or amplification in metastatic PCa compared with primary PCa (Figure 7J; Figure S8J, Supporting Information). Meanwhile, the copy number of KDM5C was positively correlated with its mRNA expression, indicating that gene locus gain may be the underlying mechanism for KDM5C upregulation in PCa BM (Figure 7K,L; Figure S7K,L, Supporting Information). In our cohort, DNA FISH and immunohistochemistry (IHC) assays consistently indicated that patients with KDM5C copy number gain showed a marked rise in KDM5C expression levels (Figure 7M; Figure S7M, Supporting Information). In addition, copy number gain of KDM5C was observed in 10.2% of primary PCa tissues and 32.5% of BM‐PCa tissues (Figure 7N), indicating that the copy number of KDM5C may serve as a predictive biomarker for PCa BM. Furthermore, survival analysis indicated that elevated levels of KDM5C were linked to a worse prognosis for patients with PCa (Figure S7N, Supporting Information). In summary, our findings indicate that copy number gain‐induced KDM5C epigenetically activates CCDC183‐AS1 transcription by recruiting TET1 in PCa cells.

### 2.9 FUBP1‐IN‐1 Inhibits CCDC183‐AS1‐Induced Osteoclastogenesis In Vitro and PCa BM In Vivo

**Figure 8:**
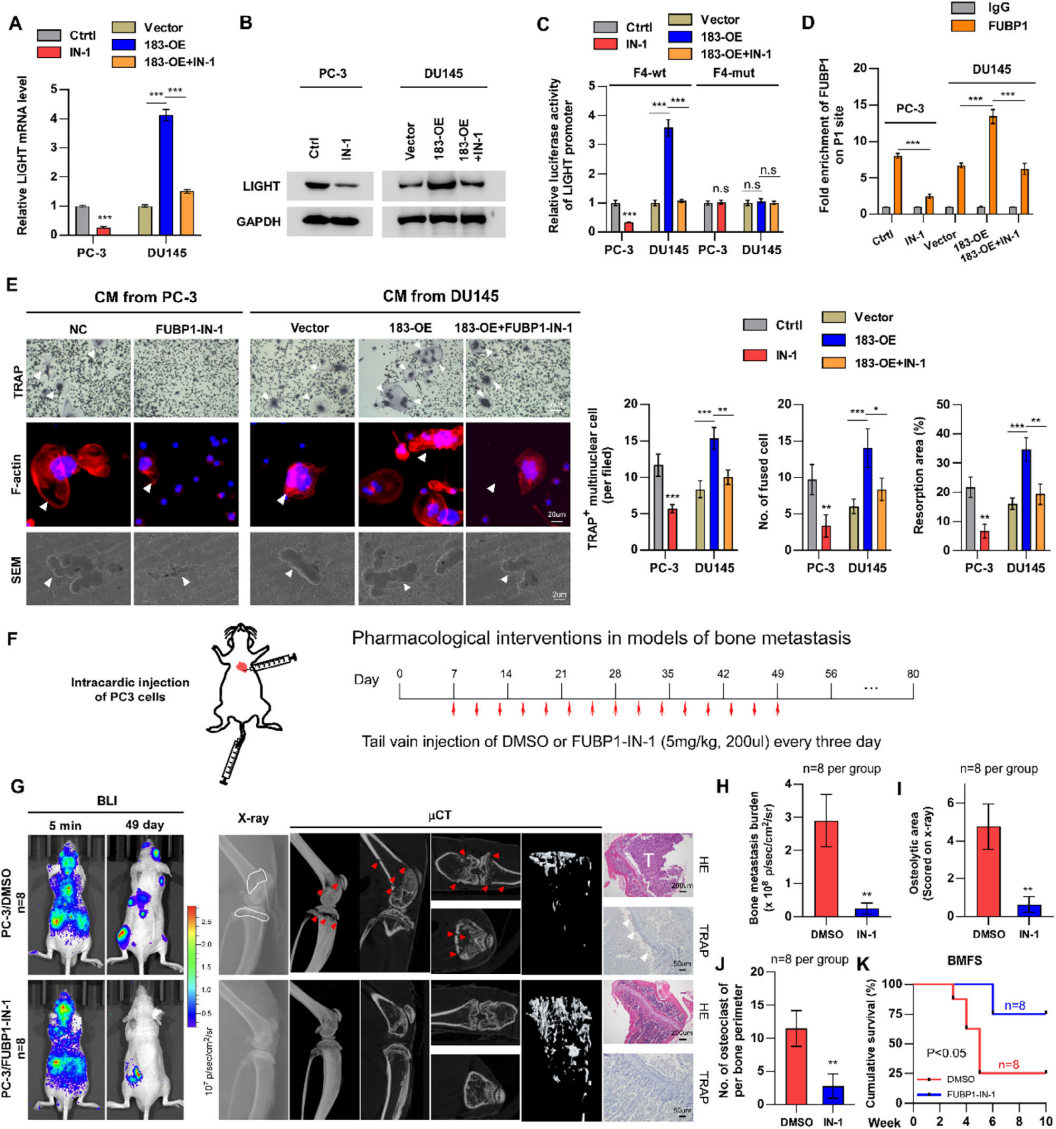
FUBP1‐IN‐1 inhibits CCDC183‐AS1‐induced osteoclastogenesis in vitro and PCa BM in vivo. A) RT‐qPCR analysis of the effect of FUBP1‐IN‐1 (IN‐1) on TNFSF14 mRNA (20 um, 24 h). B) Western blotting analysis of the effect of FUBP1‐IN‐1 (IN‐1) on LIGHT protein (20 um, 24 h). C) Dual‐luciferase reporter assay to measure the effect of FUBP1‐IN‐1 on LIGHT promoter activity (20 um, 24 h). D) ChIP analysis to determine the effect of FUBP1‐IN‐1 on the enrichment of FUBP1 on TNFSF14 promoter (P1 site) (20 um, 24 h). E) TRAP assay, F‐actin staining and SEM showing the effect of FUBP1‐IN‐1 on osteoclast differentiation and activation in vitro. Bars, 50, 20, and 2 um. F) Schematic illustration of the FUBP1‐IN‐1 treatment regimen after intracardiac injection of PC‐3 cells in mice. G) Representative images of BLI, X‐ray, µCT, HE and TRAP staining of BM lesions in the indicated mice. Bars, 200 and 50 um. H) Quantification of bone metastasis burden based on BLI. I) Quantification of osteolytic area based on X‐ray. J) Quantification of TRAP+ OCs along the bone‐tumor interface of metastases. K) Kaplan‐Meier analysis for bone metastasis‐free survival. IN‐1, FUBP1‐IN‐1. All experiments were performed in biological triplicate. Statistical analyses were performed by Student's t‐test (H, I, J), one‐way ANOVA test (A, C, D, E), and the log‐rank test (K). *p < 0.05, **p < 0.001, and ***p < 0.0001.

The above results underscore the importance of the KDM5C/CCDC183‐AS1/FUBP1/LIGHT axis in the context of PCa BM, and warrant further investigation into the potential of these components as therapeutic targets against PCa BM. Notably, emerging studies suggest that FUBP1 may function as an oncogene,^[^ ^21^, ^25^ ^]^ as indirectly evidenced by its upregulation in multiple tumor types (Figure S9A, Supporting Information). FUBP1‐IN‐1, a novel small molecule inhibitor targeting FUBP1, exerts its mechanism of action by disrupting the interaction between FUBP1 and FUSE‐like DNA sequences. Therefore, we investigated the potential of FUBP1‐IN‐1 in the treatment of BM. The results in **Figure** **8**A,B and FS9B,C (Supporting Information) showed that FUBP1‐IN‐1 significantly inhibited LIGHT expression and reversed the promotive effect of CCDC183‐AS1 on LIGHT at both the mRNA and protein levels. Additionally, our luciferase reporter experiments demonstrated that FUBP1‐IN‐1 suppressed the activity of the wild‐type F4 reporter, while the mutant F4 reporter remained unaffected (Figure 8C). ChIP experiments further validated the inhibitory capacity of FUBP1‐IN‐1 in impeding the binding of FUBP1 at the P1 site, indicating that FUBP1‐IN‐1 disrupted the transcriptional regulatory activity of FUBP1 by obstructing its interaction with the FUSE‐like sequence (Figure 8C,D). Functionally, FUBP1‐IN‐1 significantly reduced PCa cell‐induced osteoclastogenesis by decreasing the count of TRAP^+^‐OCs and the area of bone absorption pits and inhibiting podosome formation (Figure 8E). Next, we assessed the potential therapeutic effects of FUBP1‐IN‐1 on PCa BM using an in vivo model (Figure 8F). Notably, compared to carrier therapy, FUBP1‐IN‐1 treatment markedly delayed BM onset and decreased BM burden, osteolytic area, and the count of TRAP^+^‐OCs (Figure 8G–K; Figure S9D,E, Supporting Information). Given that both lung cancer and breast cancer commonly exhibit bone metastasis, we further evaluated the potential of FUBP1‐IN‐1 to inhibit bone metastatic activity in vitro using A549 (lung cancer) and MDA‐MB‐231 (breast cancer) cell lines. The results indicated that FUBP1‐IN‐1 decreased the enrichment of FUBP1 on the TNFSF14 promoter and reduced the level of TNFSF14 (Figure S9F–I, Supporting Information). Meanwhile, FUBP1‐IN‐1 significantly suppressed lung and breast cancer cell‐induced osteoclastogenesis by decreasing the count of TRAP^+^‐OCs and the area of bone absorption pits and inhibiting podosome formation (Figure S9J–M, Supporting Information). In summary, our findings suggest that FUBP1‐IN‐1 shows promise in inhibiting BM and could offer a new therapeutic strategy for managing this condition.

### 2.10 Clinical Significance of the KDM5C/CCDC183‐AS1/FUBP1/LIGHT Axis in PCa

**Figure 9:**
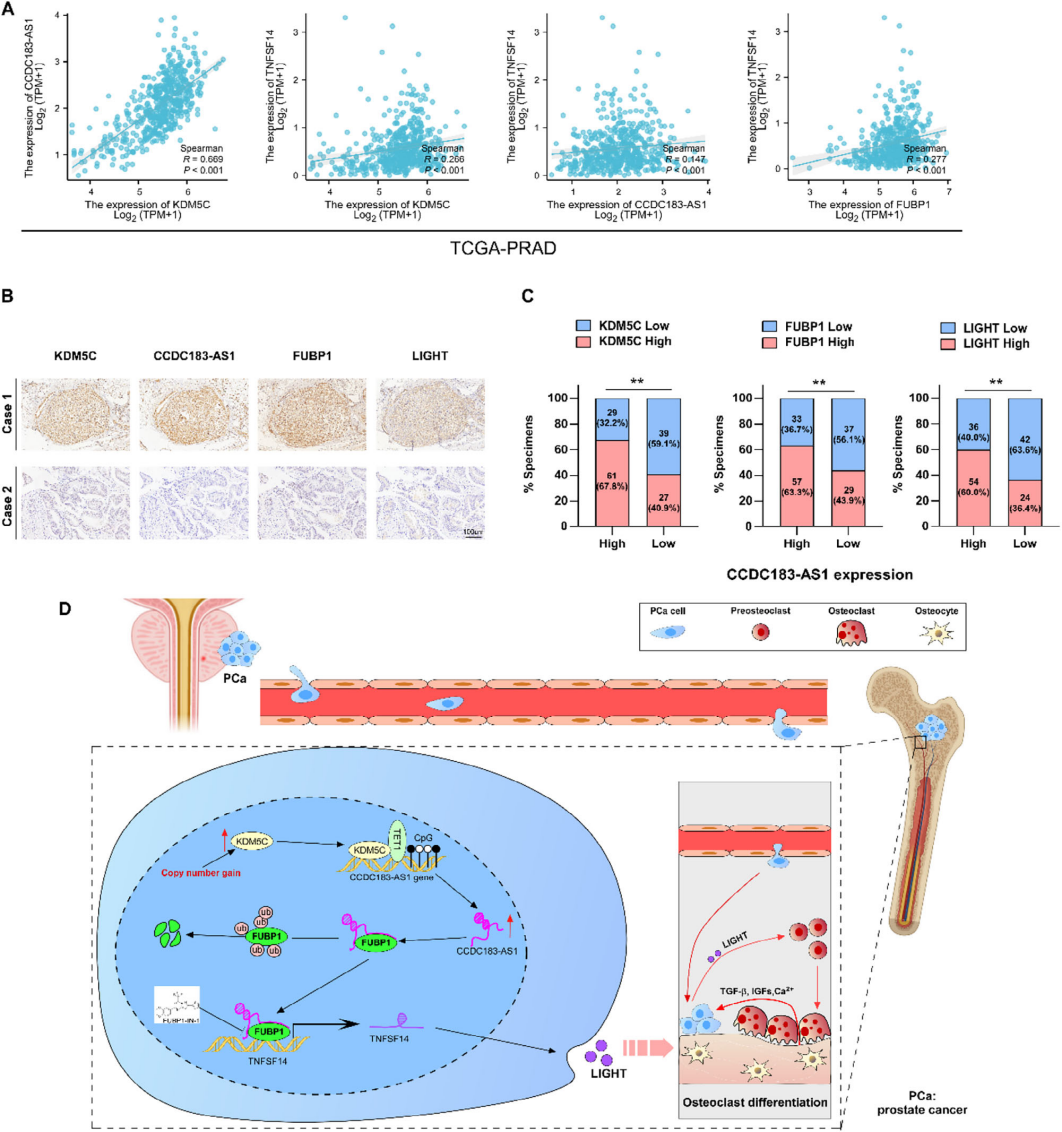
Clinical relevance of the KDM5C/CCDC183‐AS1/FUBP1/TNFSF14 axis in PCa. A) Analysis of the correlation between KDM5C, CCDC183‐AS1, FUBP1, and TNFSF14 based on the TCGA‐PRAD cohort. B) Representative images showing the expression of KDM5C, CCDC183‐AS1, FUBP1, and TNFSF14 in clinical PCa specimens. Bars, 100um. C) Percentage of specimens showing low or high CCDC183‐AS1 expression in relation to the expression levels of KDM5C, FUBP1, and LIGHT. D) Schematic diagram illustrating the regulatory mechanism of the KDM5C/CCDC183‐AS1/FUBP1/LIGHT axis in promoting PCa BM. All experiments were performed in biological triplicate. Statistical analyses were performed by Spearman correlation test (A) and χ2 test (C). *p < 0.05, **p < 0.001, and ***p < 0.0001.

To investigate the clinical significance of the KDM5C/CCDC183‐AS1/FUBP1/LIGHT axis, we explored the correlations between KDM5C, CCDC183‐AS1, FUBP1, and LIGHT in PCa based on the TCGA database and our cohort. The results shown in **Figure** **9**A revealed significant positive correlations between KDM5C and CCDC183‐AS1, KDM5C, and TNFSF14, CCDC183‐AS1 and TNFSF14, and FUBP1 and TNFSF14 expression levels. Interestingly, analysis based on the TCGA database revealed that TET1 expression was significantly correlated with the expression levels of both CCDC183‐AS1 and TNFSF14 (Figure S10A, Supporting Information). The findings from IHC and ISH indicated a positive correlation between the expression of CCDC183‐AS1 and the levels of KDM5C, FUBP1, and LIGHT (Figure 9B,C). Additionally, we verified the CCDC183‐AS1/FUBP1/LIGHT axis in primary PCa cells (Figure S10B, Supporting Information). Hence, our study conclusively demonstrated that copy number gain‐induced KDM5C drives the upregulation of CCDC183‐AS1 via TET1‐mediated DNA demethylation. CCDC183‐AS1 interacts with FUBP1 and inhibits FUBP1 degradation, which promotes the transcription of TNFSF14 and ultimately fosters BM in PCa (Figure 9D).

## 3 Discussion

BM is a complex multi‐step process.^[^ ^46^ ^]^ Throughout this procedure, the inherent ability of PCa cells to migrate and invade, their colonization of specific organs, and the alteration of the microenvironment at metastatic locations are crucial factors in organ‐specific metastasis.^[^ ^47^, ^48^, ^49^ ^]^ BM, a common and serious complication of advanced PCa, often results in a poor prognosis and reduced quality of life. Although there have been advancements in both personalized and systemic treatments for PCa, patients with BM still face reduced quality of life and survival rates due to complications associated with BM.^[^ ^20^ ^]^ Unfortunately, current treatment options for PCa BM are limited to palliative care, symptom alleviation, and supportive measures, and do not significantly prolong overall survival.^[^ ^50^ ^]^ Therefore, it is imperative to elucidate the mechanisms underlying PCa BM and establish effective treatment strategies. Here, we demonstrate that the KDM5C/CCDC183‐AS1/FUBP1 axis upregulates TNFSF14, leading to PCa BM. Notably, treatment with FUBP1‐IN‐1, a specific inhibitor of FUBP1, suppressed the CCDC183‐AS1‐induced osteoclastogenesis and effectively prevented the progression of BM. This may represent a promising new approach for clinical intervention in patients with BM.

In the current study, our results indicated a notable increase in LIGHT expression in patients with PCa BM and in CM from PCa cells overexpressing CCDC183‐AS1. Furthermore, the osteoclastogenic induction effect of CCDC183‐AS1 was abolished by interference with LIGHT expression. These results suggest that LIGHT is a key factor in CCDC183‐AS1‐induced BM in PCa. As an immune mediator associated with BM, LIGHT is closely linked with osteoclast activation and bone metastasis.^[^ ^51^, ^52^ ^]^ LIGHT contributes to increased bone resorption in several typical bone diseases, including erosive rheumatoid arthritis (RA) and multiple myeloma.^[^ ^53^, ^54^ ^]^ Consistent with our findings, Brunetti et al. showed that LIGHT was elevated in patients with non‐small cell lung cancer and BM, and played a critical role in osteoclastogenesis and BM‐induced bone resorption.^[^ ^55^ ^]^ Furthermore, in multiple myeloma, LIGHT synergistically stimulates osteoclastogenesis with RANKL, suggesting that elevated levels of LIGHT are detrimental to bone.^[^ ^56^ ^]^ In our study, we observed that the downregulation of LIGHT expression rescued CCDC183‐AS1‐induced osteoclastogenesis. Collectively, these findings suggest that LIGHT promotes bone destruction by inducing osteoclastogenesis, thereby providing potentially attractive molecular targets for treating PCa BM.

Similar to protein‐coding regulators, recent studies demonstrate that lncRNAs also exert a significant influence on tumor initiation and progression, showing considerable clinical potential in cancer diagnosis and treatment.^[^ ^15^ ^]^ Using publicly available data, we observed that CCDC183‐AS1 expression increased in PCa BM tissues, suggesting its potential involvement in the regulation of PCa BM. To date, the effects of CCDC183‐AS1 on PCa BM have not been investigated. Furthermore, the role of CCDC183‐AS1 in tumorigenesis remains relatively understudied, with reports limited to hepatocellular carcinoma, bladder cancer, and breast cancer. For instance, Zhu et al. demonstrated that CCDC183‐AS1 functions as a miR‐589‐5p decoy and promotes liver cancer cell growth and metastasis by modulating SKP1 expression.^[^ ^32^ ^]^ Similarly, in breast cancer, CCDC183‐AS1 promotes malignant progression by modulating the miR‐3918/FGFR1 regulatory axis.^[^ ^57^ ^]^ In addition, Mitf‐mediated lncRNA CCDC183‐AS1 has been implicated in bladder cancer tumorigenicity and aerobic glycolysis through the upregulation of TCF7L2.^[^ ^31^ ^]^ In our study, we observed that CCDC183‐AS1 competitively binds to FUBP1 with JTV‐1, inhibiting ubiquitin‐mediated degradation of FUBP1, and consequently increasing FUBP1 activity and expression. FUBP1 acts as a transcriptional regulator that facilitates LIGHT expression, thereby contributing to PCa‐BM development. Collectively, our findings reveal a novel mechanism by which CCDC183‐AS1 upregulates LIGHT secretion via interaction with FUBP1, highlighting the importance of CCDC183‐AS1 in PCa BM pathogenesis.

FUBP1 is an ATP‐driven DNA helicase V essential for controlling transcription, translation, and RNA splicing by attaching to single‐stranded DNA and RNA.^[^ ^22^ ^]^ There has been considerable research demonstrating that FUBP1 is upregulated in a variety of human cancers, suggesting its potential as a therapeutic and diagnostic target for these diseases. For example, FUBP1 enhances tumor progression by stimulating MYC transcription in esophageal squamous cell carcinoma.^[^ ^58^ ^]^ Wang et al. found that USF2‐mediated circACTN4 enhances MYC expression by interacting with FUBP1, thereby promoting the initiation and progression of breast cancer.^[^ ^21^ ^]^ In addition, studies have reported that the interaction between FUBP1 and NR_109 activates the transcription of MYC, and promotes M2‐like macrophage polarization, thereby affecting tumor cell proliferation and metastasis.^[^ ^35^ ^]^ Consistently, our data indicated that the binding of CCDC183‐AS1 to FUBP1 competitively inhibited JTV‐1‐induced ubiquitination and degradation of FUBP1. FUBP1 promotes osteoclastogenesis and PCa BM through upregulation of LIGHT Furthermore, the inhibition of FUBP1 binding to DNA with FUBP1‐IN‐1 suppressed osteoclastogenesis and BM, suggesting that FUBP1‐IN‐1 has potential therapeutic value in PCa BM. These findings provide a basis for future development of clinical drugs targeting PCa‐BM.

Through their catalytic activities, epigenetic modification enzymes shape the epigenetic landscape, thereby regulating gene expression. DNA methylation, an early identified epigenetic control mechanism, has been shown to influence RNA expression in cancers, including lncRNAs.^[^ ^59^ ^]^ For example, Lu et al. discovered that DNA methylation‐mediated activation of lncRNA SNHG12 promotes glioblastoma resistance to temozolomide through the miR‐129‐5p/MAPK1/E2F7 axis.^[^ ^60^ ^]^ In this study, we investigated the mechanism underlying CCDC183‐AS1 upregulation in PCa BM. Bioinformatic analysis revealed a significant correlation between CCDC183‐AS1 expression and DNA methylation of its promoter. We further investigated whether the expression of CCDC183‐AS1 was regulated by DNA methylation. We found that DNA methylation inhibited CCDC183‐AS1 expression in PCa cells, with KDM5C and TET1 playing critical roles. Consistent with our findings, Xu et al. found that YAP/TAZ recruits DNMT3A, leading to the hypermethylation and transcriptional silencing of the CDH1 promoter, which accelerates the transition from epithelial to mesenchymal cells, ultimately facilitating metastasis.^[^ ^43^ ^]^ Taken together, these results suggest that KDM5C‐ and TET1‐mediated DNA demethylation contribute to the upregulation of CCDC183‐AS1 expression in PCa BM. This finding helps to elucidate the functions of epigenetic changes that drive metastasis and provides new therapeutic principles for PCa BM.

Despite the promising findings of this study, several limitations should be acknowledged. First, the relatively small clinical sample size, inconsistencies in study samples, and retrospective nature of the analysis may limit the generalizability of the results. In future studies, a more robust study design would involve prospective patient enrollment, an expanded sample size, and selection of a homogeneous cohort to minimize confounding variables and improve statistical power. Second, intracardiac injection was used to establish a bone metastasis model in this study. Intracardiac injection is a commonly employed technique when utilizing human tumor cells, particularly for investigating the later stages of bone metastasis. This model enables the assessment of cancer cell arrest in bone, their subsequent survival, proliferation, and the formation of tumors that progress into cancer‐induced bone disease. However, intracardiac injection model recapitulates early steps in the metastatic process prior to embolism and entry of tumor cells into the circulation, and does not allow monitoring of tumor cells breaking away from the primary tumor into the bloodstream. In addition, there is currently also a lack of effective methods to specifically detect the process of tumor cells entering the circulatory system from the primary tumor site. Therefore, in future research, it is important to develop an animal model that can simulate the entire process of tumor cells metastasizing from the primary site to the bone, as well as to develop effective monitoring methods.

Overall, this study provides novel insights into the involvement of the KDM5C/CCDC183‐AS1/FUBP1/LIGHT axis in osteoclastogenesis and PCa BM. Our study not only reveals the mechanism of CCDC183‐AS1 in regulating FUBP1 ubiquitination degradation, but also validates FUBP1 as a potential therapeutic target for PCa BM, and suggests that FUBP1‐IN‐1 is promising as a FUBP1 inhibitor for the treatment of PCa BM.

## 4 Experimental Section

### Cell Culture

The LNCaP, PC‐3, 22RV1, VCaP, RWPE‐1, and DU145 cell lines were purchased from the Shanghai Chinese Academy of Sciences Cell Bank (Shanghai, China). The C4‐2B, RAW264.7, and 293FT cell lines were obtained from American Type Culture Collection (ATCC, Manassas, VA, USA). All human cell lines were cultured under the recommended conditions. All cell lines were cultured with the recommended medium supplemented with 10% fetal bovine serum (Life Technologies, USA), streptomycin (100 mg ml^−1^), and penicillin G (100 U ml^−1^) in a humidified atmosphere of 5% CO_2_ at 37 °C. All the cell lines were tested for mycoplasma contamination.

### Clinical Specimens

All tissue samples were collected from The First Affiliated Hospital of USTC and confirmed through histopathological diagnosis. The Institutional Research Ethics Committee at the First Affiliated Hospital of USTC approved the study protocol for utilizing clinical materials in research (No. 2023KY‐183). Every patient sample was collected following the Declaration of Helsinki guidelines, and each patient provided written informed consent for all procedures. Due to the retrospective nature of the study, the sample size was constrained by the availability of historical data. Therefore, all eligible cases were included in the analysis, and post hoc power analysis was performed to assess whether the statistical power achieved an acceptable level.

### Osteoclastogenesis Assay

The osteoclastogenesis assay was performed as previously described.^[^ ^61^ ^]^ RAW264.7 cells were seeded into 96‐well clusters at a density of 1 × 10^3^ cells well^−1^ and cultured with conditioned medium (CM, 500 ul) from the indicated PCa cells. CM supplemented with 25 ng ml^−1^ murine RANKL (No.:C28A, Novoprotein, Suzhou, China) was replaced every alternate day. After 6 days of culture, the cells were fixed with 4% paraformaldehyde/PBS (pH 7.4) and stained for OCs using a commercial kit (G1050‐50T; Servicebio). TRAP^+^ multinucleated cells with three or more nuclei were identified as OCs. Stained images were captured, and OCs were counted under a microscope. For each experimental group, nine images were obtained, and a representative subset of three images was randomly selected for data analysis.

### Plasmids and Transfection

Transfection and lentiviral packaging were performed employing Lipofectamine 3000 (Invitrogen, Waltham, MA) with validation of transfection efficiency through RT‐qPCR and western blot analysis. Lentivirus was generated by co‐transfecting HEK293FT cells with ViraPower Packaging Mix plasmids (pLP1, pLP2, and pLP/VSVG)(Thermo Fisher Scientific). Following a 48–72 h incubation, the harvested lentivirus‐infected PCa cells were utilized and were then selected with puromycin (ST551, Beyotime) to establish stable cell lines. Full‐length or mutated CCDC183‐AS1, Flag‐FUBP1, Myc‐JTV1, HA‐Ubiquitin, Flag‐KDM5C, and Myc‐TET1 were cloned into the pCDH‐puro vector. To silence FUBP1, KDM5C, and TET1, their respective human shRNAs inserted into pLKO.1 were constructed, and antisense oligonucleotides (ASOs) targeting the CCDC183‐AS1 were sourced from Ribo (Guangzhou). The sequences of ASOs and shRNAs are provided in the Table S2 (Supporting Information).

### Animal Study

All animal experiments were approved by the Institutional Animal Care and Use Committee of the First Affiliated Hospital of USTC (No. 2023‐N(A)‐004). The BM animal model was constructed by left ventricular injection as described in previous studies,^[^ ^62^ ^]^ and the therapeutic effects of ASO targeting CCDC183‐AS1, and FUBP1‐IN‐1 were assessed through bioluminescent imaging (BLI) system, X‐ray, µCT, hematoxylin and eosin (HE), and immunohistochemistry (IHC) staining analyses. For the animal model of bone metastasis, BALB/c‐nu mice (male, 4–6 weeks old) were injected with PC‐3 cells (1 × 10^6^) in 100 ul PBS into the left cardiac ventricle. Mice were anesthetized with isoflurane (inhalation anesthesia, 3% for induction and 1.5% for maintenance), and at the end of the experiment, the mice were euthanized with CO_2_ inhalation (CO_2_ replacement rate, 30%). One week after injection, the mice were randomly assigned to every group (n = 8 per group). ASO (10 nmol in 100 µl PBS) or PBS (100 µl) was administered via tail vein injection every 5 days for a total of 6 doses. FUBP1‐IN‐1 (HY‐100758, 5 mg kg^−1^ in 200 µl DMSO) or DMSO (200 µl) was also injected via the tail vein every 3 days for a total of 15 doses. The number of mice used in every group was determined based on a priori power analysis using G*Power software, assuming a medium effect size (Cohen's d = 0.5), a power of 0.8, and an alpha level of 0.05. To account for possible loss during the experiment, 8 animals were assigned to each group. This approach ensured both scientific validity and compliance with ethical guidelines regarding animal use. The number of experimental groups was kept to a minimum while ensuring that the results were sufficient to support the conclusions.

### Statistical Analysis

All data in this study were presented as mean ± standard deviation (SD) and statistically analyzed using GraphPad Prism 9.0 (GraphPad Software, San Diego, CA, USA) and SPSS 19.0 statistical software (SPSS Inc., Chicago, IL, USA). The value of n represents the number of independent experiments conducted using distinct biological replicates, including separate mice, independent cell batches, or individual clinical tissue samples. Statistical analysis was performed using the Student's two‐tailed t‐test and one‐way analysis of variance (ANOVA). The Spearman's correlation coefficient was calculated to explore the bivariate correlations between the different study variables. Survival curves were generated using the Kaplan‐Meier method and compared using the log‐rank test. Normality was assessed using the Shapiro‐Wilk test for sample sizes less than 50 and the Kolmogorov‐Smirnov test for sample sizes greater than or equal to 50. Unless otherwise indicated in the figure legends, all experiments were independently repeated at least three times under comparable experimental conditions. Statistical significance was set at *p* < 0.05.

## Conflict of Interest

The authors declare no conflict of interest.

## Author Contributions

C.L., X.M., K.C., and X.W. contributed equally to this work. C.L., Y.D., W.Z., C.Y., and J.X. developed the original idea and designed the experiments. C.L., X.M., K.C., and X.W. prepared the figures and drafted the manuscript. C.L., Z.Y., J.W., and Y.R. conducted the experiments and contributed to data analysis. W.Z. and J.X. provided critical reagents and/or clinical samples. C.L. and W.Z. supervised the study. All authors contributed to revising the manuscript and approved the final version for publication.

## Supporting information



Supporting Information

Supporting Information

## Data Availability

The data that support the findings of this study are available on request from the corresponding author.
