# Supplementary material for: Epigenetic Activation of CCDC183‐AS1 Promotes Osteoclastogenesis and Prostate Cancer Bone Metastasis Through the FUBP1/LIGHT Axis
Source: Adv Sci (Weinh). 2025 Jul 20;12(38):e13288. doi: 10.1002/advs.202413288 (PMC12520472; doi:10.1002/advs.202413288)
Supplement: Supplementary file 1 — Supporting Information [file ADVS-12-e13288-s001.docx]

**Supporting Information**

**Epigenetic activation of CCDC183-AS1 promotes osteoclastogenesis and prostate cancer bone metastasis through the FUBP1/LIGHT axis.**

Chuandong Lang^1#^, Xiangyu Mu^2#^, Kun Chen^1#^, Xinwen Wang^3#^, Yuluo Rong^1^, Jia Wang^1^, Zongcheng Yang^4^, Chi Yin^5^*, Yuhu Dai^3^*, Jun Xiao ^2^*, Wenzhi Zhang^1^*

**Supplementary Figures and Legends**

**
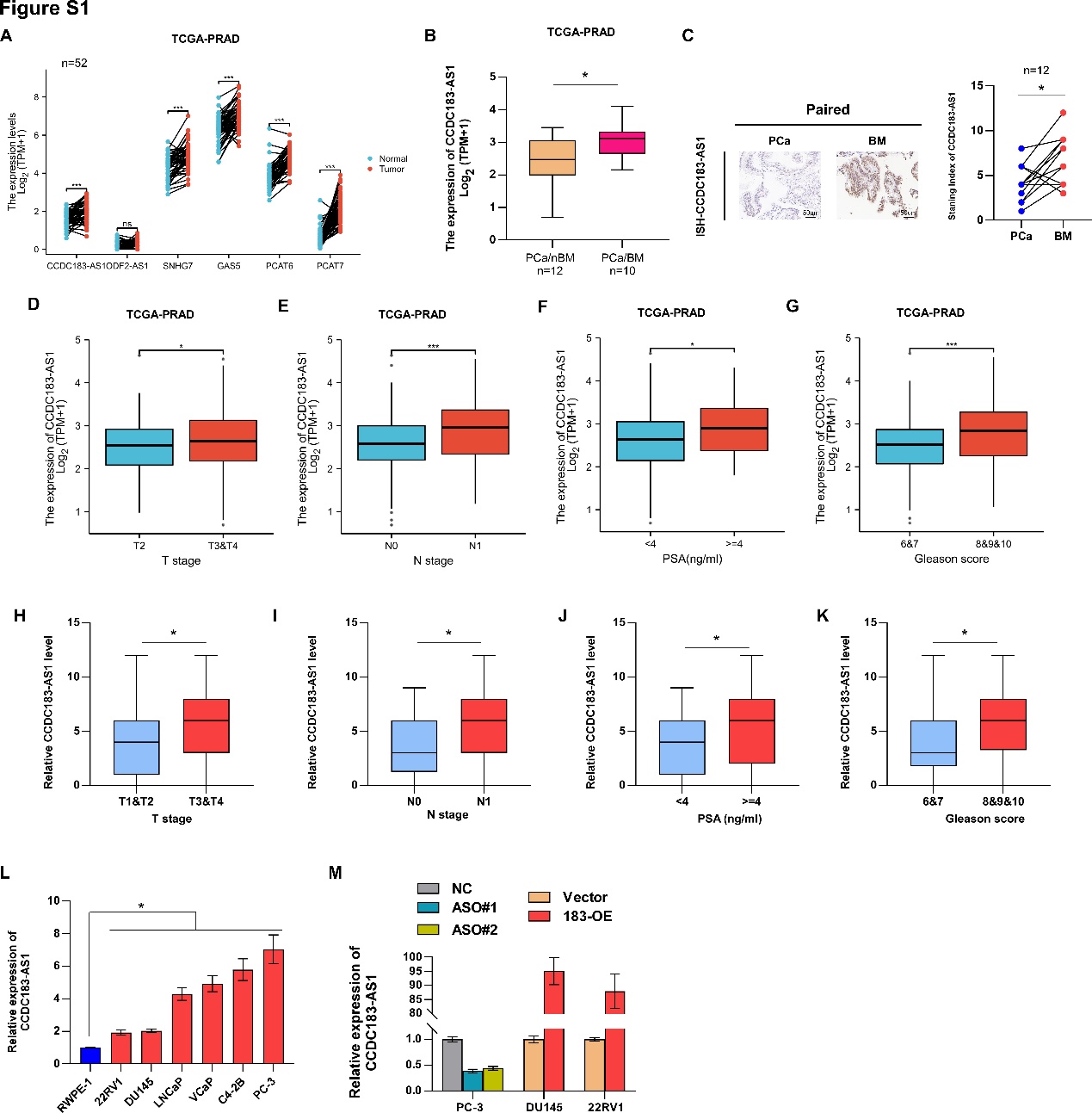
**

**Figure S1. CCDC183-AS1 overexpression is related to BM in PCa.**

**A** Comparison of six lncRNAs expression associated with PCa BM in PCa and normal tissue based on the TCGA-PRAD dataset. **B** Comparison of CCDC183-AS1 expression in PCa/nBM (n=12) and PCa/BM (n=10) based on the TCGA-PRAD dataset. **C** Representative ISH analysis images and quantification of CCDC183-AS1 expression in paired primary PCa tissue and BM tissue (n=12). **D-G** Analysis of the relationship between CCDC183-AS1 expression and T stage, N stage, PSA levels, and Gleason score in PCa based on the TCGA-PRAD cohort. **H-K** Analysis of the relationship between CCDC183-AS1 expression and T stage, N stage, PSA levels, and Gleason score in PCa based on our cohort. **L** Expression of CCDC183-AS1 in PCa cell lines and normal cell lines. **M** Validation of CCDC183-AS1 inhibition and overexpression efficiency using antisense oligonucleotides (ASOs, 5uM, 24h) and CCDC183-AS1 plasmid (183-OE). All experiments were performed in biological triplicate. Statistical analyses were performed by Student’s t-test (A-K) and one-way ANOVA test (L, M). **p*<0.05, ***p*<0.001, ****p*<0.0001.


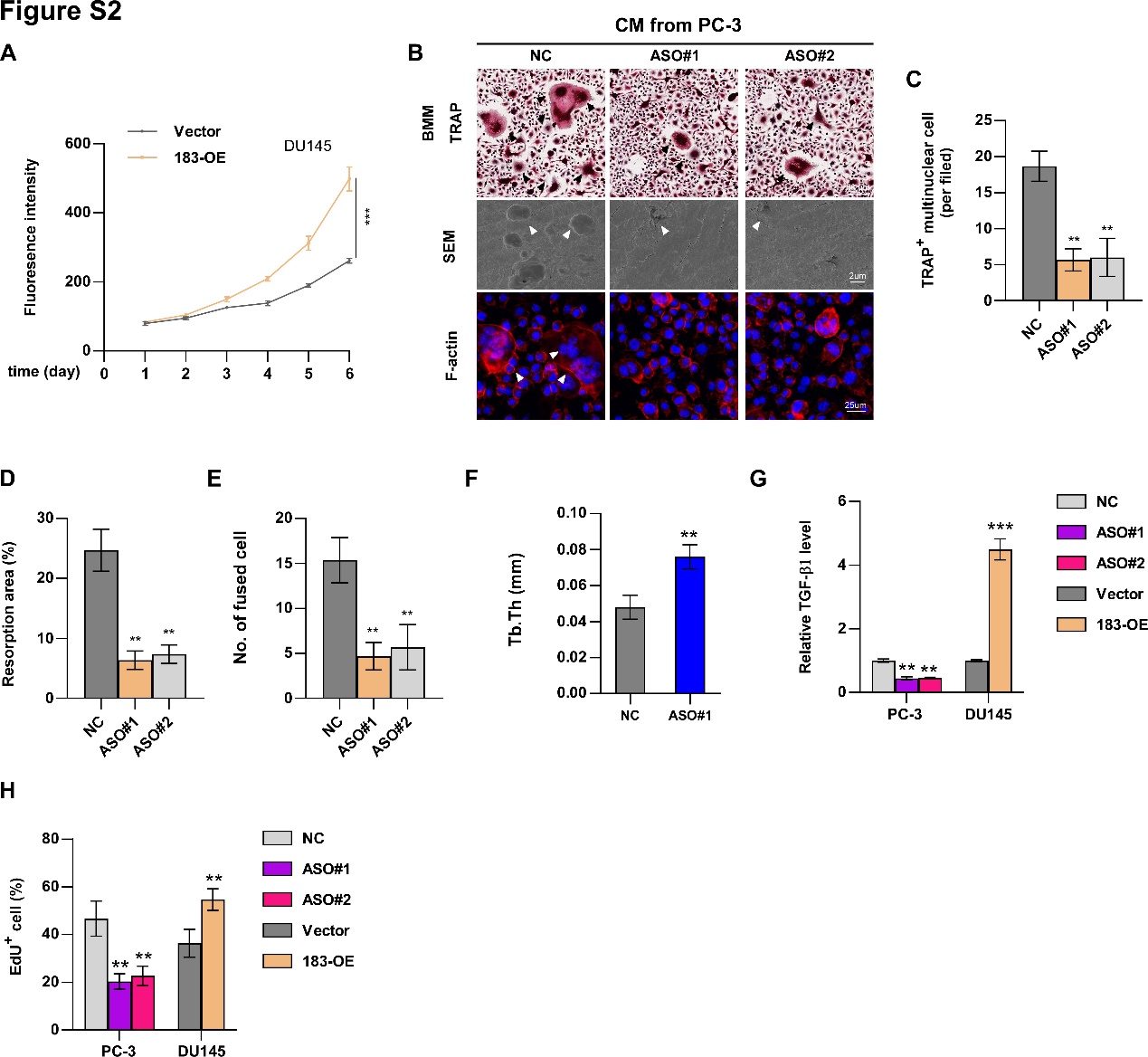


**Figure S2. CCDC183-AS1 overexpression promotes osteoclastogenesis.**

**A** Measurement of fluorescence intensity of the culture supernatant of RAW264.7 cells treated with CM from PCa cell. **B** Osteoclast differentiation assay using TRAP staining (top) and Bone resorption assay following SEM (middle) and formation of actin rings using F-actin staining (bottom) after bone marrow macrophage (BMM) with treatment of CM from the indicated PCa cells. **C-E** Quantification of TRAP+ multinucleated osteoclasts (C), the area of resorption pit per bone slice (D) and fused multinucleated cells (E) after bone marrow macrophage (BMM) with treatment of CM from the indicated PCa cells. **F** Quantification of Tb.Th (mm) in the indicated mice. **G** Analysis of TGF-β1 levels in the indicated medium using ELISA. **H** Quantification of EdU-positive cells. All experiments were performed in biological triplicate. Statistical analyses were performed by Student’s t-test (A, F) and one-way ANOVA test (C, D, E, G, H), and the log-rank test (N). **p*<0.05, ***p*<0.001, ****p*<0.0001.


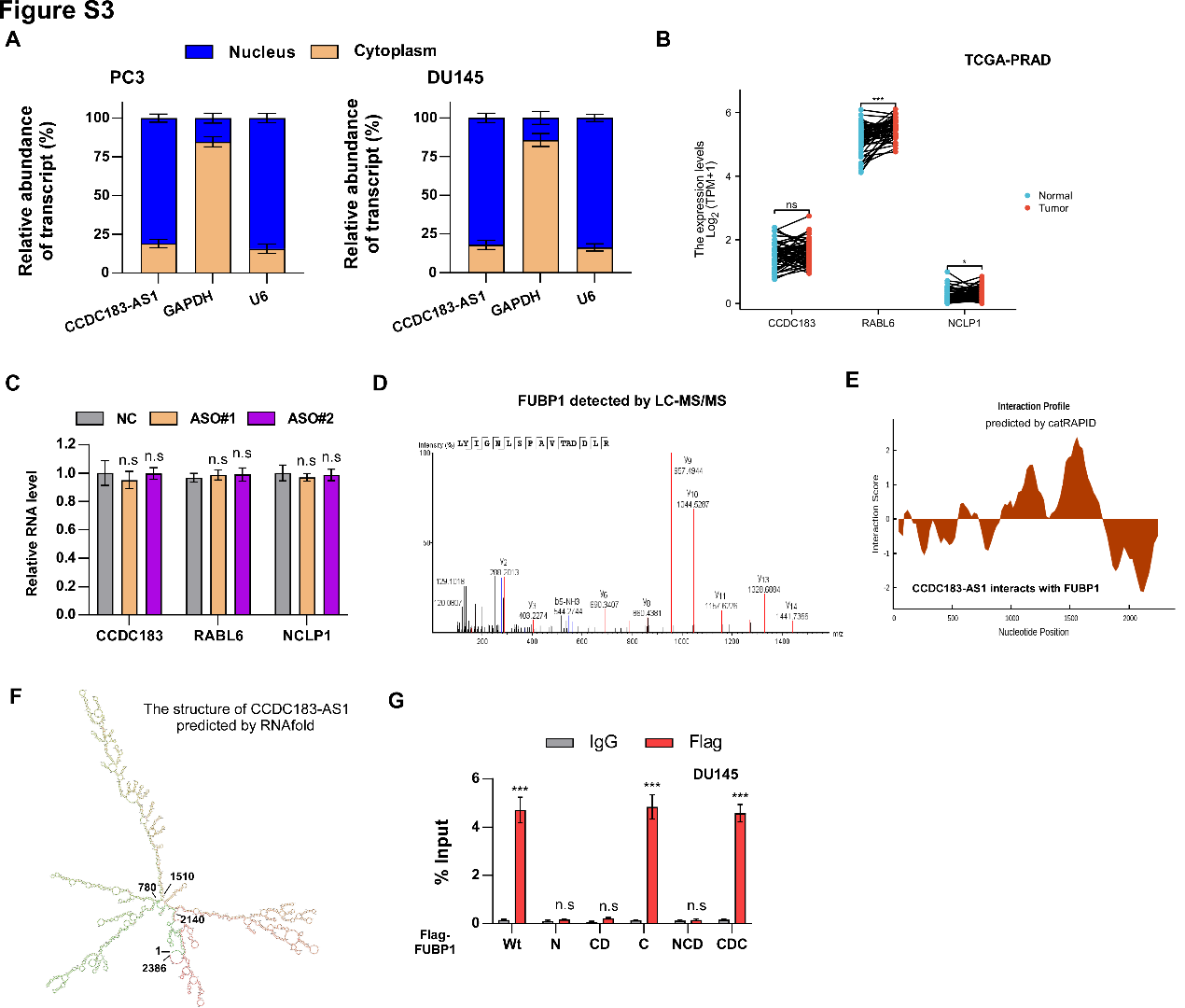


**Figure S3. CCDC183-AS1 interacts with FUBP1 protein.**

**A** The nuclear-cytoplasmic fractionation assay demonstrates that CCDC183-AS1 is mainly in the nucleus of PCa cells. U6 and GAPDH are utilized as positive controls for the nucleus and cytoplasm, respectively. **B** Scatter plot displaying the expression of genes adjacent to the CCDC183-AS1 locus in tumor and normal tissues based on the TCGA-PRAD dataset, including CCDC183, RABL6 and NCLP1. **C** RT-qPCR analysis showing that interference with CCDC183-AS1 does not significantly alter the expression of CCDC183, RABL6, and NCLP1. **D** LC-MS/MS profiles of target band (corresponding peptide sequences of FUBP1) retrieved by CCDC183-AS1. **E** Prediction of the interaction between CCDC183-AS1 and FUBP1 using the catRAPID website. **F** Secondary structure prediction of CCDC183-AS1 using the RNAfold website. **G** RIP assay using anti-flag and control IgG antibodies to detect the enrichment of CCDC183-AS1. All experiments were performed in biological triplicate. Statistical analyses were performed by Student’s t-test (B, G) and one-way ANOVA test (C). **p*<0.05, ***p*<0.001, ****p*<0.0001.


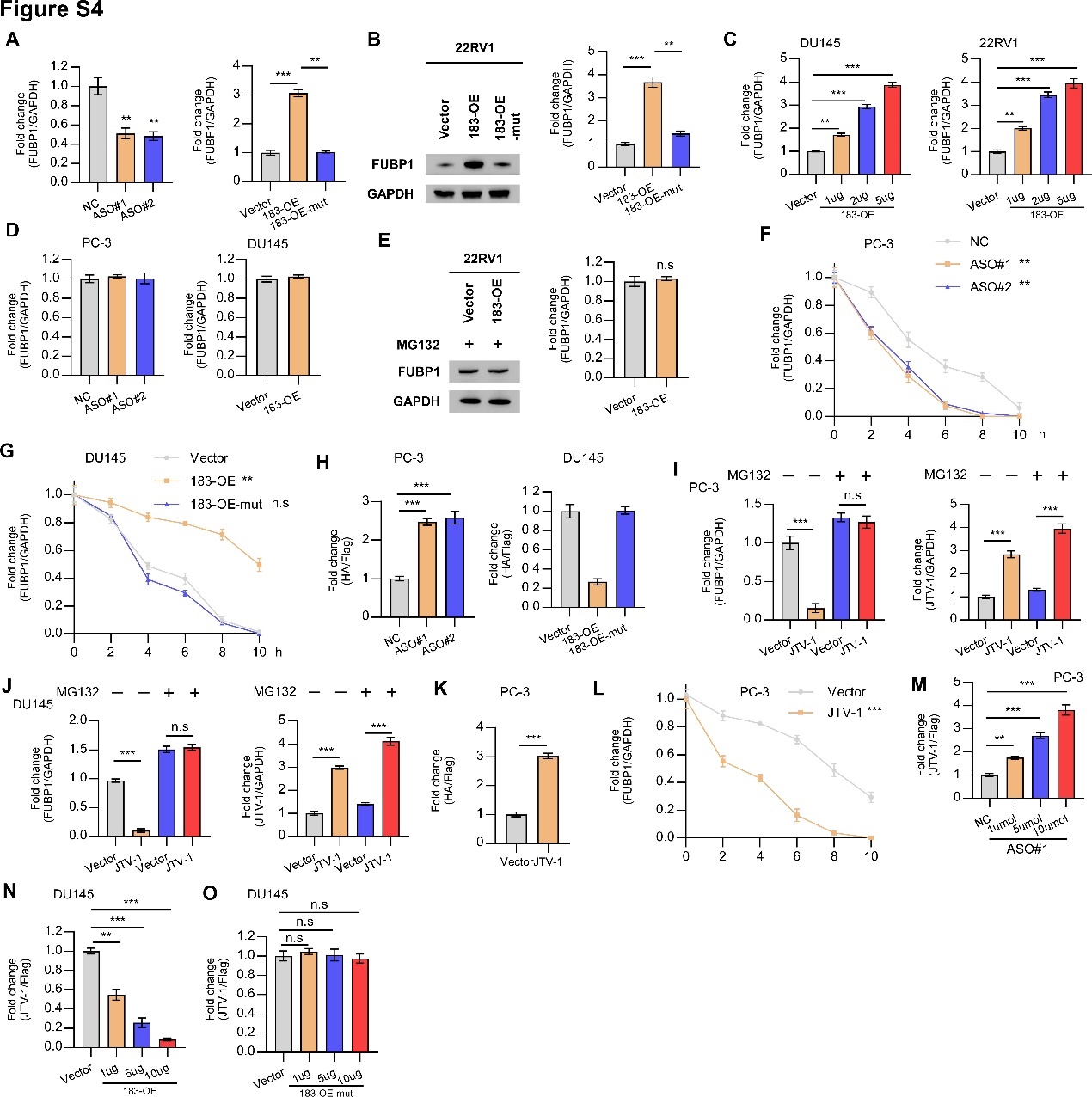


**Figure S4. CCDC183-AS1 blocks ubiquitin-mediated degradation of FUBP1 via competing with JTV-1.**

**A** Quantitative analyses of fold change (FUBP1/GAPDH) from western blot assay. **B** The effect of CCDC183-AS1 overexpression on FUBP1 protein and quantitative analyses. **C** Quantitative analyses of fold change (FUBP1/GAPDH) from western blot assay. **D** Quantitative analyses of fold change (FUBP1/GAPDH) from western blot assay. **E** Alterations in FUBP1 protein expression in the specified cells following treatment with the proteasome inhibitor MG132 and quantitative analyses. **F-G** Quantitative analyses of fold change (FUBP1/GAPDH) from western blot assay. **H** Quantitative analyses of fold change (HA/Flag) from western blot assay. **I-J** Quantitative analyses of fold change (FUBP1/GAPDH, JTV-1/GAPDH) from western blot assay. **K** Quantitative analyses of fold change (HA/Flag) from western blot assay. **L** Quantitative analyses of fold change (FUBP1/GAPDH) from western blot assay. **M-O** Quantitative analyses of fold change (JTV-1/GAPDH) from western blot assay. All experiments were performed in biological triplicate. Statistical analyses were performed by Student’s t-test (E, K, L) and one-way ANOVA test (A, B, C, D, F, G, H, I, J, M, N, O). **p*<0.05, ***p*<0.001, ****p*<0.0001.


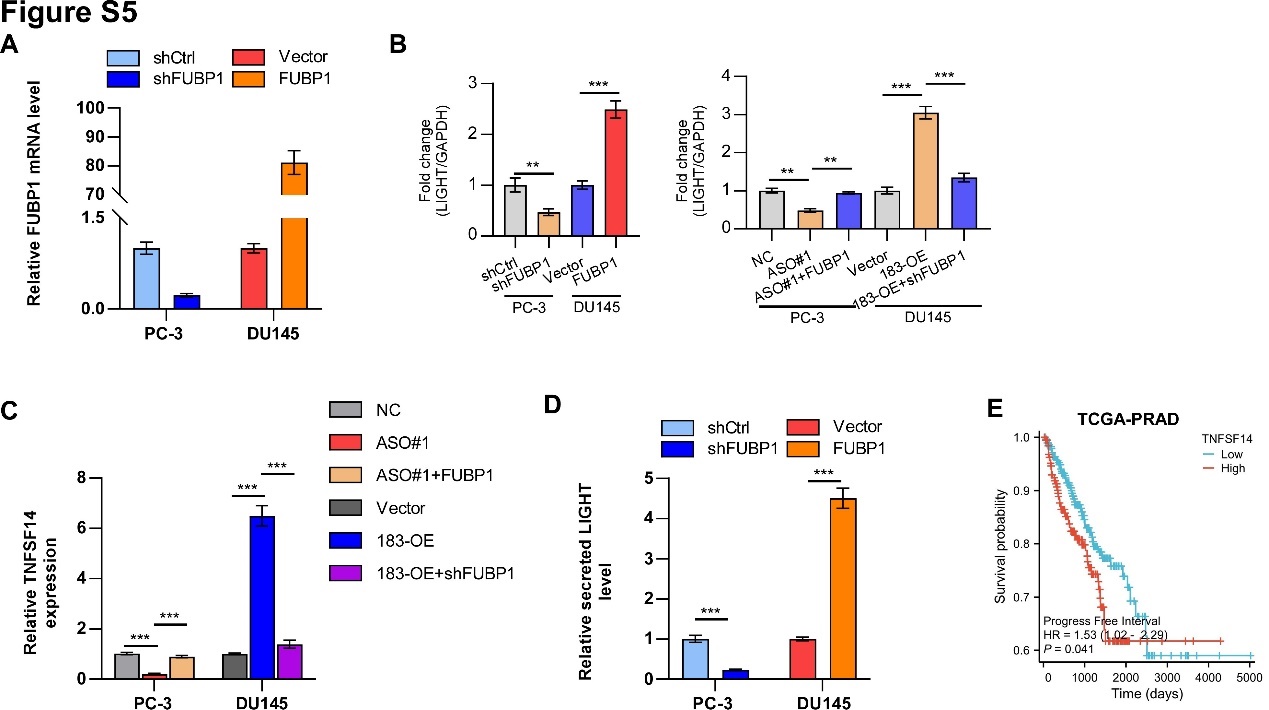


**Figure S5. CCDC183-AS1 induced LIGHT promoting osteoclastogenesis in an FUBP1-dependent manner.**

**A** Validation of FUBP1 knockdown and overexpression efficiency using RT-qPCR. **B** Quantitative analyses of fold change (LIGHT/GAPDH) from western blot assay. **C** RT-qPCR showing the effect of altered CCDC183-AS1 and FUBP1 expression on TNFSF14 mRNA. **D** ELISA showing the effect of changed FUBP1 expression on LIGHT secretion levels. **E** Analysis of the relationship between TNFSF14 and prognosis in PCa patients based on the TCGA-PRAD cohort. **F** Quantification of trabecular thickness (Th.Tb) in uCT from the indicated mice. All experiments were performed in biological triplicate. Statistical analyses were performed by Student’s t-test (B-left, D) and one-way ANOVA test (B-right, C), and the log-rank test (E). **p*<0.05, ***p*<0.001, ****p*<0.0001.


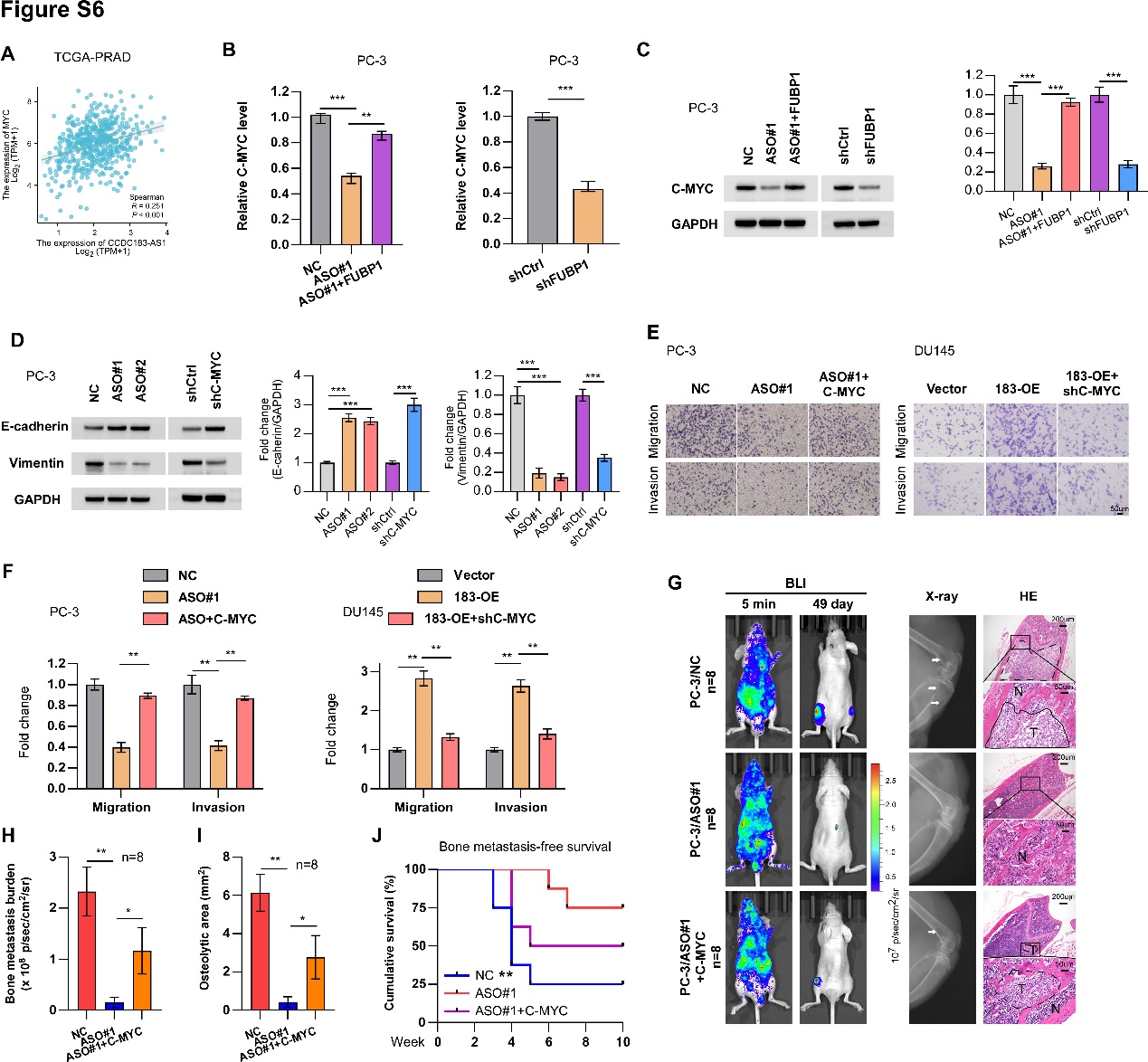


**Figure S6. CCDC183-AS1 induces EMT of PCa cells through FUBP1/C-MYC axis.**

**A** Correlation analysis between CCDC183-AS1 expression and C-MYC expression in PCa patients based on the TCGA-PRAD cohort. **B** RT-qPCR analysis of C-MYC expression in the indicated cells. **C** Western blot assay of C-MYC expression in the indicated cells and quantitative analyses. **D** Western blot assay of E-cadherin and vimentin expression in the indicated cells and quantitative analyses. **E-F** Representative images of transwell assay in the indicated cells and quantitative analyses. **G** Representative BLI images, X-ray images and HE images of bone metastasis lesions in mice. Bars, 200um, 50um. **H** Quantification of bone metastasis burden based on BLI. **I** Quantification of osteolytic areas based on X-ray. **J** Kaplan-Meier analysis showing bone metastasis-free survival curve of mice from three groups. All experiments were performed in biological triplicate. Statistical analyses were performed by Student’s t-test (B-right, F, G, H), one-way ANOVA test (B-left, C, D, F, H, I), Spearman correlation test (A) and the log-rank test (J). **p*<0.05, ***p*<0.001, ****p*<0.0001.


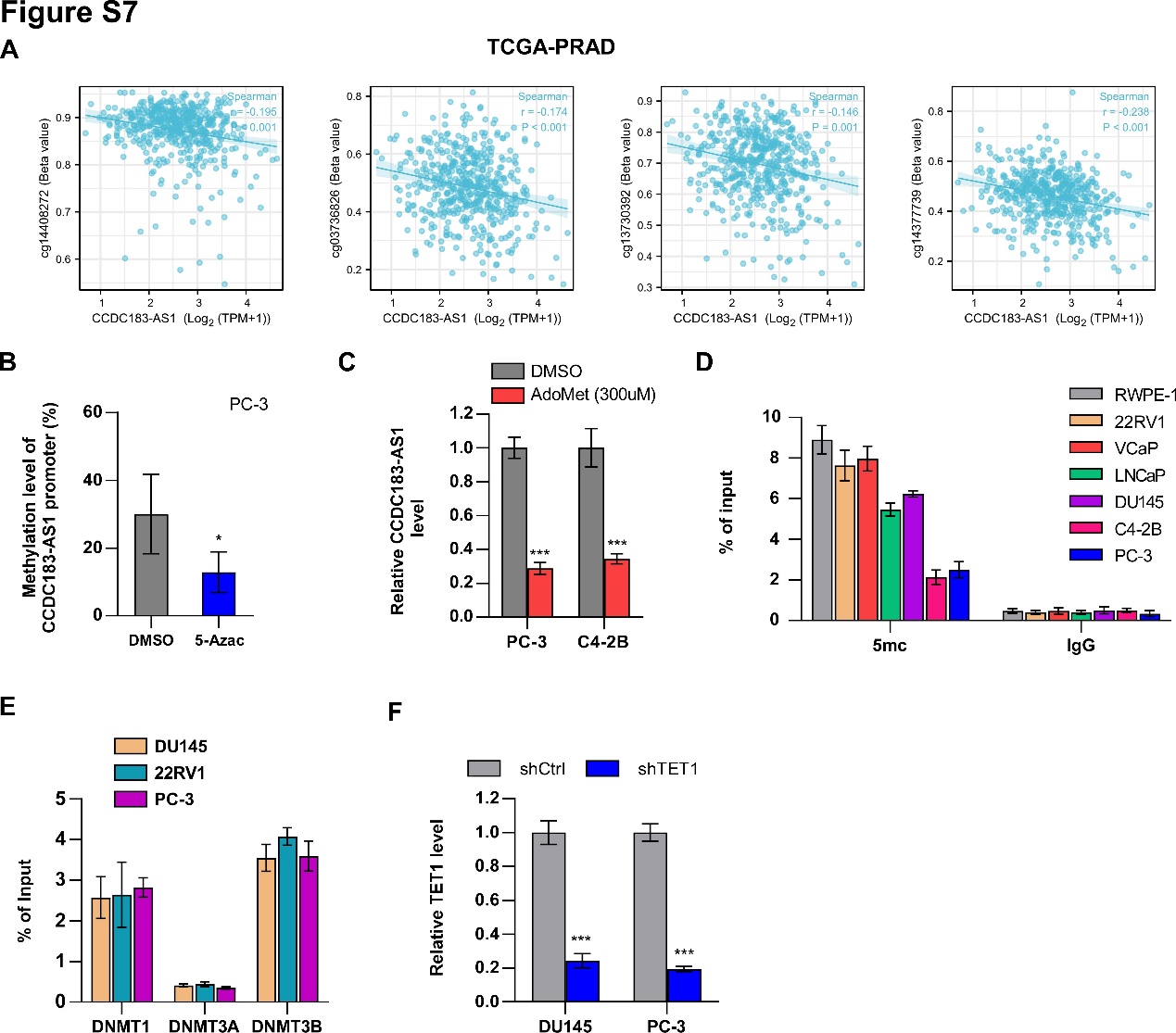


**Figure S7. Elevated CCDC183-AS1 expression is linked to hypomethylation of its own promoter.**

**A** Correlation analysis between CCDC183-AS1 expression and methylation level of different CpG sites in PCa patients based on the TCGA-PRAD cohort. **B** BSP assay showing the methylation level of CCDC183-AS1 promoter in PC3 cells. **C** RT-qPCR examination of the effect of AdoMet (300uM) treatment for 72h on CCDC183-AS1 expression in PCa cells. **D** ChIP analysis to assess the enrichment level of 5mc on the CCDC183-AS1 promoter. **E** ChIP analysis to evaluate the enrichment level of DNMT1, DNMT3A and DNMT3B on the CCDC183-AS1 promoter. **F** RT-qPCR examination of TET1 level in the indicated cells. All experiments were performed in biological triplicate. Statistical analyses were performed by Student’s t-test (B, C, F) and Spearman correlation test (A). **p*<0.05, ***p*<0.001, ****p*<0.0001.


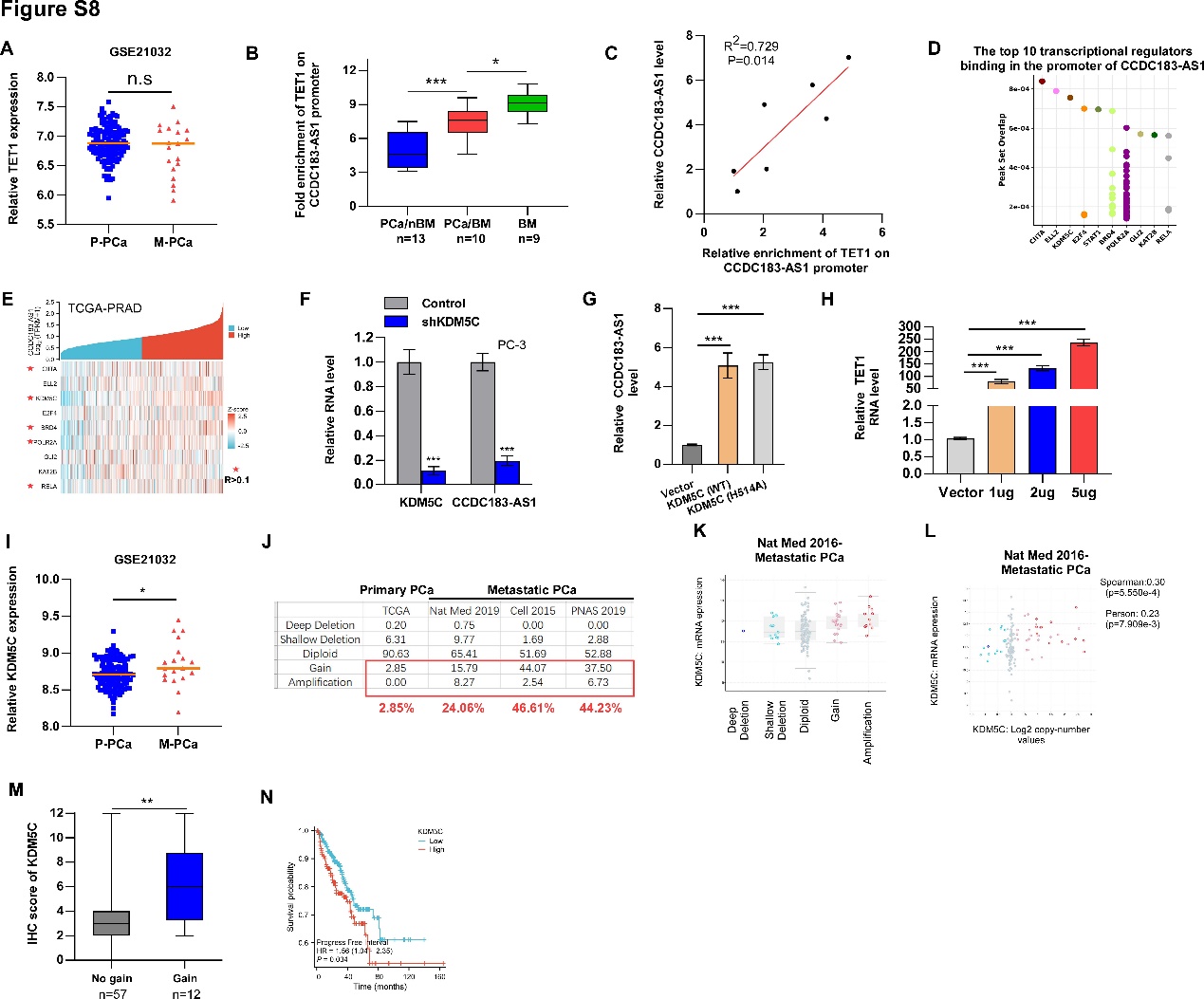


**Figure S8. KDM5C recruits TET1 to the promoter region of CCDC183-AS1, promoting DNA demethylation of CCDC183-AS1.**

**A** Analysis of TET1 expression in primary PCa and metastatic PCa tissues using the GSE21032 dataset. **B** ChIP assay showing the TET1 enrichment at the promoter region of CCDC183-AS1 in PCa/nBM (n=13), PCa/BM (n=10), and BM (n=9) tissues. **C** The correlation analysis of the enrichment of TET1 on CCDC183-AS1 promoter and the CCDC183-AS1 level. **D** Identification of the top 10 transcriptional regulators binding to the promoter of CCDC183-AS1 by the Cistrome Data Browser website. **E** Heatmap showing the correlation between the top 10 transcriptional regulators and CCDC183-AS1 in the TCGA-PRAD dataset. **F** RT-qPCR assay showing the effect of KDM5C knockdown on CCDC183-AS1 expression in PC3 cells. **G** RT-qPCR assay showing the effect of KDM5C (WT) and KDM5C (H514A) on CCDC183-AS1 expression in PC3 cells. **H** RT-qPCR examination of TET1 level in the indicated cells. **I** Analysis of KDM5C expression in primary PCa and metastatic PCa tissues using the GSE21032 dataset. **J** Analysis of KDM5C copy number gain in primary PCa and metastatic PCa based on published datasets. **K** KDM5C expression in patients with different copy number (Nat Med. 2016 Mar;22(3):298-305). **L** Correlation between KDM5C copy number value and mRNA expression in the metastatic PCa cohort (Nat Med. 2016 Mar;22(3):298-305). **M** IHC score of KDM5C in PCa patients with or without KDM5C copy number gain. **N** Analysis of the relationship between KDM5C and prognosis in PCa patients based on the TCGA-PRAD cohort. All experiments were performed in biological triplicate. Statistical analyses were performed by Student’s t-test (A, F, I, M), one-way ANOVA test (B, G, H, K), Spearman correlation test (C, L) and the log-rank test (N). **p*<0.05, ***p*<0.001, ****p*<0.0001.


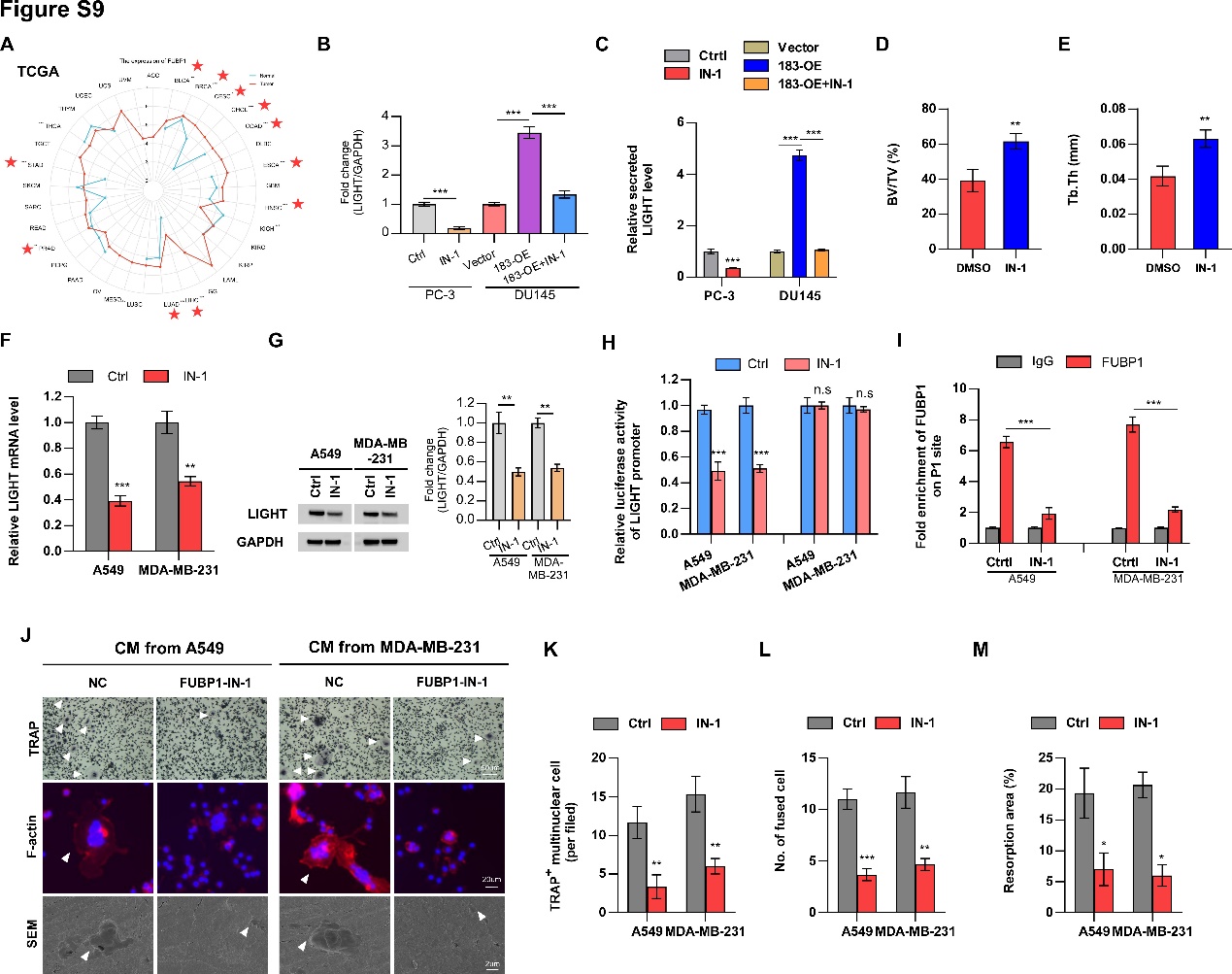


**Figure S9. FUBP1-IN-1 inhibits CCDC183-AS1-induced osteoclastogenesis in vitro and PCa BM in vivo.**

**A** Radar map showing comprehensive analysis of FUBP1 expression among various tumor tissues and normal controls using the TCGA public database. **B** Quantitative analyses of fold change (LIGHT/GAPDH) from western blot assay. **C** The effect of FUBP1-IN-1 (IN-1) on the secretion level of LIGHT protein. **D-E** Quantification of bone volume fraction/total volume (BV/TV) **(D)** and Th.Tb **(E)** in uCT from the indicated mice. **F** RT-qPCR analysis of LIGHT level in the indicated cells. **G** Western blot analysis of LIGHT level in the indicated cells and quantitative analyses. **H** Dual-luciferase reporter assay to measure the effect of FUBP1-IN-1 (20uM, 24 hours) on LIGHT promoter activity in the indicated cells. **I** ChIP analysis to determine the effect of FUBP1-IN-1 on the enrichment of FUBP1 on TNFSF14 promoter (P1 site) (20uM, 24 hours) in the indicated cells. **J-M** TRAP assay, F-actin staining and SEM showing the effect of FUBP1-IN-1 on osteoclast differentiation and activation *in vitro* in the indicated cells and quantitative analyses. Bars, 50um, 20um, 2um. All experiments were performed in biological triplicate. Statistical analyses were performed by Student’s t-test (A, D, E, F, G, H, I, K, L, M) and one-way ANOVA test (B, C). **p*<0.05, ***p*<0.001, ****p*<0.0001.


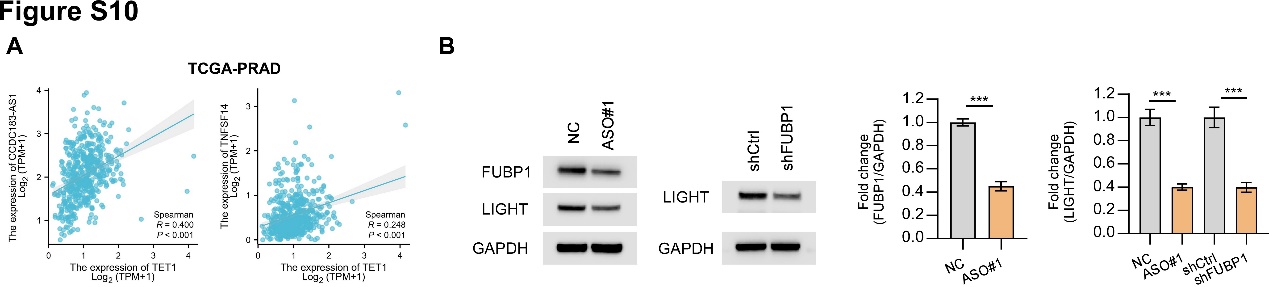


**Figure S10 Clinical relevance of the KDM5C/CCDC183-AS1/FUBP1/TNFSF14 axis in PCa.**

**A** Analysis of the correlation betweenTET1, CCDC183-AS1, and TNFSF14 based on the TCGA-PRAD cohort. B Western blot analysis of FUBP1 and LIGHT level in the primary PCa cells and quantitative analyses. All experiments were performed in biological triplicate. Statistical analyses were performed by Student’s t-test (B) and Spearman correlation test. **p*<0.05, ***p*<0.001, ****p*<0.0001.

**Supplementary Materials and Methods**

**Transwell assay**

Cell migration and invasion assays were performed using Transwell chambers (Corning, NY, USA) with 8.0 μm pore size membranes. For the invasion assay, the upper surface of the membrane was coated with Matrigel (BD Biosciences, CA, USA) diluted in serum-free medium (1:8 dilution), while the migration assay was conducted without Matrigel coating. Briefly, cells were starved in serum-free medium for 24 hours prior to the assay and then resuspended in serum-free medium at a density of 5 × 10⁴ cells/mL (for migration) or 1 × 10⁵ cells/mL (for invasion). A volume of 200 μL of the cell suspension was added to the upper chamber, while 600 μL of complete medium containing 10% fetal bovine serum (FBS) was placed in the lower chamber as a chemoattractant. Following incubation at 37 °C with 5% CO₂ for 24–48 hours, non-migrating or non-invading cells on the upper surface of the membrane were removed with a cotton swab. The migrated or invaded cells on the lower surface were fixed with 4% paraformaldehyde for 15 minutes, stained with 0.1% crystal violet solution for 30 minutes, and washed three times with PBS. Images of five random fields per membrane were captured under an inverted microscope (Olympus, Tokyo, Japan) at 100× magnification.

**Immunoprecipitation assay (IP)**

The cells were washed three times with PBS. Then, they were collected and lysed using cold freeze-thaw lysis buffer (Tris-HCl, EDTA, and 1% Triton X-100) containing PMSF (ST506, Beyotime) and a cocktail of proteinase inhibitors (4693116001, Roch). Subsequently, the lysates were incubated with 50 μl of anti-Flag (P2115, Beyotime), Myc (P2118, Beyotime), HA (P2121, Beyotime) or anti-mouse IgG (P2171, Beyotime) magnetic beads at 4°C for 12 hours. Following this, the mixture was washed six times with washing buffer and eluted with elution buffer for Western blot analysis. Conformation-specific IgG secondary antibodies (#3678, CST, Danvers, MA, USA) were used to remove heavy- or light-chains.

**RNA immunoprecipitation (RIP)**

For RIP, the Magna RIP RNA-Binding Protein Immunoprecipitation Kit (Millipore, Billerica, MA, USA) was utilized, and the experimental procedures were conducted following the manufacturer's instructions. In brief, cells were cross-linked twice with 100,000 μJ/cm^2^ UV, followed by lysis in RIP lysis buffer containing 0.2% protease inhibitor Cocktail and 0.1% RNase inhibitor, and subsequent immunoprecipitation. The immunoprecipitated complex was then subjected to proteinase K digestion, followed by RNA extraction, and RT-qPCR was employed to assess the RNA expression.

**ChIP assay**

Chromatin immunoprecipitation (ChIP) assay was performed according to the manufacturer’s instructions (SimpleChIP Plus Enzymatic Chromatin IP Kit, 9005S, Cell Signaling Technology) with minor modifications. Briefly, cells were cross-linked with 1% formaldehyde at room temperature for 10 minutes to fix protein-DNA interactions, followed by quenching with glycine (final concentration 0.125 M). After washing with cold PBS, cells were lysed in lysis buffer and chromatin was digested with micrococcal nuclease (MNase) to generate DNA fragments of approximately 150–900 bp. The chromatin lysate was incubated overnight at 4 °C with rotation using specific antibodies against FUBP1, TET1, KDM5C or normal rabbit IgG as a negative control. Immune complexes were captured using protein G magnetic beads, and cross-links were reversed by incubation at 65 °C for 2 hours. Following proteinase K digestion and DNA purification, enriched DNA fragments were analyzed by quantitative real-time PCR (qPCR) using primers specific to the TNFSF14 or CCDC183-AS1. Data were normalized to input DNA and presented as fold enrichment relative to the IgG control. The primers used for ChIP-qPCR and antibodies used in ChIP assay were listed in Supplementary Table S3.

**In vitro ubiquitination assay**

Cells were transfected with various combinations of plasmids or antisense oligonucleotides (ASOs) along with HA-tagged ubiquitin (Ub). At 24 h after transfection, the cells were treated with MG132 (10uM, C3348-5, APExBIO) for 6h, and the whole-cell lysates prepared with NP-40-containing lysis buffer were subjected to immunoprecipitation for exogenous FLAG-tagged FUBP1 proteins. FUBP1 ubiquitination was detected by immunoblotting using an anti-FLAG antibody.

**Luciferase reporter assay**

The cells (1 × 10^3^) were seeded in triplicate in 48-well plates and allowed to settle for 24 h. One hundred nanograms of luciferase reporter plasmids or the control-luciferase plasmid, plus 5 ng of pRL-TK renilla plasmid (Promega, Madison, WI), were transfected into indicated cells using the Lipofectamine 3000 reagent (Invitrogen, Carlsbad, CA, USA) according to the manufacturer’s recommendation. Luciferase and renilla signals were measured at 48 h after transfection using the Dual Luciferase Reporter Assay Kit (Promega, Madison, WI) according to a protocol provided by the manufacturer.

**Western blotting**

Western blot analysis was performed following the previously described method^[1]^. Briefly, cells or tissues were lysed in RIPA buffer (Beyotime, China) supplemented with protease and phosphatase inhibitors (Roche, Switzerland). Protein concentration was determined using the BCA Protein Assay Kit (Thermo Fisher Scientific, USA). Equal amounts of protein (30–50 μg per lane) were separated by SDS-PAGE on 8–12% polyacrylamide gels and transferred onto PVDF membranes (Millipore, USA). Membranes were blocked with 5% non-fat milk in Tris-buffered saline containing 0.1% Tween-20 (TBST) for 1 hour at room temperature, followed by incubation with primary antibodies and loading control overnight at 4 °C. After washing with TBST, membranes were incubated with horseradish peroxidase (HRP)-conjugated secondary antibodies (Cell Signaling Technology, USA) for 1 hour at room temperature. Protein bands were visualized using an enhanced chemiluminescence (ECL) detection system (GE Healthcare, UK), and band intensities were quantified using Image J software. Results were normalized to the loading control and expressed as relative protein expression levels. The primary antibodies used in this assay are as follows: anti-FUBP1(Abcam, ab213525, 1:1000), anti-JTV-1 (Proteintech, 10424-1-AP, 1:2000), anti-TNFSF14 (Affinity Biosciences, #AF0329), anti-KDM5C (Bethyl, A301-035A, 1:3000), anti-TET1 (CST, #40142, 1:1000), anti-FLAG (Proteintech, 66008-4-Ig, 1:5000), anti-HA (Proteintech, 51064-2-AP, 1:5000) and anti-Myc (Proteintech, 60003-2-Ig, 1:5000). Anti-GAPDH (Proteintech; 60004-1, 1:20,000) antibodies were used as internal controls.

**Quantitative real-time reverse transcription PCR**

According to the manufacturer's instructions, total RNA was extracted using TRIzol, and reverse transcription was performed using TaqMan reverse transcription reagents. Real-time quantitative PCR was conducted on the ViiA7 Real-Time PCR System (Applied Biosystems, Foster City, CA, USA) using cDNA as the template and SYBR Green Master Mix. GAPDH was used as an internal control. Gene expression was evaluated by the threshold cycle (CT), and the relative expression levels were calculated using the 2^-ΔΔCT^ method. The primers were listed in the Supplementary Table S4.

**Micro-CT (*μ*CT) analysis**

After hind limbs were fixed, high-resolution images (spatial resolution 10μm) were scanned using a micro-CT scanner (SIEMENS, Munich, Germany). The purpose of the scans was to measure osteolysis of the hindlimb by determining several parameters, including bone volume fraction/total volume (BV/TV), trabecular number (Tb.N), trabecular thickness (Tb.Th), trabecular separation (Th.Sp). The three-dimensional model was reconstructed using NRecon software. After digitally eliminating cortical bone, the trabecular volume of interest was determined. The volume and number of trabecular in this area were assessed.

**Protein stability assay**

Protein stability assay was performed to evaluate the degradation kinetics of target protein. Briefly, cells were seeded in 6-well plates and treated with the protein synthesis inhibitor cycloheximide (CHX, 50 μg/mL) to block new protein production. At indicated time points (0, 2, 4, 6, 8, and 10 hours), cells were harvested and lysed in RIPA buffer supplemented with protease and phosphatase inhibitors. Equal amounts of protein (30–50 μg per lane) were separated by SDS-PAGE and transferred onto PVDF membranes. After blocking with 5% non-fat milk in TBST, membranes were incubated overnight at 4 °C with primary antibodies against target proteinp and loading control. Following incubation with HRP-conjugated secondary antibodies, protein bands were visualized using an ECL detection system. Band intensities were quantified using ImageJ software, normalized to the loading control, and expressed as a percentage of the initial level (0-hour time point). The half-life of the target protein was estimated by fitting the data to an exponential decay model.

**DNA** **fluorescence in situ hybridization (FISH)**

DNA FISH was performed using the following probes: a mixture of KDM5C/CEPX probes contained home-brewed KDM5C DNA probes labeled with SpectrumOrange (Vysis, Inc.), and chromosome X control probes labeled with SpectrumGreen (Vysis, Inc.). Tissue sections were deparaffinized, boiled, and digested, followed by hybridization with the FISH probes and sealing of the slides with rubber cement. Post-denaturation, slides were incubated overnight at 37°C, washed five times in 2× SSC buffer containing 0.3% NP-40, and counterstained with DAPI to stain the nuclei. Copy number gain of the KDM5C gene by FISH was defined as a KDM5C/CEPX ratio > 1 and KDM5C gene copy number ≥ 2. Both criteria must be met to exclude samples where a KDM5C/CEPX ratio > 1 might result solely from CEPX loss.

**Enzyme linked immunosorbent assay (ELISA)**

The LIGHT levels in both the CM of PCa cells and the serum of PCa patients were detected using the LIGHT/TNFSF14 Human ELISA Kit (Abclonal, RK10100-96T). In brief, 100 μL of each sample or standard was added to the corresponding wells of a pre-coated 96-well microplate and incubated at room temperature for 2 hours. After washing with wash buffer, biotinylated detection antibody was added and incubated for 1 hour, followed by streptavidin-HRP conjugate for an additional 30 minutes. Subsequently, TMB substrate solution was added, and the reaction was stopped after 15 minutes by adding stop solution. The absorbance was measured at 450 nm using a microplate reader (BioTek, USA). Data were collected at 450nm using the SpectraMax i3x Multi-Mode Microplate Reader (Molecular Devices), and the concentrations of LIGHT in the samples were inferred by plotting the absorbance against the corresponding concentrations of standards.

**Bone marrow macrophage (BMM) isolation**

BMMs were isolated from 6-week-old mice as previously described^[2]^. Briefly, bone marrow cells were flushed from the femur and tibia and thenresuspended in red blood lysis buffer (Beyotime, C3702-120 ml) for 2 minto remove red blood cells. The cells were cultured in complete α-MEM(Gibco, 22561-021) with 30 ng/ml M-CSF (R&D Systems, 416-ML-050) in a

suspension culture dish (Corning, 430591) at 37 °C for 3 days. After that, nonadherent cells were removed by washing, and the attached BMMs were released using 0.25% trypsin-EDTA (Thermo Fisher, 25200056) for the indicated experiments.

**Bone resorption assay *in vitro***

Bone resorption assay was performed using BONE RESORPTION ASSAY KIT (CSR-BRA-48KIT, Cosmobio). The RAW264.7 cells were inoculated on fluorescein-labeled CaP-coated 24-well plates at a density of 1x10^4^ cells/well and incubated in 1ml of conditioned medium (without phenolred). After 6 days, 100 ul of the culture supernatant was harvested into a 96-well plate and the fluorescence intensity was measured with excitation and emission wavelengths of 485 and 535nm, respectively according to the manufacturer’s instruction. Calcium phosphate that has been laid on the plate is first combined with fluorescein labeled chondroitin sulfate (FACS). After adding osteoclast, FACS are released from calcium phosphate layer into the medium through cell respiration, and the fluorescence intensity in the medium is measured to evaluate bone resorption.

For bone resorption pit assay, 1x10^5^ RAW264.7 cells were seeded onto bone slices (DT-1BON1000-96; IDSPLC, Boldon, T&W) in 24-well plate and added to CM from PCa cells receiving the indicated treatments supplemented with 25ng/ml murine RANKL in a humidified atmosphere of 5% CO_2_ at 37 °C. The cultures were maintained for 9 days, with the CM (2ml/well) being replaced every other day. After 9 days, the bone slices were fixed with 2.5% glutaraldehyde, and the cells were removed by mechanical agitation and ultrasound. Subsequently, bone resorption areas were observed using scanning electron microscopy, and three random fields of view were selected for further analysis. ImageJ software was used to quantify the bone resorption pit area.

To investigate vicious cycle affected by osteoclast differentiation, above RAW264.7 cells seeded onto bone slices were treated with CM from PCa cells for 9 days. Then, the conditional medium was removed and the cells were cultured in fresh medium for 24 hours. The culture medium was then collected to measure the concentration of TGF-β. In addition, the collected medium was used as conditioned medium to treat prostate cancer (PCa) cells. Following three days of culture, EdU staining was performed to evaluate the proliferative capacity of PCa cells.

**RNA pull-down assay**

Biotinylated sense and antisense CCDC183-AS1 probes were synthesized from Exon Biotech (Guangzhou). Firstly, probes were incubated with streptavidin magnetic beads at room temperature for 1 hour and the mixture is then incubated overnight at 4°C with PCa cell lysate. Following this, the RNA-protein complex is washed three times to remove unbound components, and the proteins are eluted from the beads. The eluted proteins are subsequently separated by SDS-PAGE and analyzed using either Western blotting or LC-MS/MS.

**In situ hybridization (ISH)**

ISH staining and scoring were performed on the tissue samples as previously described^[3]^. A biotin-labeled CCDC183-AS1 probe was synthesized by Sangon Biotech (Shanghai, China). Briefly, ISH staining was performed to detect CCDC183-AS1 expression in paraffin-embedded PCa tissues. DAB Enhanced Liquid Substrate System (Sigma, Chicago, IL, USA) was used for staining. Staining scores (SI) were assessed by two independent pathologists to comparatively evaluate the expression of CCDC183-AS1. Staining index (SI) given by the two independent investigators were averaged for further comparative evaluation of CCDC183-AS1 expression. Tumor cell proportion was scored as follows: 0 (no positive tumor cells); 1 (< 10% positive tumor cells); 2 (10–35% positive tumor cells); 3 (35–70% positive tumor cells) and 4 (> 70% positive tumor cells). Staining intensity was graded according to the following criteria: 0 (no staining); 1 (weak staining, light yellow); 2 (moderate staining, yellow brown) and 3 (strong staining, brown). SI was calculated as the product of staining intensity score and the proportion of positive tumor cells. Based on this method of assessment, CCDC183-AS1 expression in prostate tumor samples was evaluated by the SI, with scores of 0, 1, 2, 3, 4, 6, 8, 9 or 12. SI score 4 was the median of all sample tissues SI. High and low expression of CCDC183-AS1 were stratified by the follow criteria: The SI ≤4 was used to define tumors with low expression of CCDC183-AS1, and SI score of > 6 as tumors with high expression of CCDC183-AS1.

**RNA fluorescence in situ hybridization**

The Cy3-labeled probe targeting the CCDC183-AS1 sequence was synthesized by RiboBio (Guangzhou, China). A fluorescence in situ hybridization (FISH) kit (RiboBio, Guangzhou, China) was used to detect probe signals in prostate cancer cells. Briefly, cells were fixed with paraformaldehyde for 10 minutes at room temperature, then permeabilized with 1 ml of 70% ethanol for 1 h at 4°C. Subsequently, an anti-CCDC183-AS1 oligonucleotide probe set was used for hybridization. After hybridization, the nuclei were stained with 4′,6-diamidino-2-phenylindole (DAPI). Representative images were obtained using an LSM810 confocal microscope (Carl Zeiss, Oberkochen, Germany).

**Proximity ligation assay (PLA)**

Proximity ligation assay (PLA) was performed using a NaveniFlex Cell MR RED KIT (#NC.MR.100.Red, Navinci Diagnostics) according to the manufacturer's instructions. After blocking non-specific binding, the primary antibody was added and incubated overnight at 4°C. Subsequently, Navenibody incubation, DNA circle formation, amplification, and DAPI staining were performed according to the manufacturer's instructions. Finally, the fluorescent signals were detected using a confocal microscope (Carl Zeiss, Oberkochen, Germany). The primary antibodies used in this assay are as follows: anti-FUBP1(Santa Cruz, sc-271241), anti-JTV1(Abcam, ab228004), anti-KDM5C (ZenBio, #200054), anti-TET1(Abcam, ab272900).

**Immunofluorescence (IF) staining**

Immunofluorescence (IF) staining was performed as previously described^[4]^. Briefly, the PCa cells were seeded onto coverslips. The cells were then fixed with 4% paraformaldehyde for 15 min and washed thrice with PBS. Cells were neutralized with 5% glycine for 5 min and permeabilized with 5% Triton X-100 for 15 min. After blocking with 10% goat serum for 1 h, the primary antibody (FUBP1, Abcam, ab213525) was added and incubated overnight at 4°C. The secondary antibody was then added and incubated at room temperature for 1 h, followed by three washes with PBS. Nuclei were stained with DAPI for 5 min and washed three times with PBS. Finally, the coverslips were mounted onto slides and images were obtained using a confocal microscope (Carl Zeiss AG, Oberkochen, Germany).

**Hematoxylin-eosin (H&E) and TRAP staining**

After fixing the femurs and tibias in paraformaldehyde solution (4%) overnight, they were decalcified using 0.5M EDTA (G1105, Servicebio) for 2 weeks and then embedded in paraffin. The paraffin sections were stained with a hematoxylin-eosin (H&E)(G1005; Servicebio) or TRAP staining kit (G1050-50T; Servicebio) according to the manufacturer's protocol. TRAP^+^-osteoclasts were counted on a 3 mm length of endocortical surface and observed on an optical microscope (Olympus, Tokyo, Japan)

**Osteoblastic Differentiation Assay**

Murine osteoblastic MC3T3-E1 cells were purchased from Guangzhou Jennio Biotech Co., Ltd.(Guangzhou, China) and cultured with Dulbecco’s modified eagle medium (DMEM, Gibco, USA), supplemented with 10% fetal bovine serum (FBS, Cell-Box, HongKong, China) and 100 U/mL penicillin-streptomycin (Hyclone, Logan, UT, USA) in an incubator with 5% CO2 at 37 °C. For osteogenic differentiation, 10 mM β-glycerolphosphate disodium salt hydrate (Sigma-Aldrich, St. Louis, MO, USA), 10 nM dexamethasone (Sigma-Aldrich, St. Louis, MO, USA), and 50 μg/mL L-ascorbic acid (Sigma-Aldrich, St. Louis, MO, USA) were added to complete the DMEM medium derived from PCa cells to the prepare osteogenic medium. The induction medium was refreshed every 2 days. After 2 weeks of induction, Alkaline phosphatase (ALP) staining was performed to assess osteogenic differentiation.

**Alkaline phosphatase (ALP) staining**

Fourteen days after osteogenic induction, a TRAP/ALP Stain Kit (Wako, Richmond, VA, USA; 294-67001) was used to perform alkaline phosphatase (ALP) staining. The cells cultured in 24-well plates were rinsed three times by PBS and fixated for 30 min using pre-cold fixative. Next, ALP substrate solution was added and cells were cultivated (room temperature, 15-45min) in a darkroom. After that, cells with ddH_2_O were rinsed and filmed under an optical microscope. The absorbance at 405nm of each well was measured with a microplate reader according to the manufacturer’s instruction.

**In vitro methylation**

A 155 bp fragment of the CCDC183-AS1 promoter region (-1728 to -1574 bp) was obtained from whole gene synthesis (BGI Genomics, Beijing, China). SssI, HpaII, and HhaI methylases (New England Biolabs) were used to perform methylation in vitro, according to the manufacturer's instructions. All cytosine residues within 5'-CpG-3' sequences are methylated by SssI, 5'-CCGG-3' sequences are methylated by HpaII, and 5'-GGCG-3' sequences are methylated by HhaI. Complete methylation was confirmed after digestion with HhaI, HpaII, or MCBC (New England Biolabs). Methylated or mock-methylated fragments were ligated into pGL3-basic vectors (Promega) for the luciferase reporter gene experiments.

**Bisulfite sequencing PCR (BSP) and methylation-specific PCR (MSP)**

The methylation status of the CCDC183-AS1 promoter was analyzed using BSP and MSP. Genomic DNA was isolated from cultured cells using a DNeasy Blood & Tissue Kit (#69504, Qiagen), and cell-free DNA was extracted from serum samples using a QIAamp DNA Blood Mini Kit (#61104, Qiagen). The isolated DNA was then subjected to bisulfite conversion and purification using EpiTect Bisulfite Kits (#59104, Qiagen) following the manufacturer's instructions. For the BSP, PCR primers were designed to amplify the modified CCDC183-AS1 promoter. The amplified products were purified using a QIAquick Gel Extraction Kit (#28704, Qiagen), cloned into the pMD19-T vector (#6013, Takara), and sequenced. For each sample, five independent clones were analyzed to determine the methylation status, and the average methylation frequency was calculated. MSP was conducted using an EpiTect MSP kit (Qiagen) according to the manufacturer’s protocol. The PCR products were separated on 2% agarose gels, stained with DuRed, and visualized under UV light. Fully methylated and unmethylated DNA fragments from the EpiTect PCR Control DNA Set (Qiagen) were used as positive and negative controls, respectively. For Methylation-Specific Quantitative PCR (MS-qPCR) assays, the amplified modified DNA was utilized to ascertain the methylation status of the promoter region of the target gene, as outlined in prior literature^[5]^. The sequences of the primers are listed in Supplementary Table S5.

**Immunohistochemistry (IHC)**

Immunohistochemical detection of the target protein was conducted following previously described methods^[1]^. Formalin-fixed paraffin-embedded (FFPE) tumor tissues were sectioned into 4μm slices, followed by immunohistochemical staining with primary antibodies. Two independent observers evaluated and scored the immunostaining intensity of the indicated protein, scoring both the proportion of positively stained tumor cells and the staining intensity. The IHC scores were recorded as the average of selected fields, calculated as the staining intensity score multiplied by the proportion score of positive tumor cells. Staining intensity scores were assigned as follows: 0 (no staining), 1 (pale yellow), 2 (yellow-brown), 3 (brown). Proportion scores for positive tumor cells were assigned as follows: 0 (no positive tumor cells), 1 (<10% positive tumor cells), 2 (10-35% positive tumor cells), 3 (36-70% positive tumor cells), 4 (>70% positive tumor cells). Based on the IHC scores, target gene expression was defined as follows: negative staining (0 score), weak staining (1-4 score), moderate staining (5-8 score), strong staining (9-12 score). The median value of the target gene's IHC score was used as the cutoff value to stratify into high and low-expression groups. The primary antibodies used in this assay are as follows: anti-FUBP1(Abcam, ab213525), anti-TNFSF14 (Affinity Biosciences, #AF0329), anti-KDM5C (Affinity Biosciences, DF13631).

**EdU assay**

The cell proliferation capacity was assessed using the EdU Cell Proliferation Detection Kit (RiboBio, Guangzhou). Briefly, cells were seeded in 96-well plates or on glass coverslips and allowed to attach overnight. Following treatment or transfection as indicated, cells were incubated with 10 μM EdU labeling medium for 2 hours at 37 °C to allow incorporation of EdU into newly synthesized DNA. After fixation with 4% paraformaldehyde for 15 minutes and permeabilization with 0.5% Triton X-100 in PBS, cells were subjected to a copper-catalyzed azide-alkyne cycloaddition reaction with Alexa Fluor® 488 or 594 azide for detection of EdU-positive cells. Nuclei were counterstained with Hoechst 33342 or DAPI. Fluorescent images were captured using a fluorescence microscope or confocal microscope (Olympus, Japan), and the percentage of EdU-positive cells was quantified using ImageJ or CellProfiler software

**Mass spectrometry**

Protein partners of CCDC183-AS1 were identified through liquid chromatography-tandem mass spectrometry (LC-MS/MS). The Q-Exactive system from ThermoFisher Scientific, featuring a nano electrospray ion source and a nanoViper C18 chromatography column, was employed to analyze peptides derived from differential expression bands detected via Coomassie Brilliant Blue staining. The mass spectrometer operated in full MS mode with a resolving power of 70,000 at m/z 400, scanning a mass range from 350 to 2,000 m/z with a maximum ion fill time of 50 ms. All identified peptides underwent meticulous manual inspection, with spectra interpreted for accurate identification.

**High throughput data processing**

The clinical profile of PCa dataset, RNA sequencing profile were downloaded from The Cancer Genome Atlas (TCGA; https://tcga-data.nci.nih.gov/tcga/) and Gene Expression Omnibus (GEO; https://www.ncbi.nlm.nih.gov/geo/) database. TCGA database is a publicly available resource that provides multi-dimensional genomic data across various cancer types. In this study, RNA expression data and corresponding clinical information for PCa were downloaded from TCGA using the GDC Data Portal (https://portal.gdc.cancer.gov). GEO is a public functional genomics data repository maintained by the National Center for Biotechnology Information (NCBI). We selected relevant datasets based on the following criteria: (1) studies involving PCa, (2) containing gene expression profiles, and (3) availability of clinical or phenotypic information. Raw data were downloaded and normalized using R/Bioconductor packages, and probe-level data were converted into gene-level expression values according to the corresponding platform annotations. The copy number data of PCa patients was downloaded from cBioPortal (https://www.cbioportal.org/).

[1] C. Yin, M. Wang, Y. Wang, Q. Lin, K. Lin, H. Du, C. Lang, Y. Dai, X. Peng, *Journal for immunotherapy of cancer,* **2023,** *11,*

[2] K. Chen, X. Chen, C. Lang, X. Yuan, J. Huang, Z. Li, M. Xu, K. Wu, C. Zhou, Q. Li, C. Zhu, L. Liu, X. Shang, *Experimental & molecular medicine,* **2023,** *55,* 2051-2066.

[3] C. Lang, Y. Dai, Z. Wu, Q. Yang, S. He, X. Zhang, W. Guo, Y. Lai, H. Du, H. Wang, D. Ren, X. Peng, *Molecular oncology,* **2020,** *14,* 808-828.

[4] H. Tian, R. Lian, Y. Li, C. Liu, S. Liang, W. Li, T. Tao, X. Wu, Y. Ye, X. Yang, J. Han, X. Chen, J. Li, Y. He, M. Li, J. Wu, J. Cai, *Nat Commun,* **2020,** *11,* 5127.

[5] M. W. Chan, E. S. Chu, K. F. To, W. K. Leung, *Biotechnol Lett,* **2004,** *26,* 1289-93.

**Supplemental Tables**

**Supplementary Table S1. The 32 commonly observed bone-remodeling factors.**

| **GM-CSF** | **M-CSF** | **RANKL** | **OPG** | **IL1** | **IL3** |
| --- | --- | --- | --- | --- | --- |
| **IL6** | **IL8** | **IL11** | **TRAIL** | **LIGHT** | **APRIL** |
| **TNF** | **BAFF** | **NGF** | **VEGF** | **CTGF** | **PTHRP** |
| **HGF** | **MIP1α** | **DKK1** | **BMP4** | **OPN** | **JAGGED1** |
| **IGF1** | **IGF2** | **HPSE** | **GDF15** | **FGF9** | **MMP1** |
| **LGALS3** | **CCL2** |  |  |  |  |

**Supplementary Table S2. The sequences of shRNAs and ASOs.**

| **shRNA Sequence (5’-3’)** | |
| --- | --- |
| KDM5C | CCCACTACGAACGCATTGTTT |
| TET1 | CCCAGAAGATTTAGAATTGAT |
| FUBP1 | CCCGAAAGGATAGCACAAATA |
|  |  |
| **ASO** | **Sequence (5’-3’)** |
| CCDC183-AS1 #1 | CAGTAGGAAGTCAACAAAGAA |
| CCDC183-AS1 #2 | TGGATCTTGTCGATCAGCTTC |
|  |  |

**Supplementary Table S3. List of primers used for RT-qPCR.**

| **Primer Sequence** | |
| --- | --- |
| CCDC183-AS1-F | GACTTGATCCGTTGGCCTGA |
| CCDC183-AS1-R | CTTGGACTTCCCCTCGAACC |
| FUBP1-F | AACAGGACCTCCAGACCGATGT |
| FUBP1-R | TCCAGTTGCCTTGACCTCTACC |
| GAPDH-F | GTCTCCTCTGACTTCAACAGCG |
| GAPDH-R | ACCACCCTGTTGCTGTAGCCAA |
| U6-F | CTCGCTTCGGCAGCACAT |
| U6-R | TTTGCGTGTCATCCTTGCG |
| TNFSF14-F | GGTCTCTTGCTGTTGCTGATGG |
| TNFSF14-R | TTGACCTCGTGAGACCTTCGCT |
| KDM5C-F | ACTGCTGACCATTGCTGAACGC |
| KDM5C-R | CCTCCTTGAGAGCCTGGATGTT |
| TET1-F | CAGGACCAAGTGTTGCTGCTGT |
| TET1-R | GACACCCATGAGAGCTTTTCCC |

**Supplementary Table S4. List of the primers and antibodies used for ChIP-qPCR.**

| **Primer Sequence** | |
| --- | --- |
| CCDC183-AS1 promoter | 5’-3’ |
| F | CTCCTGCAGGTGAGTGGTG |
| R | TGATTACATAATGCCAAGCAGGG |
| TNFSF14 P1 site | 5’-3’ |
| F | TTGAGTCAAGACGCAGATGAGCA |
| R | AGATATCCTGGGTCTTTGCACTTCC |
|  |  |
| **Antibody** | **Brand** |
| Flag | Proteintech, 66008-4-Ig |
| KDM5C | Bethyl, A301-035A |
| TET1 | CST, #40142 |
| H3K4me3 | CST, #9751 |
| 5mc | Active Motif, 61255 |
| 5hmc | Active Motif, 39769 |
| TET2 | Abcam, ab313548 |
| TET3 | Active Motif, #61395 |
| DNMT1 | Abcam, ab87656 |
| DNMT3A | Abcam, ab2850 |
| DNMT3B | Abcam, ab13604 |
| IgG | Santa Cruz, sc-2025 |

**Supplementary Table S5. The sequences of BSP and MSP primers.**

| **BSP primer Sequence (5’-3’)** | |
| --- | --- |
| CCDC183-AS1 |  |
| F | AGTAGGAAGTTAATAAAGAAAAATTT |
| R | AATTACATAATACCAAACAAAACCC |
|  |  |
| **MSP primer** | **Sequence (5’-3’)** |
| CCDC183-AS1 |  |
| Methylated-F | AATAAAGAAAAATTTCGTTGAAAGC |
| Methylated-R | ATTACATAATACCAAACAAAACCCG |
| Unmethylated-F | TAAAGAAAAATTTTGTTGAAAGTGT |
| Unmethylated-R | TTACATAATACCAAACAAAACCCAC |
|  |  |
