# Supplementary figures and images for: Epigenetic Activation of CCDC183‐AS1 Promotes Osteoclastogenesis and Prostate Cancer Bone Metastasis Through the FUBP1/LIGHT Axis
Source: Adv Sci (Weinh). 2025 Jul 20;12(38):e13288. doi: 10.1002/advs.202413288 (PMC12520472; doi:10.1002/advs.202413288)

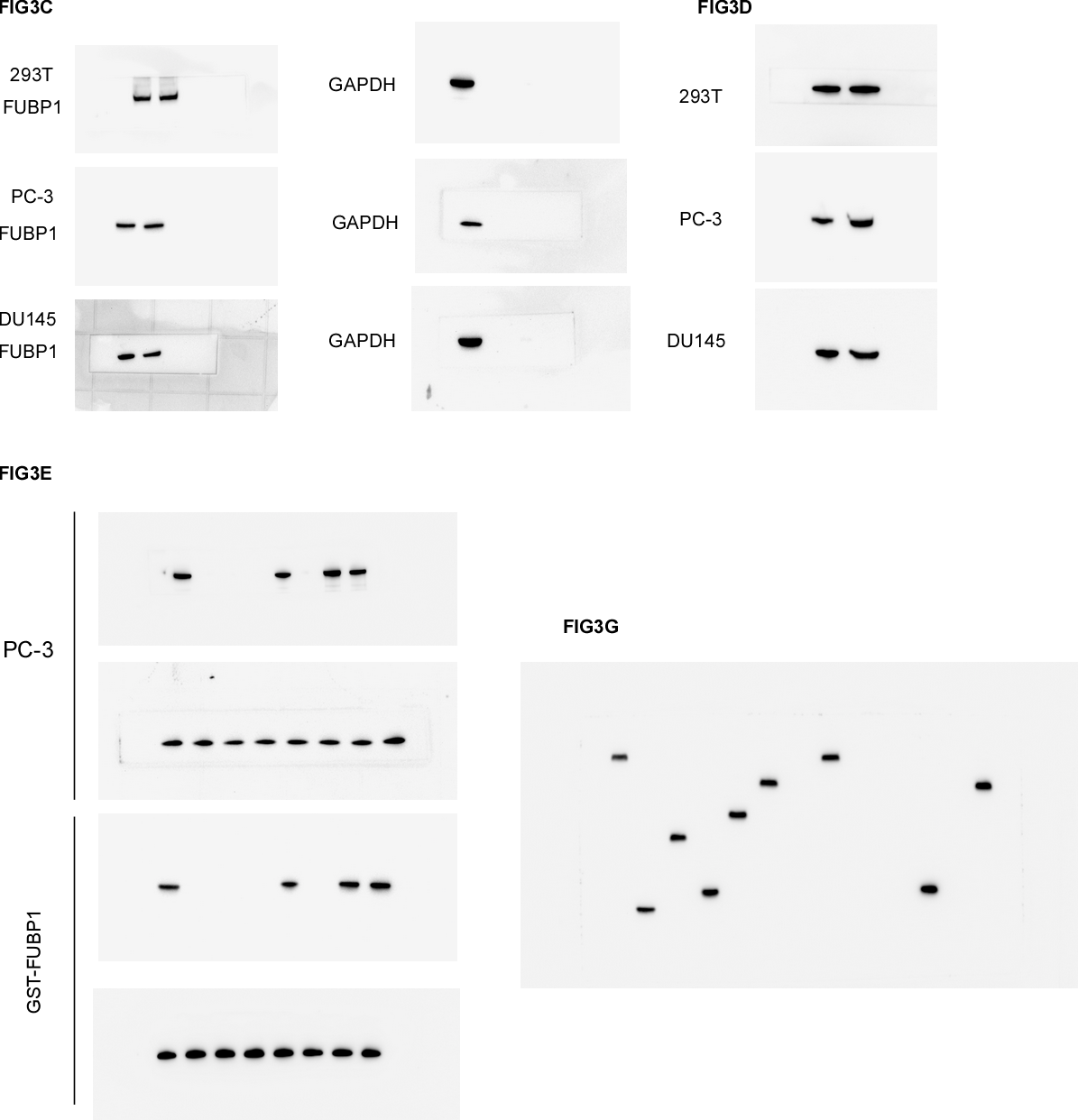


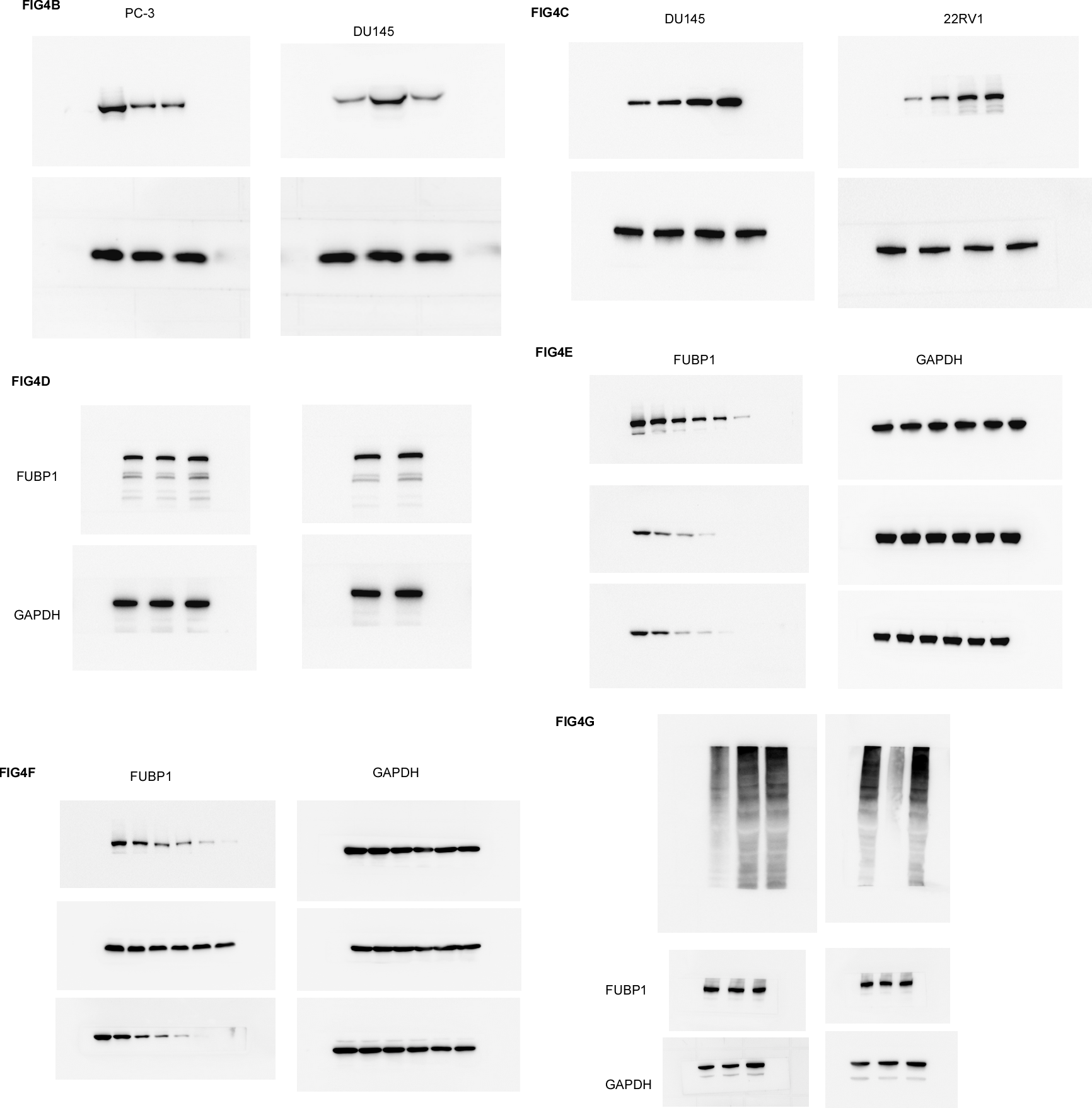


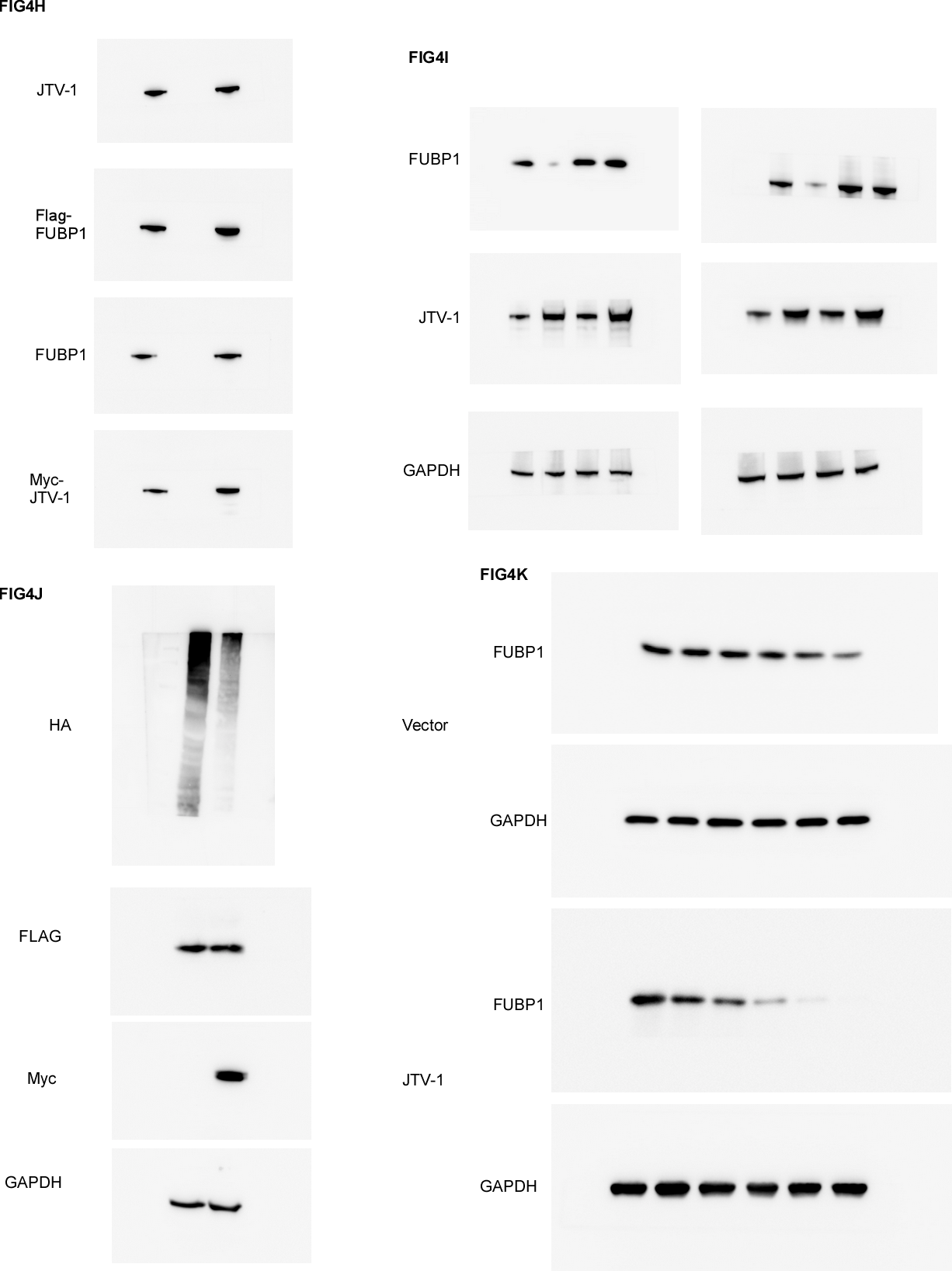


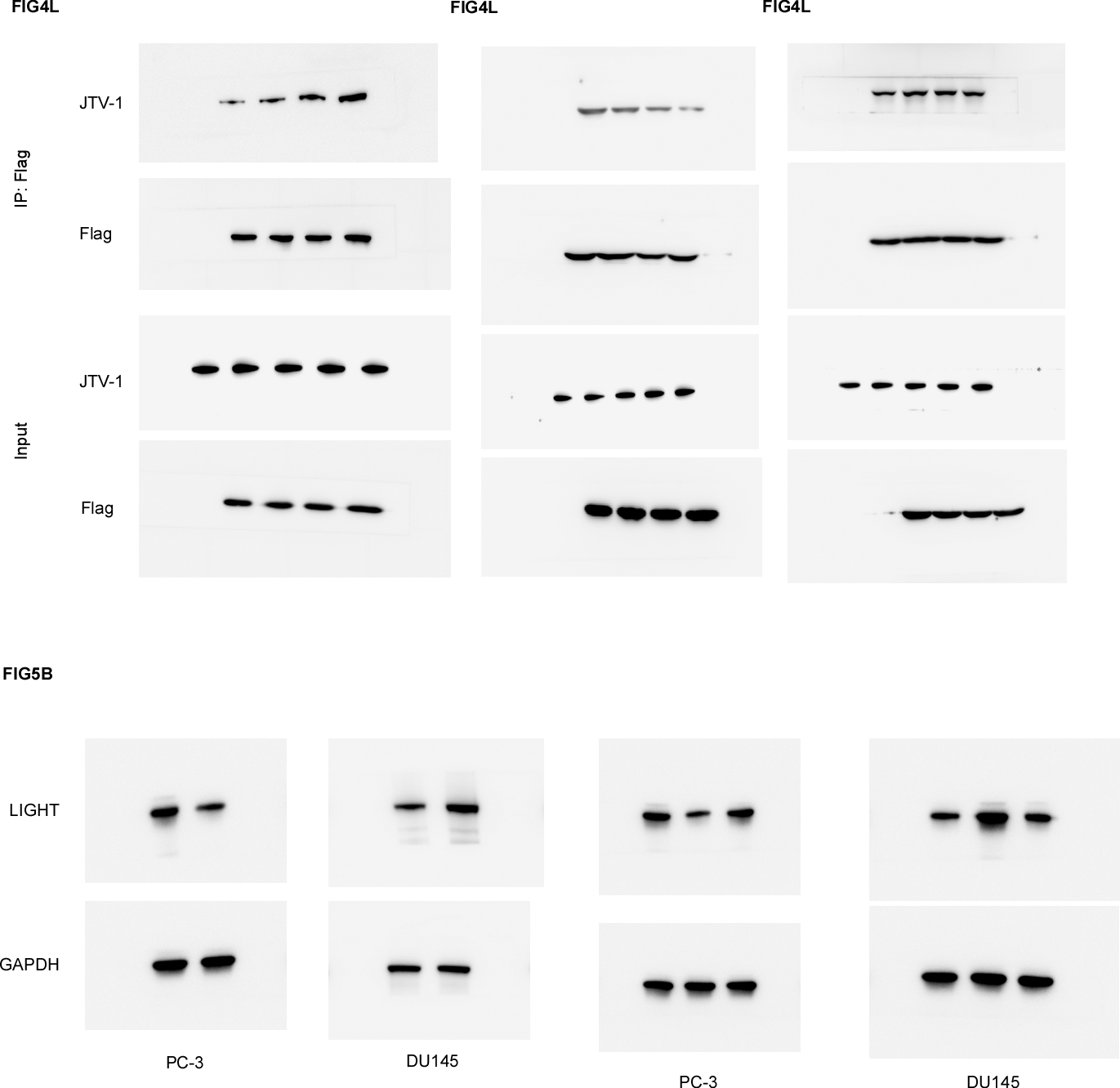


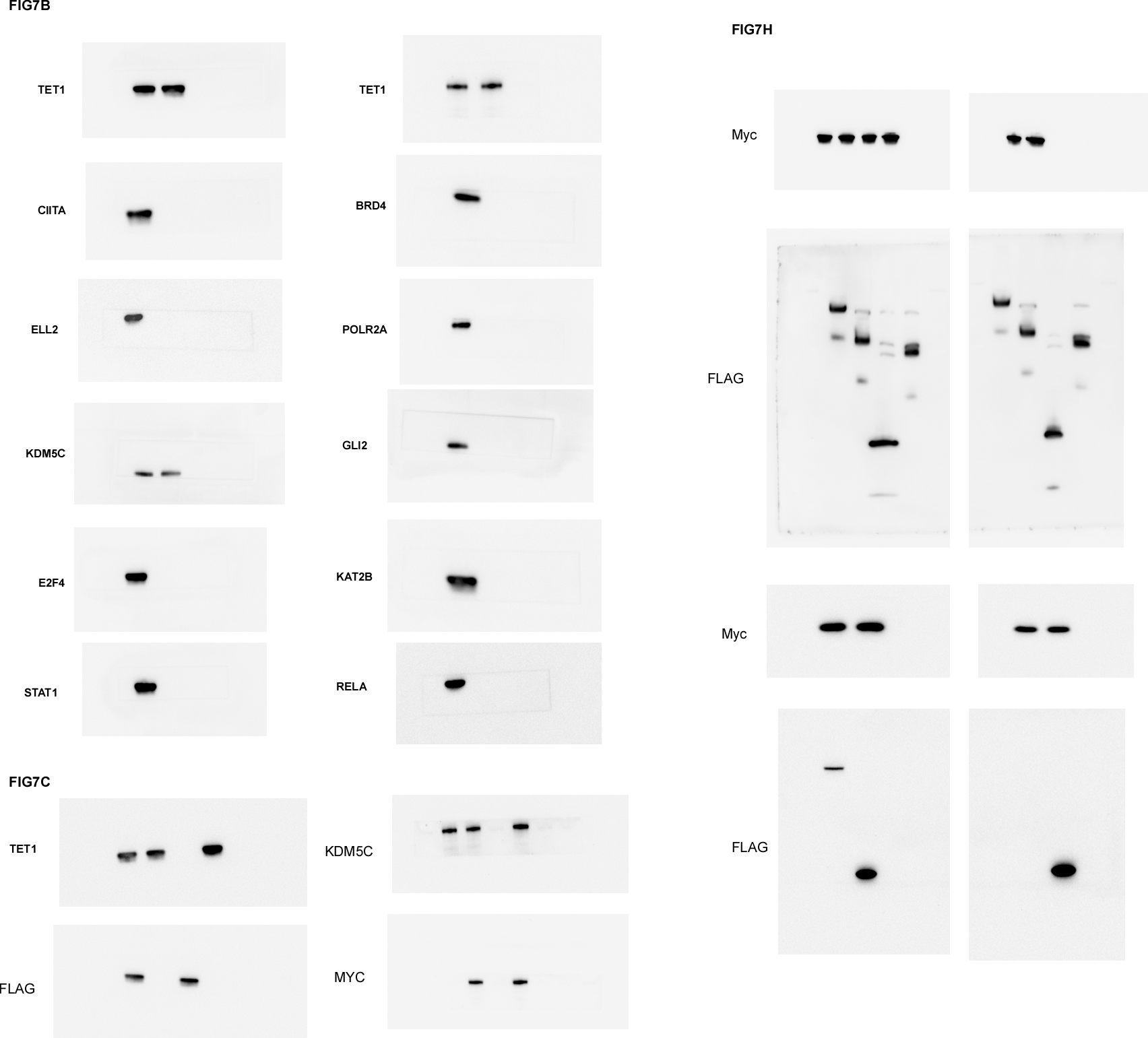


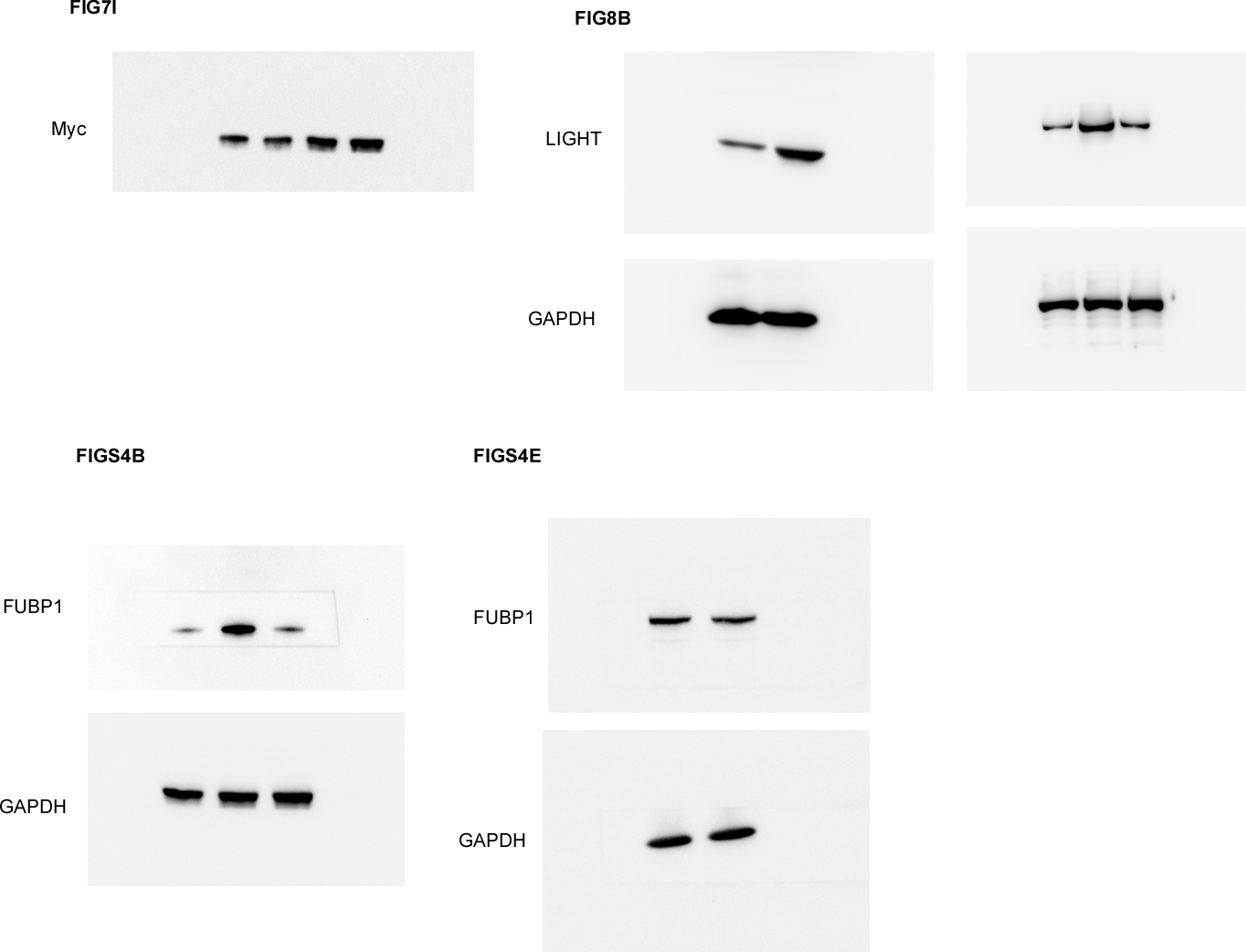


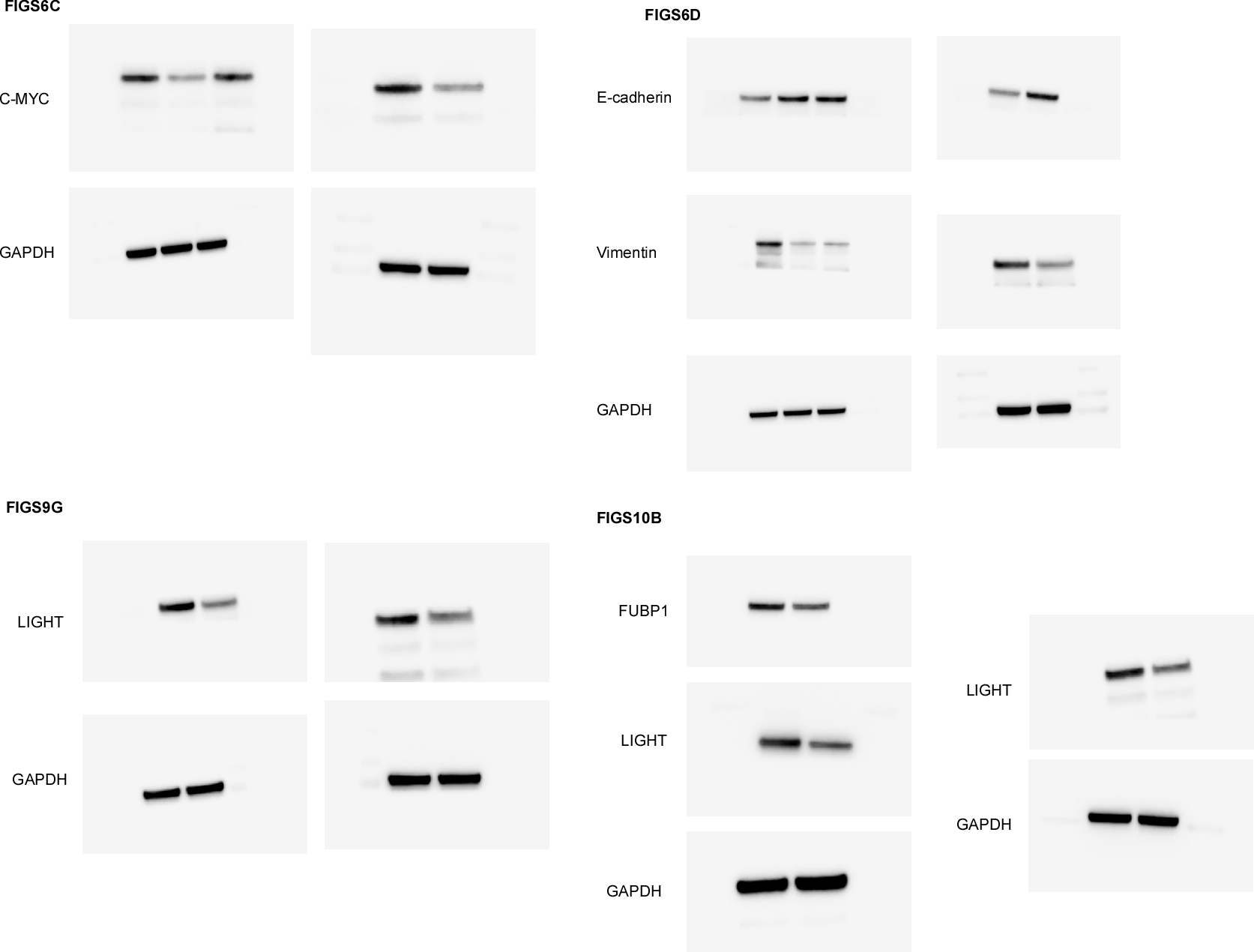

Supplement: Supplementary file 2 — Supporting Information [file ADVS-12-e13288-s002.docx]
